# Synergy between Genome Mining, Metabolomics, and Bioinformatics Uncovers Antibacterial Chlorinated Carbazole Alkaloids and Their Biosynthetic Gene Cluster from *Streptomyces tubbatahanensis* sp. nov., a Novel Actinomycete Isolated from Sulu Sea, Philippines

**DOI:** 10.1128/spectrum.03661-22

**Published:** 2023-02-21

**Authors:** Chuckcris P. Tenebro, Dana Joanne V. L. Trono, Lex Aliko P. Balida, Leah Katrine A. Bayog, Julyanna R. Bruna, Edna M. Sabido, Dion Paul C. Caspe, Emmanuel Lorenzo C. de Los Santos, Jonel P. Saludes, Doralyn S. Dalisay

**Affiliations:** a Center for Chemical Biology and Biotechnology, University of San Agustin, Iloilo City, Philippines; b Center for Natural Drug Discovery and Development, University of San Agustin, Iloilo City, Philippines; c Research Analytics, Early Solutions Data & Translational Services, UCB Celltech, Slough, Berkshire, United Kingdom; d Balik Scientist Program, Department of Science and Technology, Philippine Council for Health Research and Development, Bicutan, Taguig City, Philippines; e Department of Chemistry, College of Liberal Arts, Sciences, and Education, University of San Agustin, Iloilo City, Philippines; f Department of Biology, College of Liberal Arts, Sciences, and Education, University of San Agustin, Iloilo City, Philippines; University of Melbourne

**Keywords:** *Streptomyces*, tryptophan halogenase, flavin reductase, specialized metabolites, anticancer, antibiotic, biosynthetic gene clusters, halogenated carbazole alkaloids

## Abstract

In this study, a novel actinomycete strain, DSD3025^T^, isolated from the underexplored marine sediments in Tubbataha Reefs Natural Park, Sulu Sea, Philippines, with the proposed name *Streptomyces tubbatahanensis* sp. nov., was described using polyphasic approaches and characterized using whole-genome sequencing. Its specialized metabolites were profiled using mass spectrometry and nuclear magnetic resonance analyses, followed by antibacterial, anticancer, and toxicity screening. The *S. tubbatahanensis* DSD3025^T^ genome was comprised of 7.76 Mbp with a 72.3% G+C content. The average nucleotide identity and digital DNA-DNA hybridization values were 96.5% and 64.1%, respectively, compared with its closest related species, thus delineating the novelty of *Streptomyces* species. The genome encoded 29 putative biosynthetic gene clusters (BGCs), including a BGC region containing tryptophan halogenase and its associated flavin reductase, which were not found in its close *Streptomyces* relatives. The metabolite profiling unfolded six rare halogenated carbazole alkaloids, with chlocarbazomycin A as the major compound. A biosynthetic pathway for chlocarbazomycin A was proposed using genome mining, metabolomics, and bioinformatics platforms. Chlocarbazomycin A produced by *S. tubbatahanensis* DSD3025^T^ has antibacterial activities against Staphylococcus aureus ATCC BAA-44 and Streptococcus pyogenes and showed antiproliferative activity against colon (HCT-116) and ovarian (A2780) human cancer cell lines. Chlocarbazomycin A exhibited no toxicity to liver cells but moderate and high toxicity to kidney and cardiac cell lines, respectively.

**IMPORTANCE** *Streptomyces tubbatahanensis* DSD3025^T^ is a novel actinomycete with antibiotic and anticancer activities from Tubbataha Reefs Natural Park, a United Nations Educational, Scientific and Cultural Organization World Heritage Site in Sulu Sea and considered one of the Philippines’ oldest and most-well-protected marine ecosystems. *In silico* genome mining tools were used to identify putative BGCs that led to the discovery of genes involved in the production of halogenated carbazole alkaloids and new natural products. By integrating bioinformatics-driven genome mining and metabolomics, we unearthed the hidden biosynthetic richness and mined the associated chemical entities from the novel *Streptomyces* species. The bioprospecting of novel *Streptomyces* species from marine sediments of underexplored ecological niches serves as an important source of antibiotic and anticancer drug leads with unique chemical scaffolds.

## INTRODUCTION

Identifying novel *Streptomyces* species is crucial in the search for new specialized metabolites with antibiotic and anticancer activities. This undertaking can address the rapid emergence of multidrug-resistant pathogens (1). Members of the phylum *Actinobacteria* serve as a primary source of specialized metabolites that comprise 10% of the 211,000 described bacterial sequences in the NCBI database (2). From the 39% estimated *Actinobacteria* specialized metabolites (3), 80% were produced by members in the genus *Streptomyces*. In fact, 5 to 10% of the *Streptomyces* genome is dedicated to specialized metabolism, and most of the products remained undiscovered (4). The specialized metabolites from *Streptomyces* species are largely represented by nonribosomal peptides and polyketides (5). These compounds can have a wide array of bioactivities, such as antimicrobial, anticancer, antiparasitic, or immunosuppressant activities, or other therapeutic agents (6).

There are more than 4,700 halogenated compounds reported for which the halogen moiety is important for target specificity and enhances the compound’s pharmacological activities (7). In 2021, 14 of 50 molecules approved by the FDA were halogen-containing drugs that displayed a broad range of antibiotic and anticancer activities (8). The genus *Streptomyces* produces a diverse set of biosynthetic gene clusters (BGCs), accounting for 20 to 40 genomic regions for specialized metabolites biosynthesis (9) which conventional strategies fail to uncover. The advancements in genomics, bioinformatics, and metabolomics have accelerated the identification and isolation of chemically diverse natural products (10). Some of the well-known chlorinated specialized metabolites derived from *Streptomyces* are the antibiotics vancomycin (11) and chloramphenicol (12), the antitumor agent rebeccamycin (13), and the antifungal agent pyrrolnitrin (14).

Traditionally, compounds from a bioactive microbial strain were isolated by bioassay-guided approaches (15, 16). Meanwhile, bioinformatics-driven predictions using whole-genome sequencing have uncovered the core BGCs and cryptic metabolites, followed by the integration of high-resolution metabolomics (17) for specialized metabolite identification. The genome-based bioprospecting (17) and metabolomics-enabled approaches (18) opened a new era in natural product drug discovery and development.

From January 2015 to December 2020, there was a rising number of *Streptomyces* species isolated from different ecological niches (19). During these 6 years, 135 new *Streptomyces* species were recovered from terrestrial (*n* = 108) and marine (*n* = 27) sources, highlighting 121 *Streptomyces* species as prolific producers of 279 new specialized metabolites with wide pharmaceutical applications (19). In the year 2020 alone, 74 novel specialized metabolites were identified from *Streptomyces* species, with the largest portion isolated from marine-derived *Streptomyces* (20). From 2015 to 2020, 71% of the novel marine-derived *Streptomyces* species were recovered from marine sediments (19). These reports demonstrated that novel *Streptomyces* species from marine sediments play a significant role as sources of new bioactive specialized metabolites.

In the last 5 years, our laboratory has explored the marine sediments of the Philippine archipelago for antibiotic- and anticancer-producing *Streptomyces* and has reported the bioactive specialized metabolites they produce (15, 21–23). In the present study, a novel *Streptomyces* strain isolated from marine sediments of Tubbataha Reefs Natural Park in the middle of Sulu Sea, Philippines, was investigated for its phylogenetic, phenotypic, chemotaxonomic, and genomic features, based on its whole-genome sequence. Based on this array of testing and data analyses, marine sediment-derived actinomycete strain DSD3025^T^ is proposed to represent a novel *Streptomyces* species named *Streptomyces tubbatahanensis* sp. nov. (DSD3025^T^ = DSM 33792^T^). *S. tubbatahanensis* DSD3025^T^ was part of the over 2,000 Actinobacteria library collection reported from the marine sediments collected from different sampling sites in the Philippine archipelago (21).

The antibiotic and anticancer potentials of *S. tubbatahanensis* DSD3025^T^ were explored via genome mining, metabolite profiling, bioinformatics, and bioassays. The *S. tubbatahanensis* DSD3025^T^ genome encodes 29 putative BGCs, including a BGC of interest that contains tryptophan halogenase and flavin reductase genes, a two-component halogenase/reductase system. The bioinformatics analysis and BGC evaluation predicted compounds belonging to PKS I, PKS II, and NRPS moieties. Metabolite profiling of *S. tubbatahanensis* DSD3025^T^ crude extract by using ultra performance liquid chromatography-electrospray ionization–time of flight mass spectrometry (UPLC-ESI-TOF-MS) and nuclear magnetic resonance (NMR) analysis identified chlocarbazomycin A compound **1**, a recently discovered halogenated carbazole alkaloid, from its closely related *Streptomyces* species as a metabolite. Aside from compound **1**, several halogenated compounds were produced by *S. tubbatahanensis* DSD3025^T^. The extract induced cell death via membrane damage of multidrug-resistant Staphylococcus aureus ATCC BAA-44 and inhibited growth proliferation of MCF-7, HCT-116, and A2780 cancer cell lines. Moreover, compound **1** had antibacterial activity against S. aureus ATCC BAA-44 and Streptococcus pyogenes and showed antiproliferative activity against HCT-116 and A2780 cancer cell lines. Based on genome annotation data and bioinformatics analysis, we have propose the biosynthetic pathway of compound **1** in *S. tubbatahanensis* DSD3025^T^.

## RESULTS AND DISCUSSION

### Phylogenetic analysis of *S. tubbatahanensis* DSD3025^T^.

**FIG 1:**
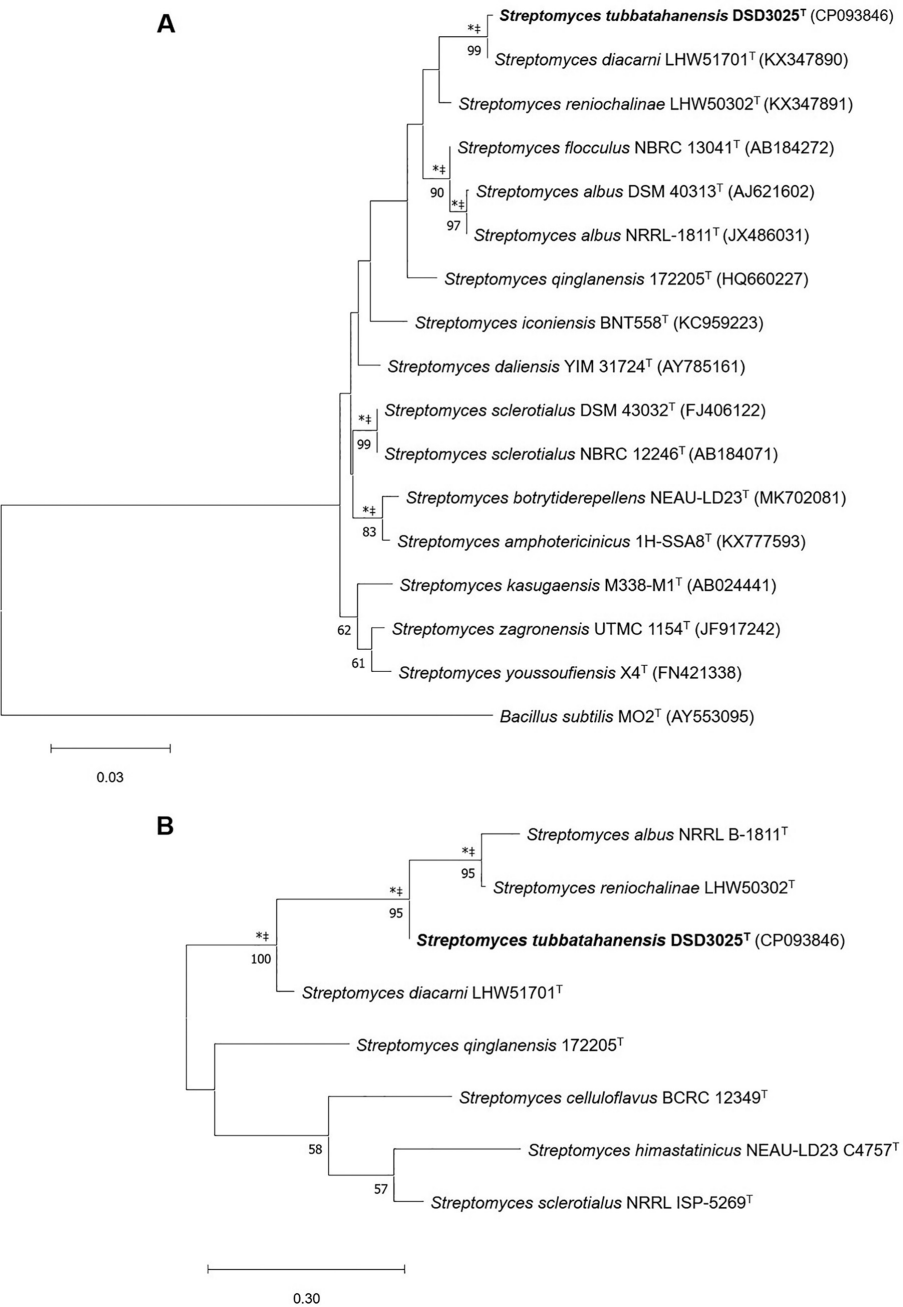
Maximum-likelihood tree showing phylogenetic relationships between *S. tubbatahanensis* DSD3025^T^ and related *Streptomyces* species, based on 16S rRNA gene sequences (A) and concatenated five housekeeping genes (*atpD*, *gyrB*, *rpoB*, *recA*, and *trpB*) (B). Only bootstrap values above 50% (percentages of 1,000 replications) are indicated. Asterisks and crosses indicate branches that were also found using neighbor-joining and the maximum-parsimony method, respectively.

The complete 16S rRNA gene sequence of *S. tubbatahanensis* DSD3025^T^ (1,531 bp) was obtained from its whole-genome sequence. *S. tubbatahanensis* DSD3025^T^ shares 16S rRNA gene sequence similarity with *Streptomyces* species, particularly Streptomyces diacarni LHW51701^T^ (99.73%) and Streptomyces reniochalinae LHW50302^T^ (98.32%), which are marine sponge-derived *Streptomyces* (24). In the maximum likelihood phylogenetic tree (Fig. 1A), *S. tubbatahanensis* DSD3025^T^ formed a monophyletic lineage with *S. diacarni* LHW51701^T^, and their nearest neighbor was *S. reniochalinae* LHW50302^T^. This topology was consistent with that observed in phylogenetic trees analyzed using neighbor-joining and maximum parsimony algorithms (see Fig. S1 in the supplemental material). In contrast, the multilocus sequence analysis (MLSA) of concatenated housekeeping gene sequences revealed that *S. tubbatahanensis* DSD3025^T^ formed a distinct lineage from its closely related *Streptomyces* species in the phylogenetic trees based on the three algorithms (Fig. 1B; Fig. S2).

**FIG 2:**
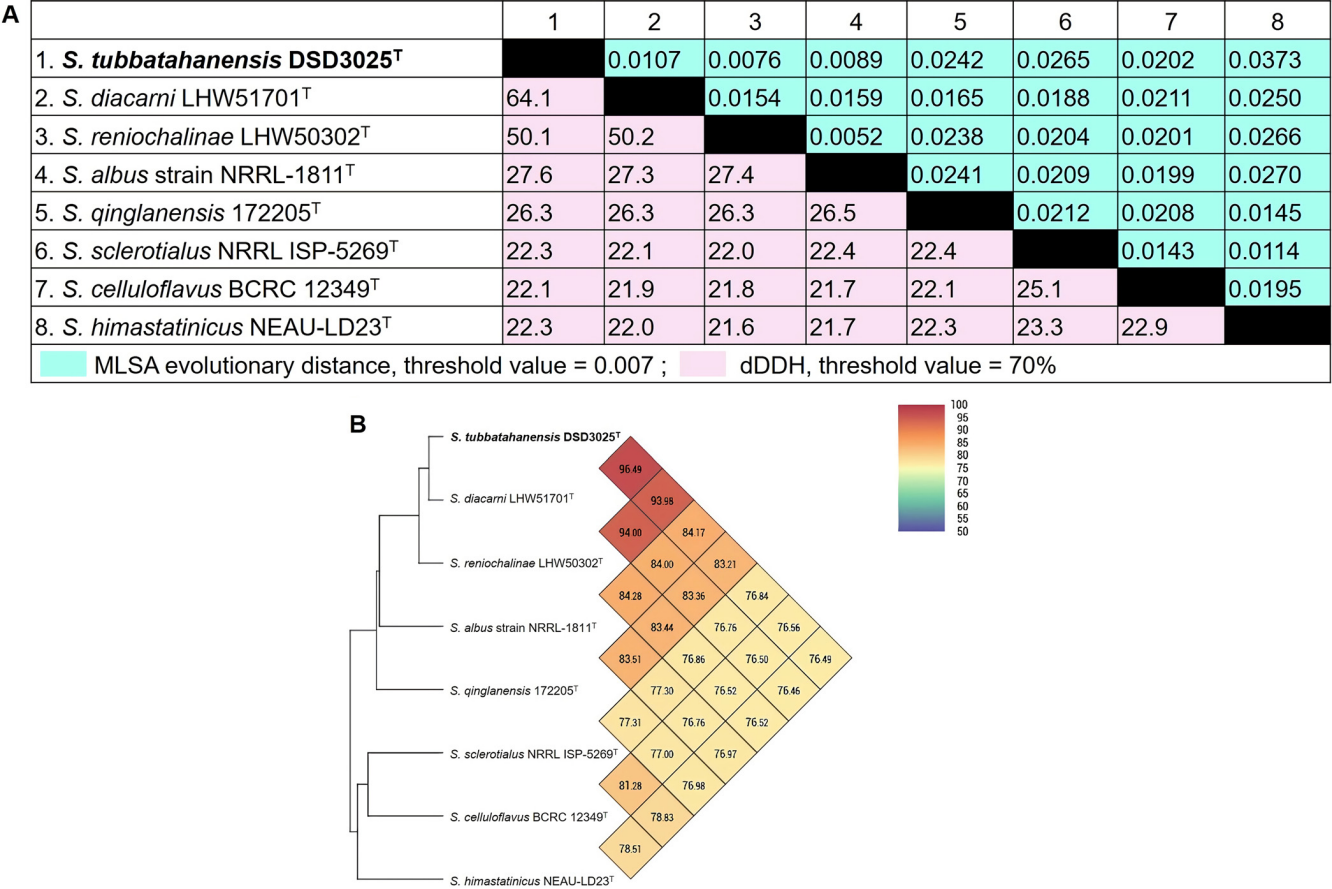
Taxonomic tools for the identification of novel *Streptomyces* species. (A) MLSA evolutionary distance and dDDH comparison between *S. tubbatahanensis* DSD3025^T^ and phylogenetic neighbors. (B) orthoANI v0.93.1 analysis using OAT software for *S. tubbatahanensis* DSD3025^T^ and related *Streptomyces* species.

Based on MLSA, the evolutionary distance between *S. tubbatahanensis* DSD3025^T^ and its closely related *Streptomyces* species was 0.0107 (Fig. 2A), which was above the cutoff value of 0.007 to delineate novel species under the genus *Streptomyces* (25). To further validate the species delineation of *S. tubbatahanensis* DSD3025^T^, the digital DNA-DNA hybridization (dDDH) and orthologous average nucleotide identity (orthoANI) values were calculated to validate the taxonomic status as a novel species. The dDDH values between *S. tubbatahanensis* DSD3025^T^ and its closely related matches ranged from 22.1 to 64.1% (Fig. 2A), which were below the threshold value of 70% (26). Notably, *S. tubbatahanensis* DSD3025^T^ and its nearest species neighbor, *S. diacarni* LHW51701^T^, had a dDDH value of 64.1%. In addition, *S. tubbatahanensis* DSD3025^T^ showed an orthoANI value of 96.49% with *S. diacarni* LHW51701^T^ (Fig. 2B). In a recent study, a 70% dDDH corresponded to approximately 96.7% and not the 95 to 96% cutoff point in delineating *Streptomyces* species (27). These findings suggested that *S. tubbatahanensis* DSD3025^T^ is a novel species based on the MLSA, dDDH, and orthoANI values between *S. tubbatahanensis* DSD3025^T^ and its closest match, *S. diacarni* LHW51701^T^. The taxonomic status of *S. tubbatahanensis* DSD3025^T^ as a novel strain was further validated using phenotypic, chemotaxonomic, and genomic analyses.

### Genome annotation and analysis.

**FIG 3:**
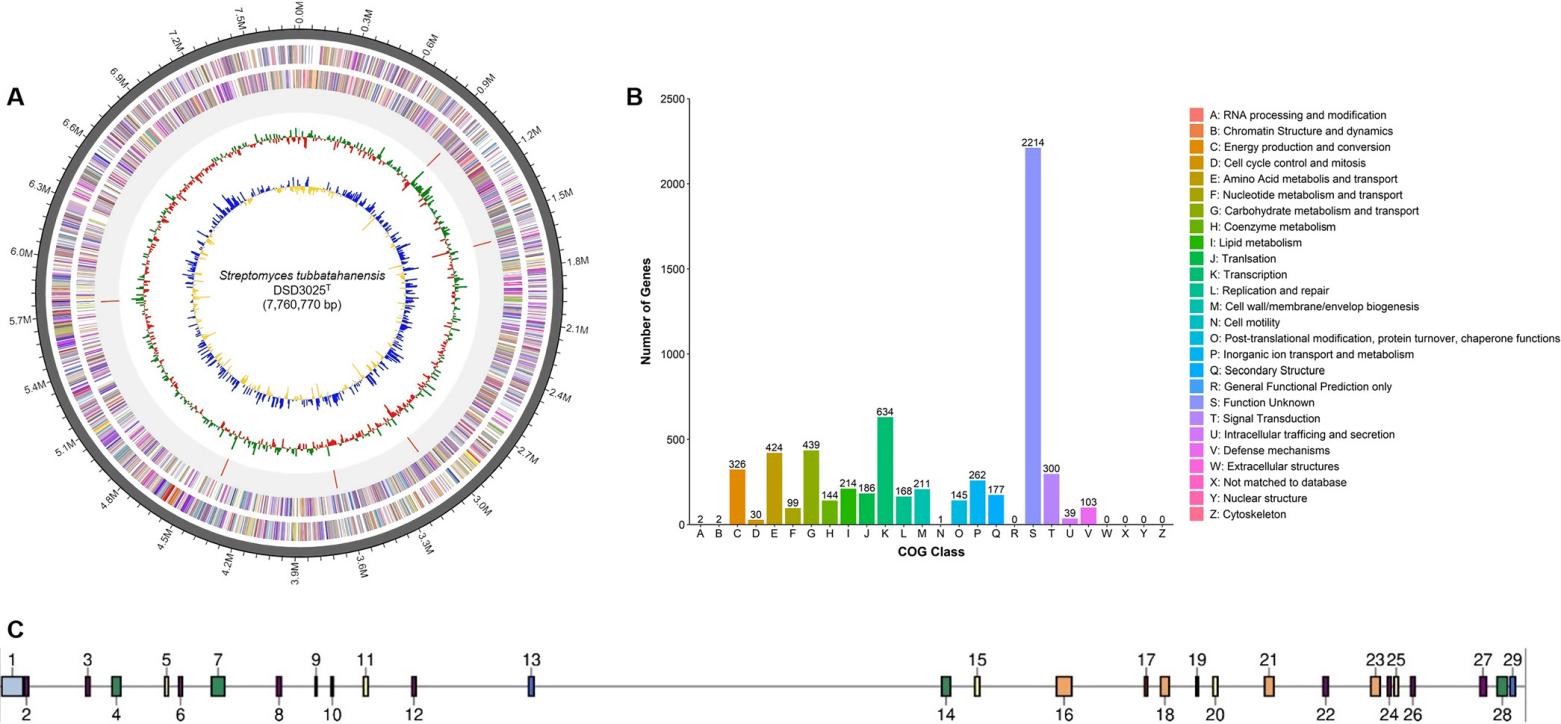
Genome annotations of *S. tubbatahanensis* DSD3025^T^. (A) The circular map of the *S. tubbatahanensis* DSD3025^T^ genome, displaying contig information in the outer circle, while the next adjacent circles show the distribution of COG genes in the forward and reverse strands, respectively. The next circle displays the rRNA and tRNA, followed by a circle for GC skew, with higher-than-average values displayed in green and lower-than-average values displayed in red. The next inner circle displays the GC ratios, with higher-than-average values in blue and lower-than-average values in yellow. (B) Clusters of Orthologous Groups (COGs) functional features. (C) Visualized locations of the 29 specialized metabolite BGC regions in the linearized genome of *S. tubbatahanensis* DSD3025^T^, as predicted by antiSMASH v.7.0. BGC regions ranged from 7,831 to 106,511 nucleotides.

The whole genome of *S. tubbatahanensis* DSD3025^T^, consisting of 7,760,770 bp and with a 72.3% G+C content (Table 1), was deposited in the GenBank database under accession number CP093846. The chromosome of *S. tubbatahanensis* DSD3025^T^ was assembled as a single large contig (Fig. 3A). It contains 6,579 genes further divided into 4,313 operons. In the genome of *S. tubbatahanensis* DSD3025^T^, 6,567 genes were counted as protein coding sequences (CDS), 70 as RNA genes, 6 as rRNA genes, and 69 as tRNA genes. Functional analysis by clusters of orthologous genes (COGs) revealed that 6,120 were assigned COG categories. Most of the predicted COGs had unknown functions (2,214), as shown in Fig. 3B. The other remaining 3,906 COG functional genes were largely assigned to transcription (634), carbohydrate transport and metabolism (439), and amino acid transport and metabolism (424). To some extent, the COGs with unknown functions required further classification and validation to determine their function.

**TABLE 1:** Genome features of *S. tubbatahanensis* DSD3025^T^

Feature	Value	Feature	Value
Chromosome size (bp)	7,760,770	Genes for amino acid and derivative metabolism	396
GC content (%)	72.3	Genes for carbohydrate metabolism	338
Contigs	1	Genes for protein metabolism	250
Operons	4,313	Core genes	392
RNA genes	70	Genomic islands	11
rRNA genes	6	Transposases	15
tRNA genes	69	Integrases	5
Genes assigned to COG	6,120	Known resistance model hits	40
Predicted genes	6,579	Drug resistance class	13
CDS	6,567	Drug resistance mechanisms	6
Subsystem features	320	Secondary metabolite gene clusters	29

Eleven genomic islands were identified in the strain’s genome (Table S1), linking specialized metabolism to functional adaptation (28). Mobility genes, such as transposases and integrases, were detected (Table S2) and were associated with the movement of mobile genetic elements. Based on RAST annotation, 320 subsystems were identified where the majority of the genes were linked to amino acid and derivative metabolism (18.8%) and carbohydrate metabolism (16.1%), followed by protein metabolism (11.9%) (Fig. S3). ARTS predicted 392 core genes and 40 known resistance model hits in the *S. tubbatahanensis* DSD3025^T^ genome (Table S3). The *in silico* resistome analysis of *S. tubbatahanensis* DSD3025^T^ genome revealed drug resistance against 13 drug classes with six resistance mechanisms (Table S4), which was similar to closely related *Streptomyces* species. The resistance genes encoded in the *S. tubbatahanensis* DSD3025^T^ genome unveiled self-resistance against different antibiotic compounds, an important feature of antibiotic-producing strains to avoid suicide (29). These predicted resistance genes in DSD3025^T^ were not tested *in vitro* in this study. A total of 29 biosynthetic gene clusters associated with specialized metabolite production were predicted in the strain’s genome (Fig. 3C), indicating the richness of the biosynthetic gene clusters, which are further discussed below.

### Phenotypic analysis.

**FIG 4:**
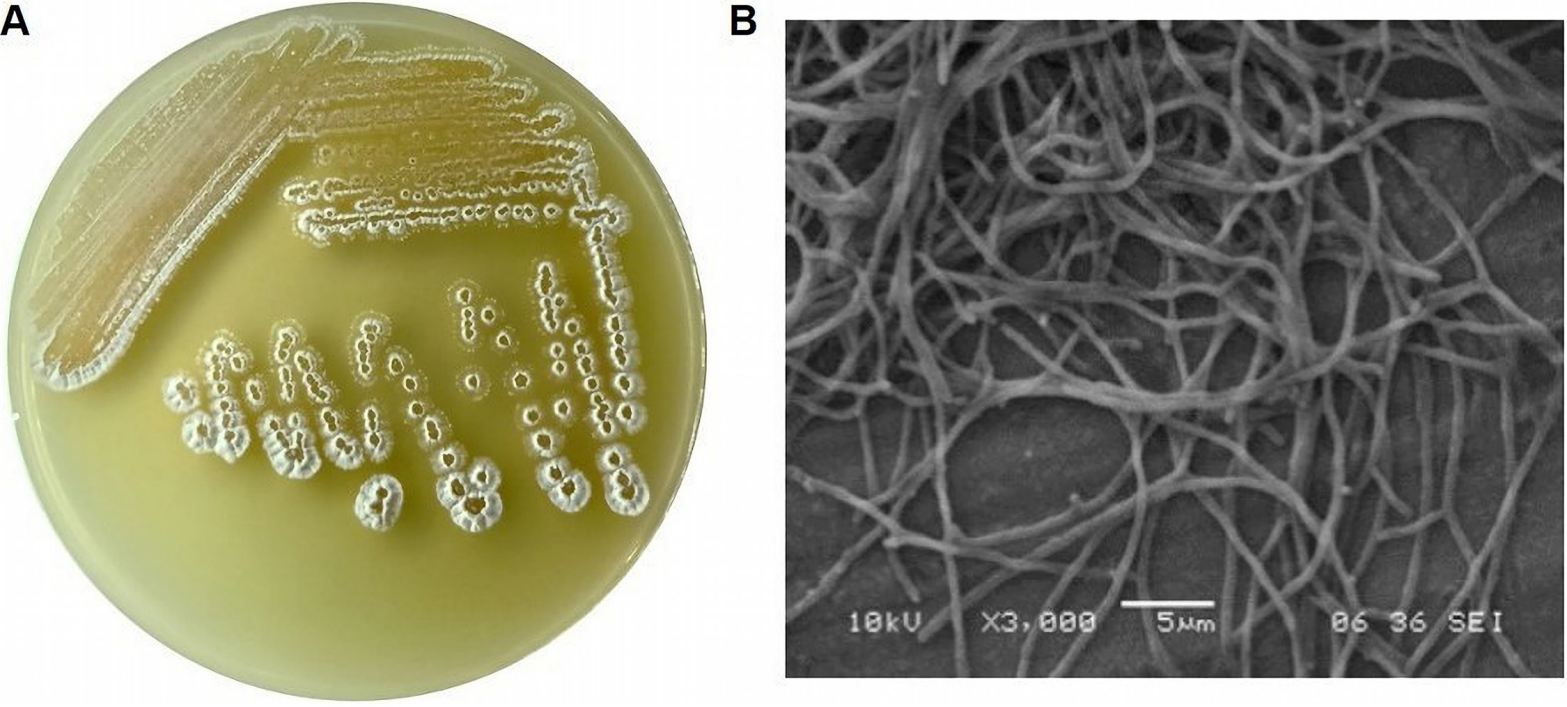
Cultural morphology of *S. tubbatahanensis* DSD3025^T^ on MM1 agar (A) and a scanning electron micrograph showing mycelial filaments and intertwining hyphal cells (B).

Colonies on marine medium 1 (MM1) agar at 14 days of incubation formed well-developed aerial and substrate mycelium, which are the phenotypic features of *Streptomyces*. Spores were not evident in the scanning electron microscopy (SEM) image, but the intertwining of hyphal cells was observed (Fig. 4). Growth of *S. tubbatahanensis* DSD3025^T^ was abundant on MM1 agar, yeast extract-glucose-calcium carbonate (YGC) medium, nutrient agar (NA), tryptic soy agar (TSA), International Streptomyces Project 2 (ISP2) and ISP3 media, with white aerial mycelium and light to deep orange-yellow substratum mycelium (Table S5). *S. tubbatahanensis* DSD3025^T^ had moderate growth in MM11 and ISP9. Poor growth was observed in mannitol-containing agar (MM3) and ISP4. No diffusible pigment was produced by the strain in the different culture media. The API ZYM is a system used to detect selected enzymes in *S. tubbatahanensis* DSD3025^T^. After 4 h of incubation, the results were assessed based on the standard reading table of the API ZYM system (Fig. S4). Of 19 enzymatic reactions, *S. tubbatahanensis* DSD3025^T^ showed seven positive enzymatic activities for alkaline phosphate, esterase (C_4_), leucine arylamidase, acid phosphatase, and naphthol phosphohydrolase and weakly positive activities for esterase and lipase (C_8_) and α-glucosidase enzymes (Table S6). The enzymatic activities expressed by different species of *Streptomyces* vary and can therefore be used to differentiate *Streptomyces* species from their closely related matches.

*S. tubbatahanensis* DSD3025^T^ had observed growth in marine medium formulated with 0 to 15% NaCl. Growth was observed at 28°C and 37°C and over a pH range of 4.0 to 10.0 (Table 2). The closely related *Streptomyces* matches of *S. tubbatahanensis* DSD3025^T^ had similar observed growth in the 0 to 15% NaCl range and at 28°C and 37°C. The NaCl tolerance test demonstrated that *S. tubbatahanensis* DSD3025^T^ was not an obligate marine *Streptomyces* but had an adaptive capacity to grow in up to 15% (wt/vol) NaCl. Notably, *S. tubbatahanensis* DSD3025^T^ showed abundant growth at pH 4.0, in contrast with its neighboring *Streptomyces* species.

**TABLE 2:** Phenotypic characteristics of *S. tubbatahanensis* DSD3025^T^ and its closely related species

Characteristic	*Streptomyces tubbatahanensis* DSD3025^T^	*S. reniochalinae* LHW50302^T^ (24)	*S. diacarni* LHW51701^T^ (24)
Morphology on ISP2			
Aerial mycelium	White	Pale to grayish greenish yellow	Yellowish white
Substratum mycelium	Pale orange yellow	Dark orange yellow	Pale orange yellow
Diffusible pigment	−	−	−
Growth at:			
4°C	−	−	−
28°C	+	+	+
37°C	+	+	+
pH 4.0	+	−	−
pH 7.0	+	+	+
pH 10.0	+	+	+
NaCl (0%)	+	+	+
NaCl (7%)	+	+	+
NaCl (10%)	+	+	+
NaCl (15%)	+	+	+

### Chemotaxonomic analysis.

The predominant respiratory quinone of *S. tubbatahanensis* DSD3025^T^ was MK-9 (H8) (44.9%) followed by MK-9 (H6) (32.5%) and MK-9 (H4) (11.9%) as shown in the summary of chemotaxonomic profile in Table 3. The MK-9 (H2) (6.3%) and MK-9 (4.3%) were the minor menaquinones of *S. tubbatahanensis* DSD3025^T^ present in trace amounts that were not present in the menaquinone system of neighboring *Streptomyces* species. The major cellular fatty acids (>10%) of *S. tubbatahanensis* DSD3025^T^ were C_16:0_ (36.15%) and C_18:1ω9c_ (13.86%). A major difference in the fatty acid profile of *S. tubbatahanensis* DSD3025^T^ that was not detected in its closely related *Streptomyces* species was the summed feature 5 (C_18:0ANTE_/C_18:2ω6,9c_), comprising 45.09%. Interestingly, the majority of the polar lipids described were unidentified phospholipids (PL), followed by phosphatidylethanolamine, aminophospholipids, diphosphatidylglycerol, glycolipids, and unidentified lipids (Fig. S5). The whole-cell sugars were glucose and xylose. The chemotaxonomic characteristics of *S. tubbatahanensis* DSD3025^T^ were typical features of members under the genus *Streptomyces* (30), with major chemotaxonomic differences observed between *S. tubbatahanensis* DSD3025^T^ and its close *Streptomyces* relatives. Collectively, the genomic, phenotypic, and chemotaxonomic features highlighted *S. tubbatahanensis* DSD3025^T^ as a novel species.

**TABLE 3:** Chemotaxonomic profile of *S. tubbatahanensis* DSD3025^T^ and closely related species

Chemotaxonomic characteristic	*S. tubbatahanensis* DSD3025^T^	*S. reniochalinae* LHW50302^T^ (24)	*S. diacarni* LHW51701^T^ (24)
Major menaquinones (%)			
MK-9	4.3	—	—
MK-9 (H2)	6.3	—	—
MK-9 (H4)	11.9	11.6	13.9
MK-9 (H6)	32.5	76.0	74.3
MK-9 (H8)	44.9	6.4	4.8
Major fatty acids (%)			
14:0	0.17	0.2	—
15:0 anteiso	1.14	22.7	21.5
16:0	36.15	2.4	2.3
16:0 iso	0.61	28.4	27.2
17:0 anteiso	0.50	19.3	21.4
18:0	2.13	0.2	—
18:1 ω9c	13.86	—	—
Summed feature 3^*a*^	0.35	0.2	0.4
Summed feature 5^*b*^	45.09	—	—
Summed feature 8^*c*^	—	—	—
Summed feature 9^*d*^	—	0.9	1.5
Major polar lipids^*e*^	DPG, PE, APL, GL, PL, L	DPG, PE, PI	DPG, PE, PI
Whole-cell sugars	Glucose, xylose	Glucose, galactose, ribose	Glucose, galactose, ribose

a Summed feature 3: for **1**, 16:1 ω7c/15 iso 2OH; for compounds **2** and **3**, 16:1 ω7c/16:1 ω6c.

b Summed feature 5: 18:0 ANTE/C18:2 ω6,9c.

c Summed feature 8: 18:1 ω7c.

d Summed feature 9: 17:1 iso ω9c.

e DPG, diphosphatidylglycerol; PE, phosphatidylethanolamine; APL, aminophospholipids; GL, glycolipids; PL, unidentified phospholipids; L, unidentified lipids.

### *In silico* specialized metabolite analysis of BGC in *S. tubbatahanensis* DSD3025^T^.

The 29 clusters predicted by antiSMASH (v.7.0) were associated with the production of BGCs consisting of nonribosomal peptides, indole biosynthesis, siderophore, type-1 (T1PKS) and type-2 (T2PKS) polyketides, posttranslationally modified peptides (RiPP-like), thiopeptides, and lanthipeptides (Table 4). BGCs 9, 10, and 21 have 100% similarity with known BGCs encoding desferrioxamine E, ectoine, and marineosin A and B, respectively. The remaining clusters with <70% similarity to known specialized metabolites were identified as NRPS, indole, terpene, T1PKS, T2PKS, siderophore, lanthipeptides, betalactone, and thiopeptides. Notably, BGC regions 2, 3, 11, 15, 18, and 27 had no similarity with the reference specialized metabolites, indicating the novelty of the BGCs in *S. tubbatahanensis* DSD3025^T^. Since *S. diacarni* LHW51701^T^ was the nearest neighbor in the phylogenetic analysis, we compared its BGCs to *S. tubbatahanensis* DSD3025^T^. We noted similar BGCs encoded between two species for the biosynthesis of marineosin A and B, desferrioxamine E, ectoine, reductasporine, SapB, ebelactone, and hopene (31). We explored the biosynthetic potential of *S. tubbatahanensis* DSD3025^T^ by examining the putative core structures of the compounds produced by the NRPS and some PKS BGCs (using PRISM4) (Fig. S6), as well as the predicted core peptides of the RiPPs (detected using BAGEL4) (Fig. S7). These analyses highlighted the structurally diverse biosynthetic potential of *S. tubbatahanensis* DSD3025^T^.

**TABLE 4:** BGCs in *S. tubbatahanensis* DSD3025^T^ predicted by AntiSMASH v.7.0

Cluster	Type	Location in *S. tubbatahanensis* DSD3025^T^ genome (nt)	Specialized metabolite encoded by a predicted BGC	Similarity
1	NRPS, betalactone	13,600–120,111	Gausemycin A, gausemycin B	50%
2	Terpene	128,097–147,797	NA^*a*^	
3	Terpene	439,716–460,624	NA	
4	NRPS	573,845–617,336	Griseobactin	69%
5	Indole	840,693–859,694	Reductasporine	66%
6	Terpene	910,976–930,923	Petrichorin A, petrichorin B	11%
7	NRPS	1,080,242–1,145,604	Thiazostatin, watasemycin A, watasemycin B, 2-hydroxyphenylthiazoline enantiopyochelin, isopyochelin	46%
8	Terpene	1,410,232–1,433,798	Isorenieratene	85%
9	Siderophore	1,606,362–1,614,193	Desferrioxamine E	100%
10	Ectoine	1,687,437–1,697,841	Ectoine	100%
11	Lanthipeptide class I	1,852,259–1,874,688	NA	
12	T2PKS	2,098,499–2,118,273	Hygromycin A	6%
13	Thiopeptide, LAP	2,691,345–2,718,846	NA	
14	NRPS	4,791,840–4,834,676	Dudomycin A	52%
15	Lanthipeptide class I	4,960,208–4,985,084	NA	
16	T2PKS	5,375,496–5,452,293	Tetrocarcin A	4%
17	RiPP-like	5,823,990–5,839,349	Granaticin	8%
18	Siderophore	5,904,616–5,949,360	NA	
19	T1PKS	6,084,971–6,096,168	Neoabyssomicin, abyssomicin	6%
20	Lanthipeptide class IV	6,171,917–6,194,700	Meilingmycin	3%
21	T1PKS, prodigiosin	6,433,729–6,479,833	Marineosin A, marineosin B	100%
22	Terpene	6,731,707–6,756,420	Hopene	61%
23	T1PKS	6,974,617–7,020,874	SapB	50%
24	Terpene	7,057,983–7,077,896	Hygrocin A, hygrocin B	6%
25	Indole	7,091,656–7,113,017	Anisomycin	38%
26	Terpene	7,177,792–7,199,270	Ebelactone	5%
27	Betalactone	7,528,873–7,561,275	NA	
28	NRPS	7,617,134–7,667,960	Arylomycin	22%
29	Thiopeptide, LAP	7,681,670–7,709,509	A54145	5%

a NA, not available.

Based on BGC analysis and comparison to known databases, the BGC region 1 of *S. tubbatahanensis* DSD3025^T^ resembled the BGC architecture of recently discovered cyclic halogenated lipoglycopeptide gausemycins A and B from *Streptomyces* (32). The unique structural features of the gausemycins A and B gene clusters were found in *S. tubbatahanensis* DSD3025^T^ BGC region 1, including the core *gauA*, *gauB*, *gauC*, and *gauD* NRPS gene homologs, as well as the incorporation of 4-chlorokynurenine (4-Cl-Kyn) (33) and 2-amino-4-hydroxy-4-phenylbutyric acid. The halogenation and conversion of tryptophan to 4-Cl-Kyn were facilitated by a tryptophan 6-halogenase (*tar14*) (33), flavin reductase, tryptophan-2,3-dioxygenase, and a putative α/β-hydrolase, for which the homologs were found in BGC region 1 of *S. tubbatahanensis* DSD3025^T^.

**FIG 5:**
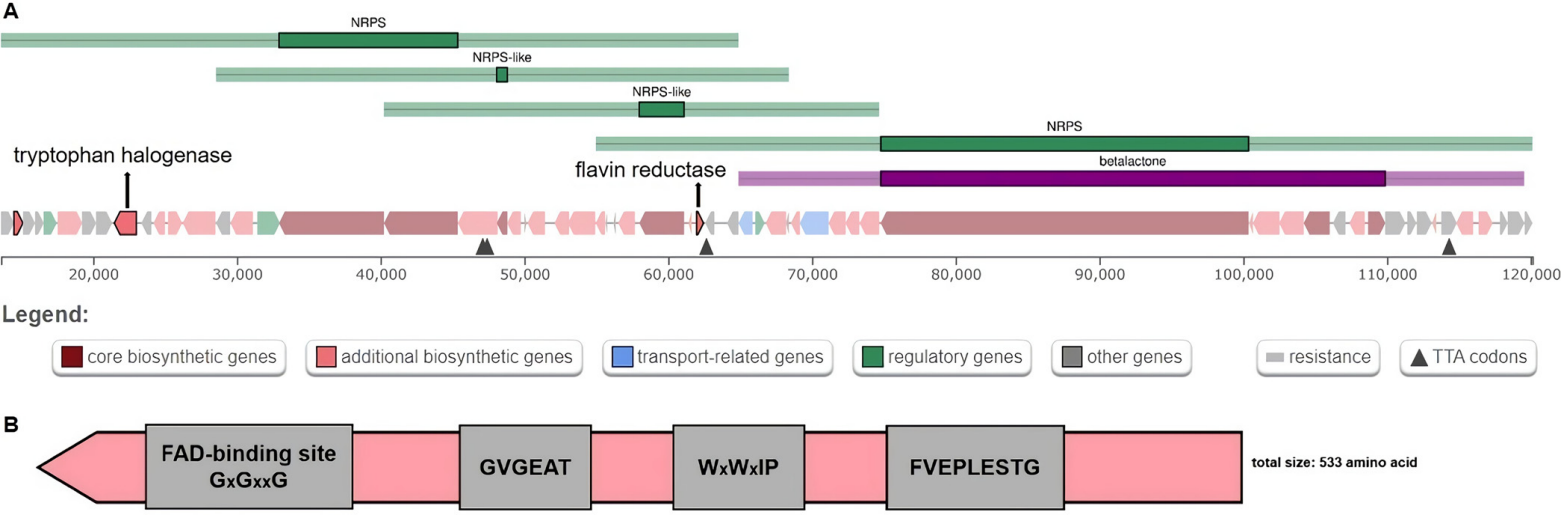
Biosynthetic gene cluster containing the halogenase gene and its conserved motifs encoded in the *S. tubbatahanensis* DSD3025^T^ genome. (A) BGC region 1 in *S. tubbatahanensis* DSD3025^T^ harbored the two-component halogenase/reductase system, the tryptophan halogenase, and its associated flavin reductase. (B) Amino acid sequence assembly of tryptophan halogenase in *S. tubbatahanensis* DSD3025^T^, showing the conserved motifs for FADH_2_-dependent halogenases.

Interestingly, an FADH_2_-dependent tryptophan halogenase and an associated flavin reductase were identified in BGC region 1 of *S. tubbatahanensis* DSD3025^T^ (Fig. 5A). This two-component halogenase/reductase system was not detected in *S. diacarni* LHW51701^T^, thus indicating differences in the biosynthesis of halogenated compounds between the two related strains. The identified tryptophan halogenase homolog had an approximate size of 533 amino acids (Fig. 5B) that may install chlorine or bromine on tryptophan substrates with high regioselectivity and specificity.

The conserved motifs of this FADH_2_-dependent halogenase (FDH) included a putative flavin-binding motif (GxGxxG) located at the amino-terminal end (34), a C-terminal motif (WxWxIP) found in the middle of the sequence (34) that prevents monooxygenase substrate binding near the flavin (35, 36), and other conserved motifs identified in the halogenase genes of motifs such as GVGEAT and FVEPLESTG (37, 38). The lysine and glutamate catalytic residues highly conserved in FDHs were also observed in the tryptophan halogenase homolog sequence of *S. tubbatahanensis* DSD3025^T^. The phylogenetic analysis revealed that the tryptophan halogenase of *S. tubbatahanensis* DSD3025^T^ formed a separate clade, together with *tar14* and other bacterial C-6 halogenases (*sttH*, *ktzR*, and th-Hal) (39–41) (Fig. S8), suggesting that the tryptophan halogenase of *S. tubbatahanensis* DSD3025^T^ was a tryptophan FDH that may catalyze site-selective C-6 halogenation of specialized metabolites instead of the C-7 halogenation annotated from the whole-genome sequence analysis. The flavin reductase found in the same gene cluster was essential for the enzymatic cofactor regeneration of FADH_2_ needed by the tryptophan halogenase to function (42).

### Metabolite profiling of *S. tubbatahanensis* DSD3025^T^ extract revealed halogenated compounds.

To gain insights into the specialized metabolites biosynthesized by *S. tubbatahanensis* DSD3025^T^, the crude extract was analyzed using ultra performance liquid chromatography–electrospray ionization–quadrupole time-of-flight mass spectrometry (UPLC-ESI-QTOF-MS). At least six mass ion peaks of *S. tubbatahanensis* DSD3025^T^ extract in the negative mode showed a 3:1 isotopic pattern of chlorine and a 1:1 isotopic pattern of bromine (Fig. S9 and S10), suggesting that *S. tubbatahanensis* DSD3025^T^ produces halogenated specialized metabolites.

**FIG 6:**
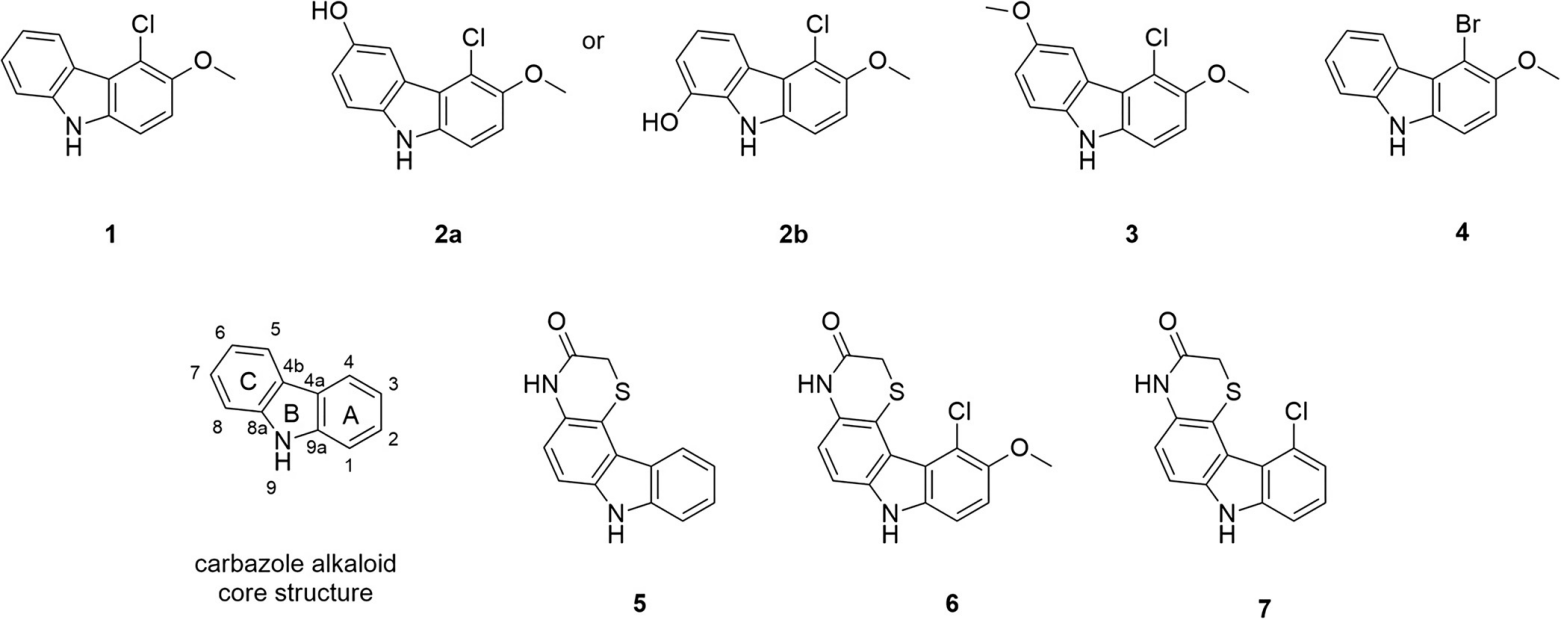
Structures of chlorinated carbazole alkaloids, namely, chlocarbazomycin A (**1**), chlocarbazomycin B (**2a**) or C (**2b**), chlocarbazomycin E (**3**), brocarbazomycin A (**4**), thiocarbazomycins A (**5**) and B (**6**), and a new compound (**7**) from *Streptomyces tubbatahanensis* DSD3025^T^.

The mass ion peaks detected at *m/z* 230.0376 [M − H]^−^ (C_13_H_9_NOCl; DBE = 9.5, mass error = 1.3 ppm, 100% fit confidence, *t_R_* 5.24 min) and *m/z* 246.0324 [M − H]^−^ (C_13_H_9_NO_2_Cl; DBE = 9.5, mass error = 0.8 ppm, 100% fit confidence, *t_R_* 4.20 min) were identified as known antimicrobial chlorinated carbazole alkaloids **1** and **2a** or **2b**, based on MS and tandem MS (MS/MS) analyses (Fig. 6) (Fig. S11 to S13). The identity of the compound detected at *m/z* 246.0324 [M − H]^−^ could not be unambiguously deduced as **2a** or **2b** by MS/MS due to positional symmetry.

The metabolite profiling of *S. tubbatahanensis* DSD3025^T^ revealed the presence of mass ion peaks that matched the recently reported novel molecules thiocarbazomycins A and B, chlocarbazomycin E, and brocarbazomycin A, isolated from coral and coral sands-derived *S. diacarni* SCSIO 64983^T^ (43). These mass ion peaks were detected at *m/z* 260.0490 [M − H]^−^ (chlocarbazomycin E **3**: C_14_H_11_NO_2_Cl; DBE = 9.5, mass error = 4.6 ppm, 90.93% fit confidence, *t_R_* 4.98 min); *m/z* 273.9881 [M − H]^−^ (brocarbazomycin A **4**: C_13_H_9_NOBr; DBE = 9.5, mass error = 4.7 ppm, 100% fit confidence, *t_R_* 5.39 min); *m/z* 253.0441 [M − H]^−^ (thiocarbazomycin A **5**: C_14_H_9_N_2_OS; DBE = 11.5, mass error = 2.0 ppm, 100% fit confidence, *t_R_* 3.38 min); and *m/z* 317.0164 [M − H]^−^ (thiocarbazomycin B **6**: C_15_H_10_N_2_O_2_SCl; DBE = 11.5, mass error = 3.8 ppm, 99.90% fit confidence, *t_R_* 3.24 min), respectively (Fig. 6 and Fig. S14 to S17). Remarkably, 6 out of 7 compounds isolated from *S. diacarni* SCSIO 64983^T^, including **1**, **2a**, and **2b**, were found in *S. tubbatahanensis* DSD3025^T^ extract in the negative mode (Table S7). The metabolite similarities observed between *S. tubbatahanensis* DSD3025^T^ and the recently reported *S. diacarni* SCSIO 64983^T^ indicated that these *Streptomyces* species belong to one major clade (96% bootstrap replicates), based on a reconstructed phylogenetic tree (Fig. S18) using the 16S rRNA gene sequences of other *S. diacarni* strains and closely related *Streptomyces* species. Moreover, *S. tubbatahanensis* DSD3025^T^ formed a subclade branch with *S. diacarni* SCSIO 68036 and SCSIO 68034, as supported by 67% bootstrap replicates.

The molecular networking analyses via MS-DIAL (Fig. S19) revealed another rare chlorinated sulfur-containing carbazole moiety at *m/z* 287.0054 [M − H]^−^: compound 7 (C_14_H_8_N_2_OSCl; DBE = 11.5, mass error = 2.8 ppm, 99.92% fit confidence, *t_R_* 4.20 min) (Fig. S20) from *S. tubbatahanensis* DSD3025^T^ that clustered with chlocarbazomycin A (56.8% similarity) not found in closely related *S. diacarni* strains. The extensive search of its neutral chemical formula showed no hits in MarinLit, ChemSpider, PubChem, or AntiBase, suggesting that the compound is a possible new specialized metabolite with halogenation located at C-4 of carbazole ring A.

In the MS positive mode, five mass ion peaks with chlorine isotopic patterns were identified from *S. tubbatahanensis* DSD3025^T^ extract (Table S8 and Fig. S21 to S25). The mass ion peak at *m/z* 232.0521 [M + H]^+^ (C_13_H_11_NOCl; DBE = 8.5, mass error = −3.4 ppm, 94.80% fit confidence, *t_R_* 5.24 min) was recognized as **1** based on retention time, MS, and MS/MS, while two later-eluting compounds with the same mass (*m/z* 517.1436 [M + H]^+^) (C_31_H_22_N_4_O_2_Cl; DBE = 22.5, mass error = 1.0 ppm, 99.97% fit confidence, *t_R_* 7.23 min) *m/z* 517.1439 [M + H]^+^ (C_31_H_22_N_4_O_2_Cl; DBE = 22.5, mass error = 1.5 ppm, 99.96% fit confidence, *t_R_* 8.61 min), chemical formula, and MS/MS but different retention time were identified as structural isomers. The exhaustive search using the chemical formulas of *m/z* 317.1419 [M + H]^+^ (C_18_H_22_N_2_OCl; DBE = 8.5, mass error = −0.6 ppm, 100% fit confidence, *t_R_* 6.68 min), *m/z* 457.0958 [M + H]^+^ (C_26_H_18_N_2_O_4_Cl; DBE = 18.5, mass error = 0.7 ppm, 99.82% fit confidence, *t_R_* 7.05 min), and structural isomers *m/z* 517.1436 [M + H]^+^ and *m/z* 517.1439 [M + H]^+^ showed no hits for known microbial natural products in MarinLit, ChemSpider, or AntiBase, suggesting new chlorinated specialized metabolites. Additionally, the MS/MS results for these unknown chlorinated compounds showed distinct fragment peaks (Fig. S26 to S29), which indicated that they are structurally unique. However, elucidating their structure ID to confirm structural uniqueness requires validation via NMR analyses.

### Isolation and structure elucidation of halogenated carbazole alkaloid.

Given that *S. tubbatahanensis* DSD3025^T^ produced halogenated metabolites based on MS analysis, it was envisaged that a flavin-dependent tryptophan 6-halogenase catalyzed the halogenation of these specialized metabolites (Fig. 6). Carbazole alkaloids utilize tryptophan as a precursor in their biosynthesis (44). The indole nucleus in tryptophan contributes to rings B and C during carbazole biosynthesis; thus, the detected tryptophan 6-halogenase encoded in the *S. tubbatahanensis* DSD3025^T^ genome may facilitate the halogenation of carbazole alkaloids at the C-7 of ring C (Fig. 6). To test this hypothesis, a mass-directed purification of *Streptomyces tubbatahanensis* DSD3025^T^ crude extract was performed.

The halometabolite detected at *m/z* 232.0521 [M + H]^+^ putatively identified as compound **1** was set as the target mass for isolation due to its relatively higher abundance compared to other halometabolites in *S. tubbatahanensis* DSD3025^T^ extract. The purification of 1 g *S. tubbatahanensis* DSD3025^T^ extract (Fig. S30) afforded 4.73 mg (0.5% yield) of DSD3025H1, which was later confirmed as compound **1** via high-resolution MS (HRMS), MS/MS, and NMR analyses (Fig. S31 and S32). The purification of **1** yielded 80% purity as estimated via ^1^H NMR. Notably, *Streptomyces tubbatahanensis* DSD3025^T^ is a relatively more prolific producer of halometabolites, producing 7 times more than its related strain, *Streptomyces diacarni* LHW51701^T^ (45).

Compound **1** was unambiguously identified by NMR spectroscopic data (Table S9) based on ^1^H, ^1^H-decoupled, ^13^C, ^13^C DEPTQ135, ^1^H-^1^H correlation spectroscopy (COSY), heteronuclear single quantum coherence (HSQC), heteronuclear multiple bond correlation (HMBC), and nuclear Overhauser effect spectroscopy (NOESY) NMR spectral analyses (Fig. S33 to S48). The purity of the sample was approximately 80%, as determined by dividing the integral value of the −OCH_3_ peak of the compound by the total integral value of the −OCH_3_ peak of the compound and methyl peak of fatty acid impurities in the aliphatic region (2.0 to 2.15 ppm) of the ^1^H NMR spectrum (Fig. S34). Data from *J* value, COSY, and NOESY were used to determine the position of chlorine in the elucidated compound. NOESY data showed correlations between −NH and H1 (Fig. S47), indicating that H1 was in proximity with −NH and that chlorine was positioned in C-4 of carbazole ring A. In addition, NOESY data demonstrated a correlation between H2 and −OCH_3_, suggesting that these protons were also in proximity and chlorine was adjacent to the −OCH_3_ of **1**. These NOESY correlations indicated that the halogenation occurred at C-4 of carbazole ring A, indicating that compound **1** isolated from *S. tubbatahanensis* DSD3025^T^ has the structure of chlocarbazomycin A, as reported previously from its closely related *Streptomyces* strain, *S. diacarni* LHW51701^T^ (43, 45). These NOESY correlations were verified by measuring the distances between NH and H1 (2.8 Å) and between H2 and −OCH3 (1.9 Å) using Chemdraw 3D v21.0.0 (Fig. S48). The correlation between H1 and H2 identified in the COSY spectrum (Fig. S41) and the calculated *J* value of 8.7 Hz from the ^1^H NMR spectrum showed that H1 and H2 were adjacent to each other (^3^*J*) and that the chlorination occurred at C-4 of carbazole ring A.

### Putative biosynthesis of chlocarbazomycin A compound 1.

Although compound **1** was first isolated just recently from *S. diacarni* strains (43, 45), its biosynthetic pathway was unknown. To elucidate the biosynthetic pathway of **1**, two separate libraries of compounds were created: (i) known halogenated microbial secondary metabolites from tryptophan halogenase precursors, including intermediates in the biosynthesis of rebeccamycin and pyrrolnitrin (Table S10), to deduce whether halogenation occurred before the closure of ring A to form the tricyclic carbazole alkaloid ring; and (ii) nonhalogenated carbazole alkaloids and indolocarbazoles (Table S11), to deduce whether halogenation occurred after tricyclic carbazole ring formation. Mining of the accurate mass of listed compounds and their adducts. [M + H]^+^, [M + Na]^+^, [M + K]^+^, [M + MeCN + H]^+^, and [M − H]^−^, in the mass spectrometry data showed no hits for compounds and intermediates for biosynthesis of rebeccamycin and pyrrolnitrin (Table S10) but showed 3 hits for nonhalogenated carbazole alkaloids and indolocarbazoles (Table S11), namely, carbazomycin B compound **8** and H and carbazoquinocin F (Fig. S49 to S53). Compound **8** was identified based on MS and MS/MS analyses (Fig. S50 and S51), while carbazomycin H and carbazoquinocin F were identified based on the chemical formula, isotope model, and double bond equivalent (Fig. S52 and S53). The absence of intermediates from the biosynthesis of rebeccamycin and pyrrolnitrin, including the chlorinated rebeccamycin analogs, and the detection of nonhalogenated carbazole alkaloids in *S. tubbatahanensis* DSD3025^T^ suggested that the chlorination most likely occurred after the tricyclic carbazole ring formation, i.e., after the closure of ring A, similar to the biosynthesis of tricyclic carbazoles such as neocarazostatins (44).

**FIG 7:**
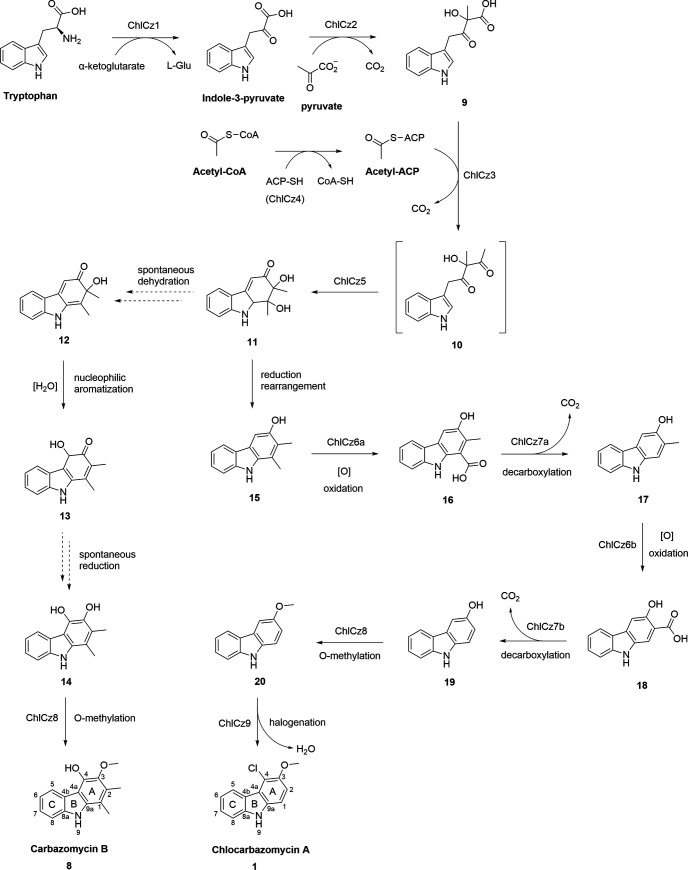
Proposed biosynthetic pathway of chlocarbazomycin A compound **1**.

**FIG 8:**
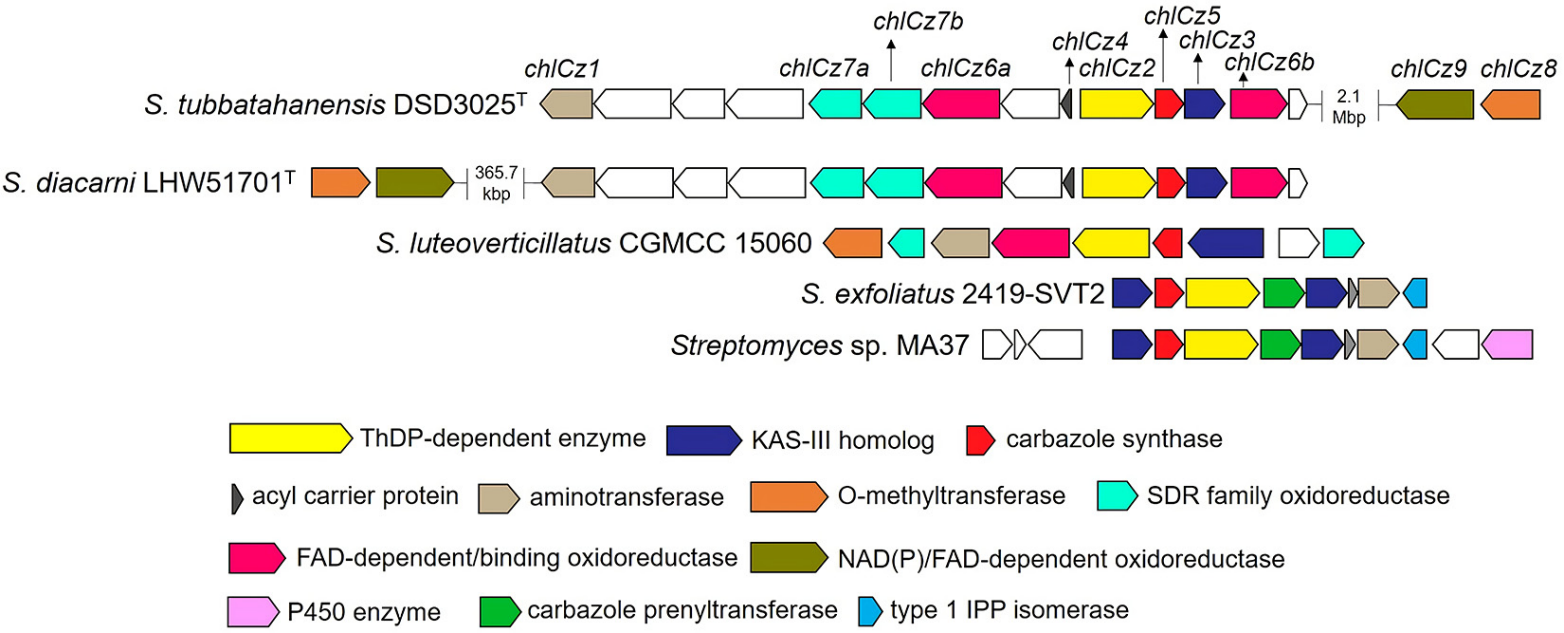
Distribution of biosynthetic gene machineries for carbazole formation in *Streptomyces*. The BGCs for the biosynthesis of neocarazostatin A by *Streptomyces* sp. MA37, carquinostatin A by *S. exfoliatus* 2419-SVT2, carbazomycin B by *S. luteoverticillatus* CGMMC 15060, and chlocarbazomycins by *S. diacarni* LHW51701^T^ and *S. tubbatahanensis* DSD3025^T^ are displayed as key features of carbazole metabolites.

The carbazole alkaloids, such as neocarazostatin A and compound **8**, produced by bacteria have a carbazole nucleus derived from tryptophan, pyruvate, and acetate (46–48). The biosynthetic pathway of **1** proposed in this study (Fig. 7) followed the initial formation of a carbazole nucleus, as observed in the biosynthesis of **8** (49). Comprehensive genome mining and bioinformatics allowed the retrieval of a candidate biosynthetic gene cluster (*chlCz*) in BGC region 16 spanning approximately 22.3 kB, with open reading frames (ORFs) involved in the formation of chlorinated carbazole alkaloids (Fig. 8, Table 5). The genome mining and gene cluster comparison with neocarazostatin A (44) and compound **8** (49) unveiled the key biosynthetic genes clustered in one BGC region of *S. tubbatahanensis* DSD3025^T^ (Table 5) for the formation of a tricyclic carbazole skeleton. The first step of carbazole assembly was the conversion of tryptophan into indole-3-pyruvate catalyzed by a putative aminotransferase *chlCz1* (*nzsD* homolog in NZS biosynthesis) that shared 97.5% homology with histidinol-phosphate transaminase of *S. diacarni* LHW51701^T^. A thiamine pyrophosphate-dependent enzyme, *chlCz2* (*nzsH* homolog in NZS biosynthesis), with 97.3% gene similarity to a thiamine pyrophosphate-binding protein of *S. diacarni* LHW51701^T^ catalyzed the C-C bond between indole-3-pyruvate and pyruvate to form α-hydroxy-β-keto acid **9**.

**TABLE 5:** Deduced functions of genes in the *chlCz* biosynthetic gene cluster

Gene name	Size (bp)	Protein homolog (accession no.), origin	% similarity	Proposed function
*chlCz1*	365	Histidinol-phosphate transaminase (NCBI WP_114024014.1), *Streptomyces diacarni*	97.5	Aminotransferase
*chlCz2*	585	Thiamine pyrophosphate-binding protein (NCBI WP_114024022.1), *Streptomyces diacarni*	97.3	Acetolactate synthase
*chlCz3*	341	3-Oxoacyl-ACP synthase (NCBI WP_114024023.1), *Streptomyces diacarni*	98.8	KAS III
*chlCz4*	83	Acyl carrier protein (NCBI WP_027735527.1), *Streptomyces diacarni*	98.8	ACP
*chlCz5*	199	SRPBCC family protein (NCBI WP_245979595.1), *Streptomyces diacarni*	100	Carbazole synthase
*chlCz6a*	441	FAD-binding oxidoreductase (NCBI WP_114024020.1), *Streptomyces diacarni*	98.4	Oxidase
*chlCz6b*	391	FAD-dependent oxidoreductase (NCBI WP_114024024.1), *Streptomyces diacarni*	99.0	Oxidase
*chlCz7a*	308	SDR family NAD(P)-dependent oxidoreductase (NCBI WP_114024018.1), *Streptomyces diacarni*	97.7	Decarboxylase
*chlCz7b*	338	SDR family oxidoreductase (NCBI WP_114024019.1), *Streptomyces diacarni*	98.8	Decarboxylase
*chlCz8* ^*a*^	365	Methyltransferase (NCBI WP_114021579.1), *Streptomyces diacarni*	93.2	O-methyltransferase
*chlCz9* ^*a*^	569	NAD(P)/FAD-dependent oxidoreductase (NCBI RCG24729.1), *Streptomyces diacarni*	95.1	Flavin-dependent halogenase

a Gene is located outside the biosynthetic gene cluster.

In contrast with the reported biosynthesis of neocarazostatin A that utilized the β-hydroxy group as its acyl substrate, the *chlCz3* (*nzsJ* homolog in NZS biosynthesis) with 98.8% gene similarity to 3-oxoacyl-ACP synthase of *S. diacarni* LHW51701^T^ recognized only the acetyl-ACP as its non-β-hydroxyl acyl substrate (50) for carbazomycin biosynthesis. The unprecedented *chlCz3* is a putative 3-oxoacyl-ACP synthase (KASIII) that possesses an acetyl-coenzyme A (CoA):ACP transacylase (ACAT) activity (51) for the catalytic formation of acetyl-ACP from acetyl-CoA and an acyl carrier protein, *chlCz4* (*nzsE* homolog in NZS biosynthesis), with 98.8% gene similarity to an ACP in *S. diacarni* LHW51701^T^. The acetyl-ACP may serve as a substrate for the side chain moiety of carbazomycins, since acetyl-ACP can be considered a starter unit for straight-chain fatty acid biosynthesis in *Streptomyces* (52). *chlCz3* catalyzed the condensation of α-hydroxy-β-keto acid and acetyl-ACP. The unstable intermediate formed by the *chlCz3*-catalyzed reaction would undergo cyclization and ring A formation of the carbazole nucleus mediated by the putative carbazole synthase *chlCz5* (*nzsI* homolog in NZS biosynthesis) that shared 100% gene homology to an SRPBCC family protein harbored by the *S. diacarni* LHW51701^T^ genome.

It was noted that **8** was a dihydroxyl type of carbazole metabolite, where its catechol intermediate **14** was initiated by dehydration to form **12** and nucleophilic aromatization to form **13** in a reaction catalyzed by *chlCz5* (*nzsI* homolog in NZS biosynthesis) (53) (Fig. 7). Interestingly, **1** produced by *S. tubbatahanensis* DSD3025^T^ was plausibly catalyzed from a mono-hydroxyl type of carbazole intermediate **15**, where the reduction and ring rearrangement steps were mediated by *chlCz5* (Table 5) (53). This unusual biosynthetic reaction catalyzed by *chlCz5* may uncover the key gene machinery to generate the putative intermediate **15** of chlocarbazomycin metabolites. The genomics, metabolomics, and bioinformatics analyses suggested that **1** and **8** may have a similar unstable intermediate, compound **11**, catalyzed by the carbazole synthase *chlCz5*, but they have different biosynthetic architectures in the carbazole ring A assembly (Fig. 7). The two-step oxidation and decarboxylation of **15** catalyzed by the putative *chlCz6a*/*chlCz6b* FAD binding or FAD dependent and the *chlCz7a/chlCz7b* SDR family oxidoreductases were plausibly initiated to remove the methyl groups attached in C-1 and C-2 of the carbazole ring A, forming intermediates **17** and **19**, respectively (Fig. 7). All the putative genes involved in the biosynthesis of **1** until intermediate **19** were found in BGC region 16 of the *S. tubbatahanensis* DSD3025^T^ genome (Fig. 8).

The hydroxyl group in C-3 of carbazole ring A in **1** was converted into a methoxy side chain catalyzed by a putative O-methyltransferase, *chlCz8* (93.2% gene similarity with a methyltransferase of *S. diacarni* LHW51701^T^), which resulted in the formation of a tricyclic carbazole, **20**, with no C-1 or C-2 methyl side chains (50). Interestingly, *chlCz8* was found outside the *chlCz* gene cluster (Table 5). The carbazole backbone of compounds **1** and **8** was mainly catalyzed by the putative KAS-III *chlCz3* and the putative carbazole synthase *chlCz5*, as in the case of neocarazostatin A (50).

The putative halogenase (*chlCz9*) gene involved in the chlorination at C-4 of carbazole ring A was proposed to be located outside the *chlCz* gene cluster. The *chlCz8* and *chlCz9* adjacent in the genome were located approximately 2.1 Mbp away from the *chlCz* gene cluster of DSD3025^T^ and had opposite gene orientations with their corresponding gene homologs in the LHW51701^T^ genome (Fig. 8).

In the search for a halogenase in *S. tubbatahanensis* DSD3025^T^ genome, the tryptophan 6-halogenase was thought to be the gene responsible for the chlorination of C-4 in **1**. However, the NMR analysis confirmed that the chlorine attachment in **1** was in C-4 of carbazole ring A and not in C-7 of the carbazole ring C, thus demonstrating that the tryptophan 6-halogenase was not responsible for this chlorination process. Notably, a putative single-component flavin-dependent halogenase (FDH), *chlCz9*, located outside the carbazomycin B gene cluster could be a bifunctional protein capable of chlorination and unusual flavin reduction. *chlCz9* was annotated by antiSMASH as a NAD(P)- and/or FAD-dependent oxidoreductase that may act on aromatic substrates (54) with tryptophan as a precursor, but not directly with tryptophan. The gene comparison in databases revealed 95.1% similarity with a NAD(P)- and/or FAD-dependent oxidoreductase harbored in the genome of a chlocarbazomycin producer, *S. diacarni* LHW51701^T^, and <50% gene similarity with halogenases. The NAD(P)- and FAD-dependent oxidoreductase as an FDH that could compensate for the chlorination of C-4 in carbazole ring A warrants further gene expression and *in vitro* experiments (54). More importantly, feeding 6-chlorotryptophan as a precursor for carbazomycin biosynthesis may unlock the possible role of tryptophan 6-halogenase found in the *S. tubbatahanensis* DSD3025^T^ genome in halogenating carbazole alkaloids.

The putative biosynthetic gene cluster in *S. tubbatahanensis* DSD3025^T^ consisted of *chlCz2/nzsH*, *chlCz3/nzsJ*, and *chlCz5/nzsI* homologs for the biosynthesis of carbazole alkaloids, which were conserved in neocarazostatin producer *Streptomyces* sp. MA37, carquinostatin producer *S. exfoliatus* 2419-SVT2, carbazomycin producer *S. luteoverticillatus* CGMCC 15060, and chlocarbazomycin producer *S. diacarni* LHW51701^T^ (Fig. 8). Additional biosynthetic genes were present in their respective BGCs for the modification of the carbazole nucleus and side chain moieties.

The bioinformatics analysis of the biosynthetic mechanism for the production of **1** highlighted the importance of exploring the different detection strictness and extra features predicted by antiSMASH. The careful analysis of proposed antiSMASH BGC boundaries could be a valuable aspect in mining biosynthetic gene clusters, rather than merely relying on the default settings. The putative gene cluster for the biosynthesis of **8** encoded in the *S. tubbatahanensis* DSD3025^T^ genome was identified through a detailed analysis of the BGC proposed by antiSMASH. The identified gene cluster was part of a larger BGC region 16 annotated as “T2PKS” due to its close proximity to signature genes used by antiSMASH as anchor points for the T2PKS gene cluster. The putative carbazomycin B gene cluster in the *S. tubbatahanensis* DSD3025^T^ genome was found using “relaxed” detection strictness, while the BGC for carbazomycin B in the *S. luteoverticillatus* CGMCC 15060 and *S. diacarni* LHW51701^T^ genomes was predicted only by antiSMASH in “loose” detection strictness.

### *S. tubbatahanensis* DSD3025^T^ extract induced cell death via membrane damage against the multidrug-resistant S. aureus ATCC BAA-44.

The crude extract of *S. tubbatahanensis* DSD3025^T^ had an oily consistency with dark brown coloration due to the high pigmented melanin content (55, 56). Initial screening using an absorbance-based assay revealed that the nature of *S. tubbatahanensis* DSD3025^T^ extract caused false-positive results and low reproducibility. Thus, a fluorescence-based assay was utilized in this study to remove the bias due to the nature of the extract.

The live/dead cell differentiation in a cell population can be easily distinguished using the calcein-propidium iodide (PI) staining (57). Calcein acetoxymethyl ester (calcein-AM) is a permeable nonfluorescent molecule that is rapidly hydrolyzed by intracellular esterases in viable cells to green fluorescent calcein, which is subsequently retained intracellularly (58). Alternatively, the nuclei-staining dye PI is a nonpermeable molecule that can only enter a membrane-damaged cell and intercalates with the DNA (59), producing a red fluorescence. Therefore, the effect of the extract on the cell viability and membrane integrity of multidrug-resistant S. aureus ATCC BAA-44 was determined via calcein-PI dual staining.

**FIG 9:**
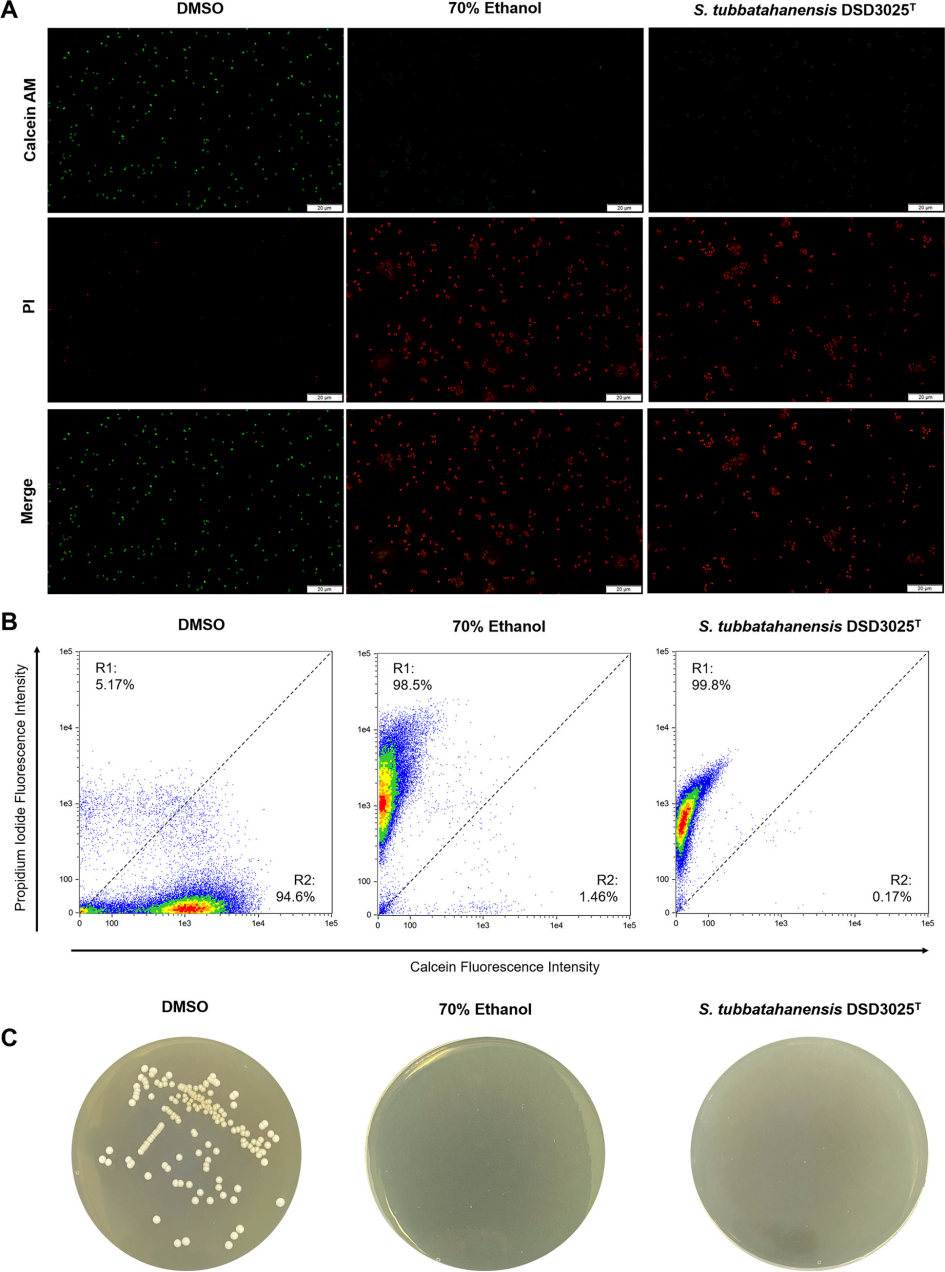
Effects of *S. tubbatahanensis* DSD3025^T^ extract (5 mg/mL) on the cell viability and membrane integrity of S. aureus ATCC BAA-44 after 6-h treatment exposure. (A) Fluorescence microscope images of multidrug-resistant S. aureus ATCC BAA-44 cells at ×100 magnification. Cells were stained with calcein-AM (green; live cells)-PI (red; dead cells) for live and dead cell differentiation. Scale bar, 20 μm. (B) Flow cytometric scatterplot profile of c-AM–PI-stained multidrug-resistant S. aureus ATCC BAA-44 cells. Regions R1 and R2 represent dead and live cells, respectively. (C) Total CFU in each treatment (*n* = 9) after 24 h of incubation.

After 6 h of treatment exposure, the live/dead cells were qualitatively identified via fluorescence microscopy. DMSO-treated cells displayed green fluorescence (Fig. 9A), indicating viable cells. On the other hand, cells treated with 70% ethanol and *S. tubbatahanensis* DSD3025^T^ extract (5 mg/mL) showed red fluorescence, indicating nonviable cells. The physiological status of the cell population of each treatment was then analyzed quantitatively by flow cytometric (FCM) analysis.

The cell population density in the flow cytometry profile in Fig. 9B was clustered into two regions based on their physiological status: R1, with PI-labeled cells (dead cells with damaged membranes), and R2, calcein-labeled cells (viable cells with an intact membrane) (23, 60). The DMSO-treated cells showed a high cell population (94.6%) in R2, which was considered live, viable cells. Results showed that *S. tubbatahanensis* DSD3025^T^ extract-treated cells exhibited a high cell population (99.8%) in R1, indicating nonviable cells. The bactericidal activity of *S. tubbatahanensis* DSD3025^T^ extract was significantly comparable to that in 70% ethanol, which killed 98.5% of the cell population. These cells were considered dead with damaged membranes, as propidium iodide can only penetrate bacterial cells with compromised membrane permeability or integrity. In this study, 70% ethanol was used as a positive model for cell membrane damage. Ethyl alcohol, at 60 to 80% concentration, is a known disinfecting agent that denatures proteins and disrupts the cell membrane. The loss of cellular membrane integrity results in increased permeability and uncontrolled transport of solutes, decreasing the proton flux across the membrane and cytoplasmic leakage (61–63) leading to cell death.

The bactericidal activity of *S. tubbatahanensis* DSD3025^T^ extract at 5 mg/mL was further validated by standard total plate counts to confirm that the membrane-damaged cell population observed in flow cytometry was dead cells. An antibiotic agent is considered “bactericidal” if it can completely prevent bacterial growth or results in a ≥99.9% decrease in the initial inoculum (64). Interestingly, both ethanol-treated and *S. tubbatahanensis* DSD3025^T^ extract-treated cells showed no visible growth after 24 h of incubation. Compared to DMSO-treated cells, which showed bacterial growth with 2.2 × 10^8^ CFU/mL (Fig. 9C). Collectively, these results confirmed that the *S. tubbatahanensis* DSD3025^T^ extract induced cell death of S. aureus ATCC BAA-44 via membrane damage.

Compound **1** produced by DSD3025^T^ was tested against ESKAPE pathogens. The results showed that **1** exhibited antibacterial activity against S. aureus ATCC BAA-44 (98% growth inhibition) and S. pyogenes (95% growth inhibition), with MIC_90_ values of 128 μg/mL and 64 μg/mL, respectively (Table 6). Minimal growth inhibition was observed against E. faecium, with only 87% growth inhibition at 128 μg/mL, while none of the Gram-negative pathogens was inhibited. Carbazole derivatives have attracted attention due to their wide range of pharmacological applications (65–69), as the carbazole moiety is known to be useful in research and clinical studies (69–73). The compound **1** produced by *S. diacarni* LHW51701^T^ has a MIC of >128 μg/mL against Bacillus mycoides, methicillin-resistant S. aureus (MRSA), Mycobacterium smegmatis, and Candida albicans (45). In addition, compound **1** produced by coral reef sand-derived *S. diacarni* SCSIO 64983, when tested using the disk-diffusion method at 10 μg, showed no antibacterial activity against Gram-positive or Gram-negative pathogens. Halo- and thio-carbazomycin analogs produced by *S. diacarni* SCSIO 64983 against the mentioned test pathogens also showed no antibiotic properties (43). The observed antibacterial activities of **1** produced by *Streptomyces tubbatahanensis* DSD3025^T^ in this study against disease-causing pathogens S. aureus and S. pyogenes showed a propitious result that fills in the gap for antibiotic drug discovery.

**TABLE 6:** MIC_90_ of compound **1** against multidrug-resistant pathogens^*a*^

Treatment	MIC_90_ value (μg/mL)					
	E. faecium ATCC 700221	S. aureus ATCC BAA-44	K. pneumoniae ATCC BAA-1705	A. baumannii ATCC BAA-1605	P. aeruginosa ATCC BAA-1744	S. pyogenes ATCC 12384
Compound **1**	>128	128	>128	>128	>128	64
Imipenem			64		8	
Meropenem				64		
Tetracycline	0.5	16	8	64	32	0.5
Vancomycin	>128	2				0.5

a Positive controls included imipenem (tested against K. pneumoniae and P. aeruginosa), meropenem (tested against A. baumannii), and vancomycin (tested against E. faecium, S. aureus, and S. pyogenes).

### *S. tubbatahanensis* DSD3025^T^ extract exhibited dose-dependent antiproliferative activities and toxicities against cancer and nontumor cells.

**FIG 10:**
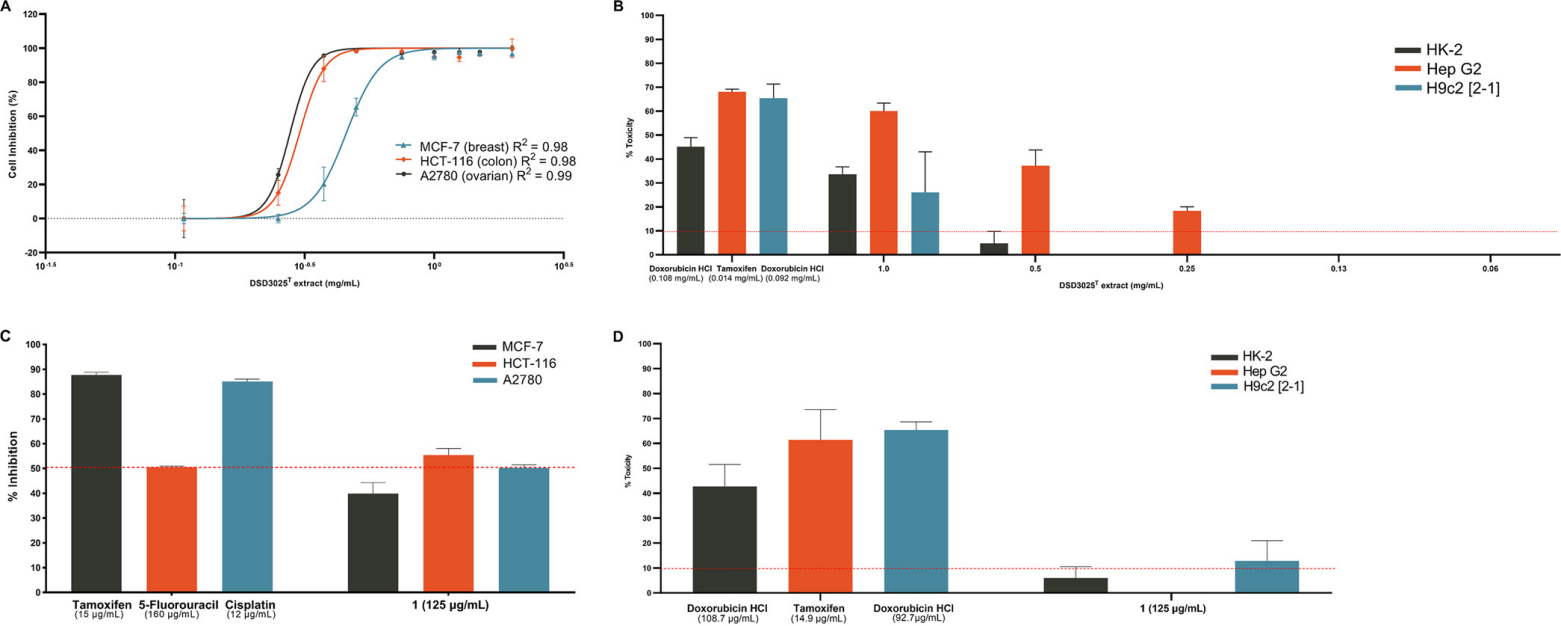
Antiproliferation (MTT) and toxicity assays of *S. tubbatahanensis* DSD3025^T^. (A) Antiproliferative activity of *S. tubbatahanensis* DSD3025^T^ extract against MCF-7, HCT-116, and A2780 cell lines. (B) Toxicity results of *S. tubbatahanensis* DSD3025^T^ extract against kidney (HK-2), liver (Hep G2), and cardiac (H9c2 [2-1]) cell lines. (C) Antiproliferative activity of **1** isolated from of *S. tubbatahanensis* DSD3025^T^ versus the positive control (tamoxifen, cisplatin, and 5-fluorouracil) against MCF-7, HCT-116, and A2780 cell lines, respectively. (D) Toxicity results for **1** versus positive control, doxorubicin HCl (HK-2, H9c2 [2-1]), and tamoxifen (Hep G2) cell lines.

The 3-(4,5-dimethyl-2-thiazolyl)-2,5-2H-tetrazolium bromide (MTT) assay is one of the most exploited *in vitro* cytotoxicity assays used in cancer research for measuring cell viability (74) and in the determination of the anticancer potential of a new compound (75, 76). The antiproliferative activities of *S. tubbatahanensis* DSD3025^T^ extract as well as that of **1** were determined against MCF-7, HCT-116, and A2780 cancer cell lines in MTT assays. *S. tubbatahanensis* DSD3025^T^ extract demonstrated antiproliferative activity in a dose-dependent manner, exhibiting a complete inhibitory activity against all cancer cells at 2 mg/mL (Fig. 10A). The *S. tubbatahanensis* DSD3025^T^ extract had 50% inhibitory concentration (IC_50_) values of 457.4 μg/mL for MCF-7, 302.00 μg/mL for HCT-116, and 277.50 μg/mL for A2780 cells. Regardless of the IC_50_ value of the extract, its inhibitory activity still showed an anticancer potential, suggesting that further purification of the extract may increase its anticancer activity.

Compound **1** demonstrated anticancer activity above the 50% threshold against two cancer cell lines. At 125 μg/mL, **1** showed 55.4% and 50.2% growth inhibition against HCT-116 and A2780 cells, respectively (Fig. 10C). Carbazole derivatives have been gaining interest for their wide range of biological activities (64–66). However, **1** isolated from the sponge-associated bacterium *Streptomyces diacarni* LHW51701^T^ demonstrated no anticancer activity against the human lung adenocarcinoma cell line SPCA-1 (45). In this study, the antiproliferative activities of **1** produced by *Streptomyces tubbatahanensis* DSD3025^T^ against HCT-116 and A2780 indicated that the compound had a broader spectrum for targeted cancer cell lines.

The *S. tubbatahanensis* DSD3025^T^ extract showed decreasing toxicity in a dose-dependent manner against kidney, liver, and cardiac cell lines (Fig. 10B). Moreover, **1** showed moderate nephrotoxicity, high cardiotoxicity, and nonhepatotoxicity at 125 μg/mL (Fig. 10D). A low percent toxicity relates to safe levels of a compound suitable for administration toward the target organ. On the other hand, absence of compound toxicity signifies viability of the cells, since elevated LDH levels are associated with cell injury and death.

### Conclusions.

Advancements in bioinformatics and genomics have improved the ability to analyze key features and core biosynthetic gene clusters of novel genomes. These major developments in natural products research have facilitated the discovery of novel compounds through metabolomics. The discovery of the tryptophan halogenase gene encoded in the novel *S. tubbatahanensis* DSD3025^T^ genome enabled the identification of halogenated compounds by high-resolution mass spectrometry. The mass spectrometry metabolomics demonstrated that *S. tubbatahanensis* DSD3025^T^ produces halogenated metabolites, which could be investigated for their anticancer and antibacterial activity. The halogenated metabolites produced by *S. tubbatahanensis* DSD3025^T^ were less likely attributed to the original tryptophan halogenase harbored in the genome, and this finding led to the focused search for halogenated compounds using metabolomics. The two-component halogenase-reductase system was not involved in the halogenation of carbazole metabolites produced by *S. tubbatahanensis* DSD3025^T^, but the chlorination step was rather compensated by a putative FDH gene that facilitated the C-4 chlorination of carbazole ring A. The tryptophan halogenase and its associated flavin reductase encoded in the *S. tubbatahanensis* DSD3025^T^ genome may influence the halogen moiety of other tryptophan substrates, and this needs further investigation for the identification of novel specialized metabolites. Future purification and NMR analysis of *S. tubbatahanensis* DSD3025^T^ extract warrant the profiling, structure elucidation, and identification of the halogenated specialized metabolites as antibacterial and anticancer candidates in the drug discovery pipeline.

The integration of bioinformatics-driven genomics and metabolomics unearths the hidden biosynthetic gene machineries that will further accelerate the natural drug discovery in novel *Streptomyces*. Overall, the bioprospecting of novel *Streptomyces* species from marine sediments of underexplored ecological niches serves as an important source of drug leads with hidden biosynthetic potential and unique chemical scaffolds.

### Description of *Streptomyces tubbatahanensis* sp. nov.

*Streptomyces tubbatahanensis* (tub.ba.ta.ha.nen’sis. N.L. masc. adj. tubbatahanensis pertaining to Tubbataha, a marine natural park in Palawan, Philippines, where the type strain was isolated).

It is a Gram stain-positive and aerobic actinomycete that forms well-developed, branched substrate mycelia with intertwining hyphal cells in MM1 agar and grows well in YGC, NA, TSA, ISP2, and ISP3 media. *S. tubbatahanensis* DSD3025^T^ has moderate growth in MM11 and ISP9 and poor growth in MM3 and ISP4. Optimum growth is at 28°C, pH 4.0 to 10.0, and 0 to 15% (wt/vol) NaCl. In the API ZYM, it is positive for alkaline phosphate, esterase, leucine arylamidase, acid phosphatase, and naphthol phosphohydrolase and weakly positive for esterase-lipase and α-glucosidase enzymes.

The major menaquinone systems are MK-9 (H8), MK-9 (H6), and MK-9 (H4). The predominant fatty acids are C_16:0_, C_18:1ω9c_, and summed feature 5. The main polar lipids are unidentified phospholipids, followed by phosphatidylethanolamine, aminophospholipids, diphosphatidylglycerol, glycolipids, and unidentified lipids. The whole-cell hydrosylate contains glucose and xylose.

The type strain, DSD3025^T^ (=DSM 33792^T^), was isolated from the marine sediment samples collected in Tubbataha Reefs Natural Park, Cagayancillo, Palawan, in the middle of Sulu Sea, Philippines. The complete genome of *S. tubbatahanensis* DSD3025^T^ is composed of one linear chromosome 7,760,770 bp long, with a G+C content of 72.3%, a total of 6,579 predicted genes, and 29 biosynthetic gene clusters. The whole-genome sequence of *S. tubbatahanensis* DSD3025^T^ has been deposited in the GenBank database under the accession number CP093846.

## MATERIALS AND METHODS

### Strain isolation and maintenance.

*S. tubbatahanensis* DSD3025^T^ was isolated from a marine sediment sample at a collection site (latitude 8.74000000, longitude 119.81916667) in Tubbataha Reefs Natural Park in the middle of Sulu Sea, Philippines, in April 2018. *S. tubbatahanensis* DSD3025^T^ was recovered from marine sediment in the subsurface layer (26 to 50 cm depth below the seafloor) sampled using a 110-cm core sampler (22). Marine sediment samples were air-dried completely and inoculated in a trehalose-containing marine agar using the dry stamp method. Pure culture of *S. tubbatahanensis* DSD3025^T^ was obtained and maintained on MM1 (77) agar at 28°C and stocked in glycerol stock suspensions (20% [vol/vol]) at −80°C.

### Genomic and phylogenetic analyses.

The genomic DNA extraction of *S. tubbatahanensis* DSD3025^T^ was carried out by using DNeasy blood and tissue kits (Qiagen) (21). Whole-genome sequencing of *S. tubbatahanensis* DSD3025^T^ was performed by applying a long-read PacBio single-molecule real-time (SMRT) sequencing system (78). The SMRTbell template library was based on the procedure for 10-kb library preparation according to the instructions from PacBio. The size selection for BluePippin was performed according to the manufacturer’s recommendations using the 0.75% DF Marker S1 High-Pass 6-kb to 10-kb v3 run protocol and S1 marker (79). The SMRTbell library was annealed based on the SMRT link setup and then sequenced using Sequel II. For whole-genome analyses, *S. tubbatahanensis* DSD3025^T^ was sequenced with its genome assembled and deposited at NCBI GenBank under the accession number CP093846.

The complete 16S rRNA gene sequence of *S. tubbatahanensis* DSD3025^T^ (1,531 bp) was retrieved from the draft genome sequence data and initially analyzed using the EzBioCloud server (80). For phylogenetic analysis, the 16S rRNA gene sequences of the type strains of closely related *Streptomyces* species were obtained from the NCBI GenBank database. The 16S rRNA gene sequences were aligned using the Clustal W algorithm in MEGA 11.0 (81). The phylogenetic trees were constructed using neighbor-joining (82), maximum parsimony (83), and maximum likelihood (84) methods with bootstrap analysis based on 1,000 replications. The five housekeeping genes, *atpD* (ATP synthase F1 beta subunit), *gyrB* (DNA gyrase B subunit), *rpoB* (RNA polymerase beta subunit), *recA* (recombinase A), and *trpB* (tryptophan synthetase beta subunit) were retrieved from the complete genome sequence of *S. tubbatahanensis* DSD3025^T^, and the related gene sequences of the type strains were obtained from the GenBank database and concatenated head-to-tail, in-frame. The phylogenetic tree of the concatenated protein-coding sequence (*atpD-gyrB-rpoB-recA-trpB*) of *S. tubbatahanensis* DSD3025^T^ and its closely related strains in the GenBank database was reconstructed using neighbor-joining (82), maximum parsimony (83), and maximum-likelihood algorithms (84) in MEGA 11.0 (81). The multilocus sequence analysis (MLSA) evolutionary distances were calculated using Kimura’s two-parameter model (85). The *in silico* digital DNA-DNA hybridization (dDDH) values, based on formula 2, were calculated using the Genome-To-Genome Distance Calculator (GGDC v3.0) (86) at https://ggdc.dsmz.de/ggdc.php#. Calculation of orthoANI values and generation of an ANI heat map were performed by using OAT software v0.93.1 available at https://www.ezbiocloud.net/tools/orthoaniu (87).

### Cultural and phenotypic properties.

The cultural characteristics of *S. tubbatahanensis* DSD3025^T^ were determined following the growth in MM1 agar, mannitol-containing agar (MM3), glucose-containing (MM11) agar, YGC (88) agar, TSA, NA, and ISP2, ISP3, ISP4, and ISP9 at 28°C for 7 days. The morphological characteristics of *S. tubbatahanensis* DSD3025^T^ were observed in terms of aerial spore color, vegetative mycelium, and diffusible pigmentation produced in MM1 agar after 7 days of incubation at 28°C and with scanning electron microscopy (JEOL JSM 5510LV). Growth at different pH, temperature, and salinity was observed in MM1 agar. *S. tubbatahanensis* DSD3025^T^ was grown in MM1 agar with different pH levels ranging from 4.0 and 10.0 at an interval of 1 pH unit. The temperatures used to determine the growth of *S. tubbatahanensis* DSD3025^T^ were 4, 28, and 37°C. Tolerance to NaCl was carried out by growing *S. tubbatahanensis* DSD3025^T^ in MM1 agar with different NaCl concentrations (0, 2.6, 3, 5, 7, 10, 12, 15% [wt/vol]). Enzymatic and biochemical characteristics of *S. tubbatahanensis* DSD3025^T^ were analyzed using a commercially available API ZYM kit (bioMérieux, Marcy-l’Étoile, France) (89–91). The API strips were inoculated according to the manufacturer’s instructions. The reference strain, Pseudomonas aeruginosa ATCC 27853, was used as a standard control for the assay. Briefly, *S. tubbatahanensis* DSD3025^T^ and P. aeruginosa bacterial suspensions were prepared in an API suspension medium with a turbidity of McFarland standard 5. A volume of 65 μL of bacterial suspension was inoculated into each cupule of the API strips. The strips were then incubated for 4 h at 37°C. One drop of ZYM A and ZYM B reagents was added into each cupule for the enzyme and biochemical analyses.

### Chemotaxonomic properties.

The biomass and lyophilized cells of *S. tubbatahanensis* DSD3025^T^ were obtained by growing in MM1 broth at 28°C for 7 days in a shaking incubator (15, 21–23, 92, 93). The analyses of fatty acids (92, 93), polar lipids (94), whole sugar (95), and respiratory quinones were carried out by the Identification Service, Leibniz-Institut DSMZ-Deutsche Sammlung von Mikroorganismen und Zellkulturen GmbH, Braunschweig, Germany. The cellular fatty acids were analyzed according to the instructions of the Microbial Identification System (MIDI; microbial ID). Polar lipids were extracted from lyophilized cells using chloroform–methanol–0.3% aqueous NaCl mixture (94) and identified by two-dimensional silica gel thin-layer chromatography. Diagnostic sugars in whole-cell hydrolysates (96) were analyzed by thin-layer chromatography on cellulose plates for identification. Respiratory quinones were extracted from freeze-dried cells using hexane, purified further by a silica-based solid-phase extraction, and identified by HPLC-DAD based on confirmed spectrum and retention time in MS. *S. tubbatahanensis* DSD3025^T^ was deposited in DSMZ under the accession number DSM 33792^T^.

### Bioinformatics analysis and BGC evaluation.

The genome of *S. tubbatahanensis* DSD3025^T^ was annotated using EzBioCloud platforms (80) and Rapid Annotation using Subsystem Technology (RAST) software (https://rast.nmpdr.org/rast.cgi?page=Upload) (97). Predicted operons in the *S. tubbatahanensis* DSD3025^T^ genome were identified using the Operon-mapper (98) available at https://biocomputo.ibt.unam.mx/operon_mapper/. The predicted genomic islands that were thought to have horizontal origins were predicted using IslandViewer v4 at https://www.pathogenomics.sfu.ca/islandviewer/upload/ (99). The *in silico* resistome of *S. tubbatahanensis* DSD3025^T^ was predicted using the Resistance Genes Identifier (RGI, v5.1.1) based on the Comprehensive Antibiotic Resistance Database (CARD, v3.1.1) (100) available at https://card.mcmaster.ca/analyze/rgi. The detections of core genes and known resistance models associated with BGCs were performed using Antibiotic Resistant Target Seeker (ARTS, v2) (101) at https://arts.ziemertlab.com/analyze. Specialized metabolite BGCs were predicted and analyzed using antiSMASH v7.0 at https://antismash.secondarymetabolites.org/ (102). Genome mining for lanthipeptides and precursor peptides was inferred using BAGEL v4.0 (103) at http://bagel4.molgenrug.nl/. The structures of predicted biosynthetic gene clusters were assembled using Prediction Informatics for Secondary Metabolomes (PRISM, v4) (104) at https://prism.adapsyn.com/.

### Extract preparation.

A 7-day-old broth culture of *S. tubbatahanensis* DSD3025^T^ was inoculated onto MM1 agar and grown for 14 days at 28°C. Harvested biomass was extracted with ethyl acetate and concentrated *in vacuo* until dried extract was obtained (15, 21–23). Solid-phase extraction (SPE) was performed to remove methanol (MeOH)-insoluble melanin and impurities in the extract. The dried extract was reconstituted in 100% MeOH (HPLC grade) to create a to 40-mg/mL suspension and then semipurified using Sep-Pak Plus Short C_18_ cartridges (Waters, Ireland) eluted with 100% MeOH. The *S. tubbatahanensis* DSD3025^T^ SPE extract was concentrated *in vacuo* for metabolite profiling, antibiotic testing, and anticancer screening.

### UPLC-QTOF of *S. tubbatahanensis* DSD3025^T^ extract.

The chemical profile of *S. tubbatahanensis* DSD3025^T^ extract was analyzed using MS in UPLC-QTOF-MS. A 0.2-mg/mL solution of *S. tubbatahanensis* DSD3025^T^ extract in MeOH (MS grade) was prepared and placed in an autosampler at 4°C. Five microliters of the solution was injected into the UPLC system. Separation of compounds was performed using an Acquity UPLC BEH C_18_ column (130 Å, 1.7 μm, 2.1 mm by 50 mm) held at 40°C. Water (H_2_O; solvent A) and acetonitrile (MeCN; solvent B) containing 0.1% (vol/vol) formic acid (HCOOH) were used as mobile phase. The mobile phase was pumped at 0.3 mL/min as follows: 20% B (0 to 0.55 min), 20% to 100% B (0.55 to 9.92 min), 100% B (9.92 to 11.58 min), 100% to 20% B (11.58 to 12.68 min), and 20% B (12.68 to 13.78 min). Compounds eluted at different retention times were analyzed using a Waters Synapt XS Q-ToF mass spectrometer equipped with an ESI source. The mass spectrometer was calibrated in both positive and negative ions in resolution mode using sodium iodide (NaI). A lockspray mass correction was performed using leucine-enkephalin (*m/z* 556.2771 [M + H]^+^, *m/z* 554.2615 [M − H]^−^). The MS^E^ data (low energy, 0 eV; high energy, ramp 25 to 75 eV) was acquired in the centroid mode over a mass ion range of 100 to 2,100 Da and a scan time set at 0.15 s. The analyte was subjected to a capillary voltage of 3 kV (positive) or 1.0 kV (negative), with a 100°C source temperature, 500 liters/h desolvation gas (N_2_) flow, and 300°C desolvation temperature. Acquired MS^E^ data were processed using MassLynx software version 4.2 (Waters Corporation, Milford, MA, USA). Accurate mass, isotope pattern, chemical formula, and double bond equivalent were used for extensive library search in AntiBase v2017, ChemSpider, and MarinLit.

### Molecular networking analysis.

The acquired Waters MS^E^ data (.raw) was converted to Analysis Base File (.abf) format using the Reifycs Analysis base file converter (Tokyo, Japan). The converted files were then processed using MS-DIAL with optimized parameters (Table S12).

A library of 12,879 unique compounds in the negative mode was downloaded from http://prime.psc.riken.jp/compms/msdial/main.html for the identification of compounds. Molecular networking analysis was performed using the Data Visualization Navigator on a target *m/z* value, and the MS/MS tolerance was set at 0.05 Da, 40% cutoff similarity, and retention time tolerance to 1.5 min. The molecular network was then visualized using the Mozilla Firefox HTML viewer.

### Isolation and structural elucidation of chlocarbazomycin A compound 1 produced by *S. tubbatahanensis* DSD3025^T^.

A solid-phase extraction was performed with 1 g dried *S. tubbatahanensis* DSD3025^T^ crude extract via reversed-phase flash column chromatography (Biotage Isolera, Biotage Sfar, C_18_, 100 Å, 30 μm, 30 g, 135 mL H_2_O, 135 mL MeOH, 25 mL/min). The H_2_O eluate was lyophilized and stored in a –20°C freezer, while the MeOH eluate was dried *in vacuo* at 35°C to afford 877 mg dried *S. tubbatahanensis* DSD3025^T^ MeOH eluate. The dried MeOH eluate was subjected to mass-directed purification via reversed-phase preparatory HPLC to isolate **1** (DSD3025H1; *t_R_* = 44. 97 min) using an Xbridge Prep C_18_ column (5 μm, 19 by 150 mm) and H_2_O/MeOH as mobile phase pumped as follows: 80:20 H_2_O-MeOH (0 to 4.43 min), linear gradient elution to 100% MeOH (4.43 to 61.95 min), 100% MeOH (61.95 to 72.15 min), back to 80:20 H_2_O-MeOH (72.15 to 78.90 min), and equilibration at 80:20 H_2_O-MeOH (78.90 to 85.65 min). A Waters Prep HPLC system was used (Waters 2535 quaternary pump, manual injector) equipped with a make-up pump (Waters 515) and a splitter that split the column outlet flow to the single-quadrupole mass detector (Waters Acquity QDa detector) and fraction collector (WFC III) at 1:1,000 (vol/vol). Mass ion scans were set at a range of 150 to 700 *m/z* in the positive polarity. The fraction collection was triggered at *m/z* 232 [M + H]^+^, >1 × 10^3^ intensity. The collected fraction was dried *in vacuo* to afford 4.73 mg of **1** at ~80% purity as estimated via ^1^H NMR.

The structure of **1** was described and verified using NMR data. Briefly, the one-dimensional (1D) and 2D NMR spectra of **1** were recorded in CDCl_3_ (99.8% D) at 298 K with CDCl_3_ peaks as a reference on a Bruker Avance cryoprobe (5 mm TCI ^1^H&^19^F/^13^C&^15^N/D) NMR spectrometer operating at 600 MHz for ^1^H and 150 MHz for ^13^C. The software used in the acquisition was Bruker TopSpin software (version 4.1.4), while the postprocessing of data was performed on MestreNova software (version 14.2.2). Standard Bruker pulse sequences were used in all 1D and 2D NMR experiments (^1^H, ^1^H-decoupled ^13^C, ^13^C DEPTQ135, COSY, HSQC, HMBC, and NOESY). The HMBC experiment was optimized using the *J*_CH_ value of 6 Hz, while the mixing time for the NOESY experiment was 750 ms. The 1D and 2D NMR spectra were calibrated based on residual CDCl_3_ signals (^1^H, δ 7.24 ppm; ^13^C, δ 77.2 ppm).

### Antibacterial activity.

**(i) Flow cytometry assay by calcein-PI dual staining.** The effect of *S. tubbatahanensis* DSD3025^T^ on bacterial cell viability and membrane integrity was assessed using a calcein-PI dual staining assay, followed by fluorescence microscopy and flow cytometry. The antibiotic activity of *S. tubbatahanensis* DSD3025^T^ extract was evaluated against a multidrug-resistant pathogen, Staphylococcus aureus ATCC BAA-44, an Iberian MRSA clone with multidrug resistance against 18 antibiotics (52). Briefly, S. aureus ATCC BAA-44 cells were grown for 3 h at 37°C (200 rpm) to obtain mid-logarithmic-phase cells. The cells were harvested by centrifugation (4,000 rpm for 5 min) and resuspended in phosphate-buffered saline (PBS) to a final optical density at 600 nm (OD_600_) of 0.5. In a 96-well plate, the bacterial suspension in PBS was treated with *S. tubbatahanensis* DSD3025^T^ extract at 5 mg/mL. DMSO- and 70% ethanol-treated cells were used as the negative and positive controls, respectively. Treatment plates were incubated for 6 h at 37°C (80 rpm). A premixed dye solution was prepared using a Live/Dead cell double staining kit (Sigma-Aldrich, MO, USA) according to the manufacturer’s protocol with modifications. The dye solution was prepared by adding 5 μL of calcein-AM (CA) and 20 μL of PI to 24 mL PBS. After treatment exposure, cells were dual-stained by adding 10 μL CA-PI dye solution. Single-stained cells were also prepared for fluorescence compensation in the flow cytometer. The plate was incubated for 30 min in a dark room at 37°C.

The flow cytometry analysis was performed to quantify the live or dead cells in the cell population. Data were acquired using Amnis FlowSight imaging flow cytometer (Luminex, Austin, TX, USA) with 505 to 560 nm (channel 2) and 642 to 745 nm (channel 5) for CA and PI fluorescence detection, respectively. Data acquisition was set to 10,000 events for each sample. The experiment was performed in three trials with triplicates. Data were analyzed using AMNIS IDEAS software v6.3.23. The PI fluorescing cells (R1) were gated as dead cells with damaged cell membranes, and CA fluorescing cells (R2) were gated as live cells with intact membranes. Treated cells were also observed in an IX83 inverted fluorescence microscope (Olympus, USA) to morphologically identify the physiological status of the cells through fluorescence emission.

**(ii) Total plate count.** A total plate count was performed using the spread plate method to confirm the bactericidal activity. Cell treatment was performed using the same protocol as described above. The treated cells were serially diluted up to 1 × 10^6^ dilution in PBS. The cell suspension (50 μL) was then inoculated onto TSA plates and incubated for 24 h at 37°C. Three trials with triplicates were done. CFU were calculated using the following equation: CFU per milliliter = [(number of colonies)(dilution factor)]/(volume of culture plate).

**(iii) MIC of chlocarbazomycin A compound 1.** The MIC_90_ of **1** was determined using a microbroth susceptibility assay with 2-fold serial dilution. The compound was tested starting from an initial test concentration of 128 to 0.25 μg/mL against multidrug-resistant pathogens Enterococcus faecium ATCC 70021, Staphylococcus aureus ATCC BAA-44, Klebsiella pneumoniae ATCC BAA-1705, Acinetobacter baumannii ATCC BAA-1605, and Streptococcus pyogenes ATCC 12384. The positive controls were vancomycin for E. faecium, S. aureus, and S. pyogenes, meropenem for A. baumannii, imipenem for K. pneumoniae, and tetracycline for all test pathogens. DMSO was used as the negative control. The bacterial suspension (195 μL) with an OD_600_ of 1 × 10^6^ CFU/mL was dispensed into the wells of a 96-well plate followed by the addition of the treatment (5 μL), and then incubated for 18 to 24 h at 37°C. The OD_600_ was determined using an absorbance microplate reader (BioTekTM ELx808, Biotek, Winooski, VT, USA). The last concentration showing >90% growth inhibition was considered the MIC_90_ of the extract (53). The assay was performed in triplicates and three trials except for S. pyogenes, which was tested in two trials only, due to sample limitation. The percent growth inhibition was calculated using the following equation: percent growth inhibition = [(OD_negative control_ − OD_treatment_)/(OD_negative control_)] × 100.

### Anticancer activity.

**(i) Cell line maintenance.** Human ovarian carcinoma (A2780; ECACC 93112519), human colon carcinoma (HCT-116; ATCC CCL-247), and human breast Caucasian adenocarcinoma (MCF-7; ECACC 86012803) were used for MTT testing. The human renal proximal tubule epithelial cell line (HK-2; ATCC CRL-2190), human hepatocellular carcinoma (Hep G2; ECACC 85011430), and rat cardiomyocytes (H9c2 [2-1]; ECACC 88092904) were utilized for toxicity testing. The A2780 cells and HCT-116 cells were grown in RPMI 1640 (D6456). The MCF-7 and Hep G2 cells were grown in minimum essential medium (MEM; 51411C) with 1% nonessential amino acid solution (NEAA; M7145). The MCF-7 growth medium had an added supplement of 1 mM sodium pyruvate solution (S8636). The HK-2 and H9c2 [2-1] cell lines were grown in Dulbecco’s minimum essential medium (DMEM; D6546). All maintenance media contained 10% fetal bovine serum (F2442), 2 mM alanyl-glutamine (A8185) supplement, and 1% penicillin-streptomycin (P4333), with the exception of MCF-7 and HCT-116 growth media, which did not contain 2 mM alanyl-glutamine supplement.

The cell lines used in this study were cultured in accordance with the medium components and concentrations publicly listed in the European Collection of Authenticated Cell Cultures (ECACC) and American Type Culture Collection (ATCC). The growth media and supplements used for cell line maintenance were purchased from Merck (Sigma-Aldrich), Philippines.

**(ii) MTT assay.** Human cancer cells for the antiproliferation assay of *S. tubbatahanensis* DSD3025^T^ extracts and **1** were seeded in 96-well plates using an automated liquid workstation Biomek i5 (Beckman Coulter, Germany) at 25,000, 6,000, and 8,000 cells/well for A2780, HCT-116, and MCF-7, respectively. Cells were incubated for 24 h at 37°C with 5% CO_2_ and 80 to 90% relative humidity. The first batch of cells was then treated with 2-fold dilutions of *S. tubbatahanensis* DSD3025^T^ extract from 2 to 0.007 mg/mL, while another batch was treated with 2-fold dilutions of **1** at 125 to 0.49 μg/mL for 24 h. The negative control was DMSO, while cisplatin (PHR1624), 5-fluorouracil (F6627), and tamoxifen (85256) were used as positive controls for A2780, HCT-116, and MCF-7 cancer cells, respectively. Ten microliters of a 5-mg/mL MTT solution was then added to each well and incubated for 4 h. The medium was then gently aspirated and replaced with 100 μL DMSO to dissolve the formazan crystals. Absorbance was measured at 570 nm using a CLARIOstar multimode microplate reader (BMG Labtech GmbH, Germany). The percent growth inhibition was calculated using the following equation: percent inhibition = {[(negative control absorbance) − (experimental absorbance)]/(negative control absorbance)} × 100.

The IC_50_ values of *S. tubbatahanensis* DSD3025^T^ extract were analyzed using GraphPad Prism v9.5.0 for Windows (GraphPad Software, San Diego, CA USA). The inhibitory activity of **1** tested at a final concentration of 125 μg/mL was compared with that of the positive controls using the same software.

**(iii) Lactate dehydrogenase (LDH) assay for toxicity profiling with nontumor cells.** Cell lines for the LDH assay were seeded at 5,000 cells/well in a 96-well plate using an automated liquid handling workstation (Biomek i5; Beckman-Coulter, Germany). The experimental plates were incubated for 2 to 4 h before the addition of treatment for the cells to attach at 37°C, 5% CO_2_ with a relative humidity of 80 to 90%. After incubation, the 2-fold dilutions of *S. tubbatahanensis* DSD3025^T^ SPE extract and **1** with decreasing concentrations starting from 1 mg/mL to 0.063 mg/mL, and 125 μg/mL were added, respectively.

The negative control used was 0.1% DMSO. Doxorubicin hydrochloride (PHR1789) was used as the positive control for HK-2 and H9c2 [2-1] cell lines, while tamoxifen (85256) was used as the positive control for the Hep G2 cell line. The experimental plates were incubated for 18 h followed by LDH toxicity testing using a cytotoxicity detection kit (4744934001; Roche Diagnostics). The stop solution used was 1 N HCl, and the absorbance was measured at 490 nm using a CLARIOstar multimode microplate reader (BMG Labtech GmbH, Germany). The percent toxicity was calculated using the following formula which was based on the assay kit protocol: percent toxicity = {(experimental value − experimental blank) − (DMSO control − DMSO control blank)]/[(high control value − high control blank) − (low control value − low control blank)]} × 100. The results were illustrated using GraphPad Prism v9.5.0 for Windows (GraphPad Software, San Diego, CA, USA).

The threshold value for analyzing the toxicity in this study was set to 10%, to obtain the lowest minimum compound efficacy against the tested cell line. Any value exceeding the baseline of 10% was considered toxic.

### Data availability.

The whole genome of *S. tubbatahanensis* DSD3025^T^, consisting of 7,760,770 bp and with 72.3% G+C content (Table 1), was deposited in the GenBank database under accession number CP093846.
