# Supplementary material for: Synergy between Genome Mining, Metabolomics, and Bioinformatics Uncovers Antibacterial Chlorinated Carbazole Alkaloids and Their Biosynthetic Gene Cluster from Streptomyces tubbatahanensis sp. nov., a Novel Actinomycete Isolated from Sulu Sea, Philippines
Source: Microbiol Spectr. 2023 Feb 21;11(2):e03661-22. doi: 10.1128/spectrum.03661-22 (PMC10100901; doi:10.1128/spectrum.03661-22)
Supplement: Supplemental file 1 — Supplemental material. Download spectrum.03661-22-s0001.pdf, PDF file, 8.8 MB [file spectrum.03661-22-s0001.pdf]

Synergy between Genome Mining, Metabolomics, and Bioinformatics Uncovers Antibacterial Chlorinated Carbazole Alkaloids and their Biosynthetic Gene Cluster from *Streptomyces tubbatahanensis* sp. nov., a Novel Actinomycete Isolated from Sulu Sea, Philippines

Chuckcris P. Tenebro[1], Dana Joanne Von L. Trono[1], Lex Aliko P. Balida[1], Leah Katrine A. Bayog[1], Julyanna R. Bruna[1], Edna M. Sabido[1], Dion Paul C. Caspe[2], Emmanuel Lorenzo C. de Los Santos[3,4], Jonel P. Saludes[2,4,5], Doralyn S. Dalisay[1,4,6*]

[1]Center for Chemical Biology and Biotechnology (C2B2), University of San Agustin, Gen. Luna Street, Iloilo City 5000, Philippines
[2]Center for Natural Drug Discovery and Development (CND3), University of San Agustin, Gen. Luna Street, Iloilo City 5000, Philippines
[3]Research Analytics, Early Solutions Data & Translational Services, UCB Celltech, Slough, Berkshire, United Kingdom SL1 3WE
[4]Balik Scientist Program, Department of Science and Technology Philippine Council for Health Research and Development (DOST-PCHRD), Bicutan, Taguig City 1631, Philippines
[5]Department of Chemistry, College of Liberal Arts, Sciences, and Education, University of San Agustin, Iloilo City 5000, Philippines
[6]Department of Biology, College of Liberal Arts, Sciences, and Education, University of San Agustin, Iloilo City 5000, Philippines

*Author to whom correspondence should be addressed. ddalisay@usa.edu.ph

Table of Contents

Table S1. Genomic Islands in *S. tubbatahanensis* DSD3025^T genome 5

Table S2. Transposases and Integrases predicted in *S. tubbatahanensis* DSD3025^T genome.. 6

Table S3. Predicted core genes and known resistance model hits in *S. tubbatahanensis* DSD3025^T genome 7

Table S4. Antimicrobial resistance genes in *S. tubbatahanensis* DSD3025^T genome predicted by CARD 8

Table S5. Cultural characteristics of *S. tubbatahanensis* DSD3025^T incubated at 28°C for 14 days 9

Table S6. Enzymatic and biochemical analyses of *S. tubbatahanensis* DSD3025^T..................10

Table S7. Known and novel carbazole alkaloids from *S. tubbatahanensis* DSD3025^T in the negative mode 11

Table S8. Known and novel chlorinated compounds from *S. tubbatahanensis* DSD3025^{T}11 in the positive mode 11

Table S9. NMR Spectroscopic Data (600 MHz, $CDCl_3$) for **1** produced by *S. tubbatahanensis* DSD3025^T and from *S. diacarni* LHW51701^T..12

Table S10. Actinomycete secondary metabolites from tryptophan halogenase precursors and intermediates from rebeccamycin and pyrrolnitrin biosynthesis 13

Table S11. Non-halogenated Carbazole Alkaloids and Indolocarbazoles isolated from Actinomycetes...18

Table S12. MS-DIAL® parameters 22

Figure S1. Phylogenetic analysis of the 16S rRNA gene sequences of *S. tubbatahanensis* DSD3025^T and its closely related *Streptomyces* species based on (A) neighbor-joining and (B) maximum parsimony algorithms...25

Figure S2. Phylogenetic analysis of concatenated five house-keeping genes (*atpD, gyrB, rpoB, recA*, and *trpB*) of *S. tubbatahanensis* DSD3025^T and its closely related *Streptomyces* species based on (A) neighbor-joining and (B) maximum parsimony algorithms.. 25

Figure S3. Subsystem information of *S. tubbatahanensis* DSD3025^T genome annotated by the Rapid Annotation using Subsystem Technology (RAST v2). 26

Figure S4. Enzymatic activities of *Streptomyces* tubbatahaensis DSD3025^T as analyzed using the API® ZYM kit. 26

Figure S5. Major polar lipid profile of *S. tubbatahanensis* DSD3025^T. DPG, diphosphatidylglycerol; PE, phosphatidylethanolamine; APL, aminophospholipids; GL, glycolipids; PL, unidentified phospholipids; L, unidentified lipids. 27

Figure S6. BGC core structures detected in *S. tubbatahanensis* DSD3025^T genome as predicted by PRISM4 available at https://prism.adapsyn.com/. 28

Figure S7. Putative RiPPs and bacteriocin BGCs detected in *S. tubbatahanensis* DSD3025^T genome as predicted by BAGEL4 available at http://bagel4.molgenrug.nl/...............................30

Figure S8. Phylogenetic analysis of tryptophan halogenase of DSD3025^T and known regioselective flavin-dependent halogenases using maximum likelihood algorithms. 31

Figure S9. Detection of known and chlorinated carbazole alkaloids in *S. tubbatahanensis* DSD3025^T extract. 32

Figure S10. Mass ion peaks of DSD3025^T extract in the negative mode with chlorine and bromine isotope pattern. 33

Figure S11. Formula prediction of Chlocarbazomycin A **1**.. 34

Figure S12. Formula prediction of Chlocarbazomycin B **2a** or Chlocarbazomycin C **2b**. 35

Figure S13. MS/MS analysis of *m/z* 232.0521 [M+H]$^+$.. 36

Figure S14. Formula prediction of Chlocarbazomycin E **3**. 37

Figure S15. Formula prediction of Brocarbazomycin A **4**. 38

Figure S16. Formula prediction of Thiocarbazomycin A **5**. 39

Figure S17. Formula prediction of Thiocarbazomycin B **6**. 40

Figure S18. Reconstructed phylogenetic tree analysis of the 16S rRNA gene sequences of *S. tubbatahanensis* DSD3025^T and its closely related *Streptomyces* species based on maximum likelihood algorithm. 41

Figure S19. Molecular networking via MS-DIAL® showing the clustering of chlorinated compounds chlocarbazomycin A (**1**) (*m/z* 230.0376 [M-H]$^-$), chlocarbazomycin B **2a** or C **2b** (*m/z* 246.0314 [M-H]$^-$), and a new sulfur-containing carbazole alkaloid moiety (**7**) (*m/z* 287.0042 [M-H]$^-$). 42

Figure S20. Formula prediction of Compound **7**. 43

Figure S21. Formula prediction of chlorinated compound (*m/z* 232.0521 [M+H]$^+$) detected in the positive mode. 44

Figure S22. Formula prediction of chlorinated compound (*m/z* 317.1419 [M+H]$^+$) detected in the positive mode. 45

Figure S23. Formula prediction of chlorinated compound (*m/z* 457.0958 [M+H]$^+$) detected in the positive mode. 46

Figure S24. Formula prediction of chlorinated compound (*m/z* 517.1436 [M+H]$^+$) detected in the positive mode. 47

Figure S25. Formula prediction of chlorinated compound (*m/z* 517.1439 [M+H]$^+$) detected in the positive mode. 48

Figure S26. MS/MS analysis of chlorinated compound (*m/z* 317.1419 [M+H]$^+$) from DSD3025^T. 49

Figure S27. MS/MS analysis of chlorinated compound (*m/z* 457.0958 [M+H]$^+$) from DSD3025^T.. 50

Figure S28. MS/MS analysis of chlorinated compound (*m/z* 517.1436 [M+H]$^+$) from DSD3025^T. 51

Figure S29. MS/MS analysis of chlorinated compound (m/z 517.1439 [M+H]$^+$) from DSD3025^T. 52

Figure S30. Mass-Directed Purification of *S. tubbatahanensis* DSD3025^T Extract....................53
Figure S31. High-Resolution Mass Spectrometry Analysis of Collected Fraction DSD3025H1. 54
Figure S32. Formula prediction of the isolated fraction of DSD3025H1.. 55
Figure S33. ^{1}H NMR Spectrum of **1** (600 MHz, $CDCl_3$)....................56
Figure S34. ^{1}H NMR Spectrum of **1** (600 MHz, $CDCl_3$, expansion, aliphatic region)....................57
Figure S35. ^{1}H NMR Spectrum of **1** (600 MHz, $CDCl_3$, expansion, aromatic region). 58
Figure S36. ^{1}H-decoupled ^{13}C NMR Spectrum of **1** (150 MHz, $CDCl_3$). 59
Figure S37. ^{1}H-decoupled ^{13}C NMR Spectrum of **1** (150 MHz, $CDCl_3$, expansion). 60
Figure S38. ^{13}C DEPTQ135 NMR Spectrum of **1** (150 MHz, $CDCl_3$).. 61
Figure S39. ^{13}C DEPTQ135 NMR Spectrum of **1** (150 MHz, $CDCl_3$, expansion). 62
Figure S40. COSY NMR Spectrum of **1** (600 MHz, $CDCl_3$). 63
Figure S41. COSY NMR Spectrum of **1** (600 MHz, $CDCl_3$, expansion). 64
Figure S42. HSQC NMR Spectrum of **1** (600 MHz, $CDCl_3$). 65
Figure S43. HMBC NMR Spectrum of **1** (600 MHz, $CDCl_3$, J_{CH} = 6 Hz)....................66
Figure S44. HMBC NMR Spectrum of **1** (600 MHz, $CDCl_3$, expansion, J_{CH} = 6 Hz)....................67
Figure S45. HMBC NMR Spectrum of **1** (600 MHz, $CDCl_3$, expansion, J_{CH} = 6 Hz)....................68
Figure S46. NOESY NMR Spectrum of **1** (600 MHz, $CDCl_3$, mixing time = 750 ms)....................69
Figure S47. NOESY NMR Spectrum of **1** (600 MHz, $CDCl_3$, mixing time = 750 ms, expansion). 70
Figure S48. Distances measured between -NH/H1, -NH/H8, and H2/-OCH_3 using Chemdraw 3D (v21.0.0)....................71
Figure S49. Detection of Non-Halogenated Carbazole Alkaloids in *S. tubbatahanensis* DSD3025^T Extract....................72
Figure S50. Formula prediction of compound **8** (*m/z* 242.1178 $[M+H]^+$) detected in the positive mode....................73
Figure S51. MS/MS Analysis of Carbazomycin B **8**....................74
Figure S52. Formula prediction of mass ion peak detected in the positive mode at *m/z* 288.1234 $[M+H]^+$....................75
Figure S53. Formula prediction of mass ion peak detected in the positive mode at *m/z* 338.2113 $[M+H]^+$....................76

Supplemental Materials

Table S1. Genomic Islands in *S. tubbatahanensis* DSD3025^T genome

Genomic Island (GI)	Gene Start	Gene End	Length (bp)	GI Method
**1**	48805	53095	4290	SIGI-HMM
**2**	1818511	1831519	13008	SIGI-HMM
**3**	2746771	2752564	5793	SIGI-HMM
**4**	5276499	5280698	4199	SIGI-HMM
**5**	4014978	4050413	35435	IslandPath-DIMOB
**6**	4335630	4351140	15510	IslandPath-DIMOB
**7**	5627735	5669587	41852	IslandPath-DIMOB
**8**	6242590	6309408	66818	IslandPath-DIMOB
**9**	6396945	6406354	9409	IslandPath-DIMOB
**10**	7080343	7092136	11793	IslandPath-DIMOB
**11**	7732319	7753416	21097	IslandPath-DIMOB

Table S2. Transposases and Integrases predicted in *S. tubbatahanensis* DSD3025^{T} genome

Predicted Transposases and Integrases	Gene Start	Gene End	Length (bp)
**IS5 family transposase**	123058	123938	880
**IS5/IS1182 family transposase**	129589	129779	190
**transposase**	266660	266923	263
**IS701 family transposase**	938331	938534	203
**IS5 family transposase**	939594	940072	478
**IS5 family transposase**	939594	940072	478
**IS701 family transposase**	6295174	6295523	349
**IS701 family transposase**	6297787	6297980	193
**transposase**	6302100	6302251	151
**transposase**	6302100	6302251	151
**IS5/IS1182 family transposase**	6519128	6519232	104
**IS5 family transposase**	7090869	7091749	880
**IS5/IS1182 family transposase**	7719360	7719515	155
**transposase**	7734238	7734885	647
**transposase**	7734238	7734885	647
**site-specific integrase**	4025877	4026032	155
**site-specific integrase**	4031122	4031371	249
**site-specific integrase**	5627735	5628973	1238
**site-specific integrase**	6309405	6310595	1190
**site-specific integrase**	6309405	6310595	1190

Table S3. Predicted core genes and known resistance model hits in *S. tubbatahanensis* DSD3025^T genome

	***S. tubbatahanensis* DSD3025^T (1)**	***S. reniochalinae* LHW50302^T (2)**	***S. diacarni* LHW51701^T (3)**
**Total Genes**	6579	6757	6628
**Total BGCs**	29	32	33
**Known resistance**	40	40	40
**Core genes**	392	393	393
**Gene duplication**	32	30	30
**BGC proximity**	17	16	16
**Phylogeny / HGT**	178	175	176

Table S4. Antimicrobial resistance genes in *S. tubbatahanensis* DSD3025^{T} genome predicted by CARD v3.1.1 available at https://card.mcmaster.ca/analyze/rgi.

Antibiotic Resistance Ontology (ARO)	Drug class	Resistance Mechanism	Percent Identity of Matching Region*
novA	aminocoumarin antibiotic	antibiotic efflux	77.51
oleC	macrolide antibiotic	antibiotic efflux	76.09
mtrA	macrolide antibiotic; penam	antibiotic efflux	75.00
***Mycobacterium tuberculosis rpoB* mutants conferring resistance to rifampicin**	rifamycin antibiotic	antibiotic target alteration; antibiotic target replacement	75.00
Streptomyces lividans cmlR	phenicol antibiotic	antibiotic efflux	73.72
***Escherichia coli EF-Tu* mutants conferring resistance to Pulvomycin**	elfamycin antibiotic	antibiotic target alteration	73.48
tlrC	macrolide antibiotic; lincosamide antibiotic	antibiotic target protection	68.45
rphA	rifamycin antibiotic	antibiotic inactivation	60.74
Pseudomonas aeruginosa soxR	fluoroquinolone antibiotic; cephalosporin; glycylcycline; penam; tetracycline antibiotic; rifamycin antibiotic; phenicol antibiotic; disinfecting agents and antiseptics	antibiotic target alteration; antibiotic efflux	59.72
***Clostridioides difficile gyrB* conferring resistance to fluoroquinolones**	fluoroquinolone antibiotic	antibiotic target alteration	56.68
***Thermus thermophilus uL3* mutations conferring resistance to pleuromutilin antibiotics**	pleuromutilin antibiotic	antibiotic target alteration	56.19
vanJ	glycopeptide antibiotic	antibiotic target alteration	55.80
gimA	macrolide antibiotic	antibiotic inactivation	55.38
RbpA	rifamycin antibiotic	antibiotic target protection	54.63
myrA	macrolide antibiotic; lincosamide antibiotic	antibiotic target alteration	54.20
***Mycobacterium tuberculosis folC* with mutation conferring resistance to para-aminosalicylic acid**	Para-aminosalicylic acid	antibiotic target alteration	53.35
tetA	tetracycline antibiotic	antibiotic efflux	53.23
lmrC	lincosamide antibiotic	antibiotic target protection	52.80
oleB	macrolide antibiotic	antibiotic target protection	52.50
oleC	macrolide antibiotic	antibiotic efflux	51.13
abeS	macrolide antibiotic; aminocoumarin antibiotic	antibiotic efflux	50.98

Table S4. Antimicrobial resistance genes in *S. tubbatahanensis* DSD3025^T genome predicted by CARD v3.1.1 available at https://card.mcmaster.ca/analyze/rgi. (cont.)

Antibiotic Resistance Ontology (ARO)	Drug class	Resistance Mechanism	Percent Identity of Matching Region*
NmcR	carbapenem; cephalosporin; cephamycin; penam	antibiotic inactivation	50.82
***Escherichia coli soxR* with mutation conferring antibiotic resistance**	fluoroquinolone antibiotic; cephalosporin; glycylcycline; penam; tetracycline antibiotic; rifamycin antibiotic; phenicol antibiotic; disinfecting agents and antiseptics	antibiotic target alteration; antibiotic efflux	50.75
***Clostridioides difficile gyrA* conferring resistance to fluoroquinolones**	fluoroquinolone antibiotic	antibiotic target alteration	50.06
***Only ARO with more than 50% similarity were reported**			

Table S5. Cultural characteristics of *S. tubbatahanensis* DSD3025^T incubated at 28°C for 14 days

Medium	Substratum Mycelium	Aerial Mycelium	Diffusible Pigment
**MM1**	Pale orange yellow	White, +++	-
**MM3**	White	White, +	-
**MM11**	Light yellow	Yellowish white, ++	-
**YGC**	Pale orange yellow	White, +++	-
**NA**	Deep orange yellow	White, +++	-
**TSA**	Pale orange yellow	White, +++	-
**ISP2**	Pale orange yellow	White, +++	-
**ISP3**	Pale orange yellow	White, +++	-
**ISP4**	Light yellow	Yellowish white, +	-
**ISP9**	Pale orange yellow	White, ++	-
**+++, Abundant; ++, moderate; +, poor; -, no growth or no pigment**			

Table S6. Enzymatic and biochemical analyses of *S. tubbatahanensis* DSD3025^{T}

Enzyme	Result
**Negative control**	-
**Alkaline phosphatase**	+
**Esterase C4**	+
**Esterase/lipase C8**	+
**Lipase (C14)**	-
**Leucine arylamidase**	+
**Valine arylamidase**	-
**Cystine arylamidase**	-
**Trypsin**	-
**α-chymotrypsin**	-
**Acid phosphatase**	+
**Naphthol-AS-BI-phosphohydrolase**	+
**α-galactosidase**	-
**β-galactosidase**	-
**β-glucuronidase**	-
**α-glucosidase**	+
**β-glucosidase**	-
**N-acetyl-β-glucosaminidase**	-
**α-mannosidase**	-
**+, with enzymatic activity ; -, no enzymatic activity**	

Table S7. Known and novel carbazole alkaloids from *S. tubbatahanensis* DSD3025^T in the negative mode

Entry	t_R, min	*m/z*	Ion	Formula Predictor	Δ ppm	DBE	Fit Confidence	Putative Chemical ID
**1**	3.24	317.0164	$[M-H]^-$	$C_{15}H_{10}N_2O_2SCl$	3.8	11.5	99.90	Thiocarbazomycin B
**2**	3.38	253.0441	$[M-H]^-$	$C_{14}H_9N_2O_2S$	2.0	11.5	100.00	Thiocarbazomycin A
**3**	4.20	246.0324	$[M-H]^-$	$C_{13}H_9NO_2Cl$	0.8	9.5	100.00	Chlocarbazomycin B/C
**4**	4.20	287.0054	$[M-H]^-$	$C_{14}H_8N_2OSCl$	2.8	11.5	99.92	New compound
**5**	4.98	260.0490	$[M-H]^-$	$C_{14}H_{11}NO_2Cl$	4.6	9.5	90.90	Chlocarbazomycin E
**6**	5.24	230.0376	$[M-H]^-$	$C_{13}H_9NOCl$	0.8	9.5	100.00	Chlocarbazomycin A
**7**	5.39	273.9881	$[M-H]^-$	$C_{13}H_9NOBr$	4.7	9.5	100.00	Brocarbazomycin A

Table S8. Known and novel chlorinated compounds from *S. tubbatahanensis* DSD3025^T

Entry	t_R, min	*m/z*	Ion	Formula Predictor	Δ ppm	DBE	Fit Confidence	Putative Chemical ID
**1**	5.29	232.0521	$[M+H]^+$	$C_{13}H_{11}NOCl$	-3.4	8.5	94.80	Chlocarbazomycin A
**2**	6.68	317.1419	$[M+H]^+$	$C_{18}H_{22}N_2OCl$	-0.6	8.5	100.00	New compound
**3**	7.05	457.0958	$[M+H]^+$	$C_{26}H_{18}N_2O_4Cl$	0.7	18.5	99.82	New compound
**4**	7.23	517.1436	$[M+H]^+$	$C_{31}H_{22}N_4O_2Cl$	1.0	22.5	99.97	New compound
**5**	8.61	517.1426	$[M+H]^+$	$C_{31}H_{22}N_4O_2Cl$	1.5	22.5	99.96	New compound

Table S9. NMR Spectroscopic Data (600 MHz, CDCl3) for 1 produced by *S. tubbatahanensis* DSD3025[T] and from *S. diacarni* LHW51701[T] (Cheng *et. al*., *Chin J Chem.* **2021**, 39, 1188-1192).

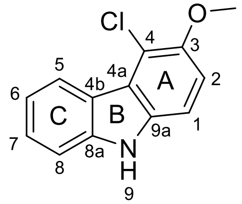

Position	1 Produced by *S. tubbatahanensis* DSD3025[T]					Chlocarbazomycin A[a]	
	δ_C, type	δ_H (m, *J* in Hz)	COSY	HMBC[b]	NOESY[c]	δ_C, type	δ_H (m, *J* in Hz)
**1**	109.0, CH	7.24, d (8.7)	H2	C2, C3, C4a, C9a	-NH	108.8, CH	7.26
**2**	112.8, CH	7.11, d (8.7)	H1	C1, C4, C3, C9a	-OCH_3	112.5, CH	7.11, d (8.6)
**3**	149.5, C					149.2, C	
**4**	117.4, C					117.1, C	
**4a**	122.3, C					122.0, C	
**4b**	123.0, C					122.7, C	
**5**	123.7, CH	8.61, d (8.0)	H6	C4a, C4b, C7, C8a		123.4, C	8.63, d (8.0)
**6**	119.8. CH	7.27, m	H5, H7	C4b, C5, C7, C8		119.5, CH	7.26
**7**	126.9, CH	7.43, m	H6, H8	C5, C6, C8a		126.6, CH	7.44-7.47
**8**	110.7, CH	7.39, d (8.0)	H7	C4b, C6, C7, C8a	-NH	110.5, CH	7.39, d (8.1)
**8a**	140.8, C					140.6, CH	
**9a**	135.7, C					135.4, C	
**-OCH_3**	58.3, CH_3	3.96, s		C2, C3	H2	58.1, CH_3	3.98, s
**-NH**		7.99, s			H1, H8		7.97, s

[a]Cheng *et. al*., *Chin J Chem.* **2021**, 39, 1188-1192
[b]HMBC correlations with J_{CH} = 6 Hz
[c]Mixing time = 750 m

Table S10. Actinomycete secondary metabolites from tryptophan halogenase precursors and intermediates from rebeccamycin and pyrrolnitrin biosynthesis

Entry	Compound	Chemical Formula	Exact Mass	$[M+H]^+$	$[M+Na]^+$	$[M+K]^+$	$[M+ACN+H]^+$	$[M-H]^-$	Remarks
**1**	7-chlorotryptophan (precursor)	$C_{11}H_{11}ClN_2O_2$	238.0509	239.0587	261.0407	277.0146	280.0853	237.0431	Not found
**2**	7-dechlorotryptophan (intermediate)	$C_{11}H_9ClN_2O_2$	236.0353	237.0431	259.0251	274.9990	278.0697	235.0275	Not found
**3**	7-chloro-indole-3-pyruvic acid	$C_{11}H_8ClNO_3$	237.0193	238.0271	260.0091	275.9830	279.0537	236.0115	Not found
**4**	3,4-bis(7-chloro-1H-indol-3-yl)-2-imino-5-oxohex-3-enedioic acid (intermediate)	$C_{22}H_{13}Cl_2N_3O_5$	469.0232	470.0310	492.0130	507.9869	511.0576	468.0154	Not found
**5**	3,4-bis(7-chloro-1H-indol-3-yl)-2,5-dioxohex-3-enedioic acid (intermediate)	$C_{22}H_{12}Cl_2N_2O_6$	470.0072	471.0150	492.9970	508.9709	512.0416	468.9994	Not found
**6**	11,11'-dichlorochromopyrrolic acid (intermediate)	$C_{22}H_{13}Cl_2N_3O_4$	453.0283	454.0361	476.0181	491.9920	495.0627	452.0205	Not found
**7**	Rebeccamycin	$C_{27}H_{21}Cl_2N_3O_7$	569.0757	570.0835	592.0655	608.0394	611.1101	568.0679	Not found

Table S10. Actinomycete secondary metabolites from tryptophan halogenase precursors and intermediates from rebeccamycin and pyrrolnitrin biosynthesis (cont)

Entry	Compound	Chemical Formula	Exact Mass	[M+H]$^+$	[M+Na]$^+$	[M+K]$^+$	[M+ACN +H]$^+$	[M-H]$^-$	Remarks
**8**	Rebeccamycin aglycone	$C_{20}H_{11}Cl_2N_3O_2$	393.0072	394.0150	415.9970	431.9709	435.0416	391.9994	Not found
**9**	9-methoxyrebeccamycin	$C_{28}H_{23}Cl_2N_3O_8$	599.0862	600.0940	622.0760	638.0499	641.1206	598.0784	Not found
**10**	9-methoxyrebeccamycin aglycone	$C_{21}H_{11}Cl_2N_3O_3$	423.0177	424.0255	446.0075	461.9814	465.0521	422.0099	Not found
**11**	Pyrrolnitrin	$C_{10}H_6Cl_2N_2O_2$	255.9806	256.9884	278.9704	294.9443	298.0150	254.9728	Not found
**12**	3-(3-chloro-2-nitrophenyl)-1H-pyrrole	$C_{10}H_7ClN_2O_2$	222.0196	223.0274	245.0094	260.9833	264.0540	221.0118	Not found
**13**	monodechloroaminopyrrolnitrin (Intermediate)	$C_{10}H_9ClN_2$	192.0454	193.0532	215.0352	231.0091	234.0798	191.0376	Not found
**14**	aminopyrrolnytrin (intermediate)	$C_{10}H_8Cl_2N_2$	226.0065	227.0143	248.9963	264.9702	268.0409	224.9987	Not found

Table S10. Actinomycete secondary metabolites from tryptophan halogenase precursors and intermediates from rebeccamycin and pyrrolnitrin biosynthesis (cont)

Entry	Compound	Chemical Formula	Exact Mass	$[M+H]^+$	$[M+Na]^+$	$[M+K]^+$	$[M+ACN+H]^+$	$[M-H]^-$	Remarks
**15**	lynamicin A	$C_{22}H_{15}Cl_2N_3O_2$	423.0541	424.0619	446.0439	462.0178	465.0885	422.0463	Not found
**16**	lynamicin B	$C_{22}H_{14}Cl_3N_3O_2$	457.0152	458.0230	480.0050	495.9789	499.0496	456.0074	Not found
**17**	lynamicin C	$C_{20}H_{11}Cl_4N_3$	432.9707	433.9785	455.9605	471.9344	475.0051	431.9629	Not found
**18**	lynamicin D	$C_{24}H_{17}Cl_2N_3O_4$	481.0596	482.0674	504.0494	520.0233	523.0940	480.0518	Not found
**19**	lynamicin E	$C_{24}H_{18}ClN_3O_4$	447.0986	448.1064	470.0884	486.0623	489.1330	446.0908	Not found
**20**	indimycin A	$C_{23}H_{19}Cl_2N_3$	407.0956	408.1034	430.0854	446.0593	449.1300	406.0878	Not found
**21**	indimycin B	$C_{24}H_{21}Cl_2N_3$	421.1113	422.1191	444.1011	460.0750	463.1457	420.1035	Not found
**22**	indimycin C	$C_{23}H_{19}Cl_2N_3$	407.0956	408.1034	430.0854	446.0593	449.1300	406.0878	Not found

Table S10. Actinomycete secondary metabolites from tryptophan halogenase precursors and intermediates from rebeccamycin and pyrrolnitrin biosynthesis (cont)

Entry	Compound	Chemical Formula	Exact Mass	$[M+H]^+$	$[M+Na]^+$	$[M+K]^+$	$[M+ACN+H]^+$	$[M-H]^-$	Remarks
**23**	indimycin D	$C_{22}H_{17}Cl_2N_3$	393.0800	394.0878	416.0698	432.0437	435.1144	392.0722	Not found
**24**	indimycin E	$C_{23}H_{19}Cl_2N_3$	407.0956	408.1034	430.0854	446.0593	449.1300	406.0878	Not found
**25**	spiroindimycin A	$C_{24}H_{15}Cl_2N_3O_4$	479.0440	480.0518	502.0338	518.0077	521.0784	478.0362	Not found
**26**	spiroindimycin B	$C_{23}H_{17}Cl_2N_3O_2$	437.0698	438.0776	460.0596	476.0335	479.1042	436.0620	Not found
**27**	spiroindimycin C	$C_{22}H_{15}Cl_2N_3O_2$	423.0541	424.0619	446.0439	462.0178	465.0885	422.0463	Not found
**28**	spiroindimycin D	$C_{25}H_{19}Cl_2N_3O_4$	495.0753	496.0831	518.0651	534.0390	537.1097	494.0675	Not found
**29**	spiroindimycin E	$C_{23}H_{18}ClN_3O_2$	403.1088	404.1166	426.0986	442.0725	445.1432	402.1010	Not found
**30**	spiroindimycin F	$C_{23}H_{18}ClN_3O_2$	403.1088	404.1166	426.0986	442.0725	445.1432	402.1010	Not found

Table S10. Actinomycete secondary metabolites from tryptophan halogenase precursors and intermediates from rebeccamycin and pyrrolnitrin biosynthesis (cont)

Entry	Compound	Chemical Formula	Exact Mass	$[M+H]^+$	$[M+Na]^+$	$[M+K]^+$	$[M+ACN+H]^+$	$[M-H]^-$	Remarks
**31**	chlorokynurenine	$C_{10}H_{11}ClN_2O_3$	242.0458	243.0536	265.0356	281.0095	284.0802	241.0380	Not found
**32**	chlororeductasporine	$C_{22}H_{19}ClN_3^+$	360.1262	361.134	383.116	399.0899	402.1606	359.1184	Not found
**33**	dichlororeductasporine	$C_{22}H_{18}Cl_2N_3^+$	394.0872	395.095	417.077	433.0509	436.1216	393.0794	Not found
**34**	chlorohydroxysporine	$C_{20}H_{12}ClN_3O_3$	377.0567	378.0645	400.0465	416.0204	419.0911	376.0489	Not found
**35**	dichlorohydroxysporine	$C_{20}H_{11}Cl_2N_3O_3$	411.0177	412.0255	434.0075	449.9814	453.0521	410.0099	Not found

Table S11. Non-halogenated Carbazole Alkaloids and Indolocarbazoles isolated from Actinomycetes.

Entry	Compound	Chemical Formula	Exact Mass	$[M+H]^+$	$[M+Na]^+$	$[M+K]^+$	$[M+ACN+H]^+$	$[M-H]^-$	Remarks
**1**	Epacarbazolin A	$C_{22}H_{27}NO_4$	369.1940	370.2018	392.1838	408.1577	411.2284	368.1862	Not found
**2**	Epacarbazolin B	$C_{23}H_{29}NO_4$	383.2097	384.2175	406.1995	422.1734	425.2441	382.2019	Not found
**3**	Staurosporine	$C_{28}H_{26}N_4O_3$	466.2005	467.2083	489.1903	505.1642	508.2349	465.1927	Not found
**4**	Antiostatin A1	$C_{20}H_{24}N_2O_2$	324.1838	325.1916	347.1736	363.1475	366.2182	323.1760	Not found
**5**	Antiostatin A2	$C_{21}H_{26}N_2O_2$	338.1995	339.2073	361.1893	377.1632	380.2339	337.1917	Not found
**6**	Antiostatin A3	$C_{22}H_{28}N_2O_2$	352.2151	353.2229	375.2049	391.1788	394.2495	351.2073	Not found
**7**	Antiostatin A4	$C_{22}H_{28}N_2O_3$	352.2151	353.2229	375.2049	391.1788	394.2495	351.2073	Not found
**8**	Antiostatin B2	$C_{24}H_{32}N_4O_3$	424.2474	425.2552	447.2372	463.2111	466.2818	423.2396	Not found
**9**	Antiostatin B3	$C_{25}H_{34}N_4O_3$	438.2631	439.2709	461.2529	477.2268	480.2975	437.2553	Not found
**10**	Antiostatin B4	$C_{25}H_{34}N_4O_3$	438.2631	439.2709	461.2529	477.2268	480.2975	437.2553	Not found
**11**	Antiostatin B5	$C_{26}H_{36}N_4O_3$	452.2787	453.2865	475.2685	491.2424	494.3131	451.2709	Not found

Table S11. Non-halogenated Carbazole Alkaloids and Indolocarbazoles isolated from Actinomycetes (cont.)

Entry	Compound	Chemical Formula	Exact Mass	$[M+H]^+$	$[M+Na]^+$	$[M+K]^+$	$[M+ACN+H]^+$	$[M-H]^-$	Remarks
**12**	Lavanduquinocin	$C_{26}H_{31}NO_3$	405.2304	406.2382	428.2202	444.1941	447.2648	404.2226	Not found
**13**	Neocarazostatin B	$C_{22}H_{27}NO_3$	353.1991	354.2069	376.1889	392.1628	395.2335	352.1913	Not found
**14**	Staurosporinone	$C_{20}H_{13}N_3O$	311.1059	312.1137	334.0957	350.0696	353.1403	310.0981	Not found
**15**	Carbazomycin A	$C_{16}H_{17}NO_2$	255.1259	256.1337	278.1157	294.0896	297.1603	254.1181	Not found
**16**	Carbazomycin B	$C_{15}H_{15}NO_2$	241.1103	242.1181	264.1001	280.0740	283.1447	240.1025	**Found**
**17**	Carbazomycin C	$C_{16}H_{17}NO_3$	271.1208	272.1286	294.1106	310.0845	313.1552	270.1130	Not found
**18**	Carbazomycin D	$C_{17}H_{19}NO_3$	285.1365	286.1443	308.1263	324.1002	327.1709	284.1287	Not found
**19**	Carbazomycin E	$C_{16}H_{15}NO_3$	269.1052	270.1130	292.0950	308.0689	311.1396	268.0974	Not found
**20**	Carbazomycin F	$C_{17}H_{17}NO_4$	299.1158	300.1236	322.1056	338.0795	341.1502	298.1080	Not found
**21**	Carbazomycin G	$C_{15}H_{15}NO_3$	257.1052	258.1130	280.0950	296.0689	299.1396	256.0974	Not found
**22**	Carbazomycin H	$C_{16}H_{17}NO_4$	287.1158	288.1236	310.1056	326.0795	329.1502	286.1080	**Found**

Table S11. Non-halogenated Carbazole Alkaloids and Indolocarbazoles isolated from Actinomycetes (cont.)

Entry	Compound	Chemical Formula	Exact Mass	$[M+H]^+$	$[M+Na]^+$	$[M+K]^+$	$[M+ACN+H]^+$	$[M-H]^-$	Remarks
**23**	Carazostatin	$C_{20}H_{25}NO$	295.1936	296.2014	318.1834	334.1573	337.2280	294.1858	Not found
**24**	Carbazomadurin A	$C_{22}H_{27}NO_3$	353.1991	354.2069	376.1889	392.1628	395.2335	352.1913	Not found
**25**	Carbazomadurin B	$C_{23}H_{29}NO_3$	367.2148	368.2226	390.2046	406.1785	409.2492	366.2070	Not found
**26**	Neocarazostatin A	$C_{22}H_{27}NO_4$	369.1940	370.2018	392.1838	408.1577	411.2284	368.1862	Not found
**27**	epoxy-neocarazostatin B	$C_{22}H_{27}NO_4$	369.1940	370.2018	392.1838	408.1577	411.2284	368.1862	Not found
**28**	neocarazostatin C	$C_{23}H_{29}NO_4$	383.2097	384.2175	406.1995	422.1734	425.2441	382.2019	Not found
**29**	carbazoquinocin A	$C_{19}H_{21}NO_2$	295.1572	296.1650	318.1470	334.1209	337.1916	294.1494	Not found
**30**	carbazoquinocin B	$C_{20}H_{23}NO_2$	309.1729	310.1807	332.1627	348.1366	351.2073	308.1651	Not found
**31**	carbazoquinocin C	$C_{20}H_{23}NO_2$	309.1729	310.1807	332.1627	348.1366	351.2073	308.1651	Not found
**32**	carbazoquinocin D	$C_{21}H_{25}NO_2$	323.1885	324.1963	346.1783	362.1522	365.2229	322.1807	Not found
**33**	carbazoquinocin E	$C_{21}H_{25}NO_2$	323.1885	324.1963	346.1783	362.1522	365.2229	322.1807	Not found

Table S11. Non-halogenated Carbazole Alkaloids and Indolocarbazoles isolated from Actinomycetes (cont.)

Entry	Compound	Chemical Formula	Exact Mass	$[M+H]^+$	$[M+Na]^+$	$[M+K]^+$	$[M+ACN+H]^+$	$[M-H]^-$	Remarks
**34**	carbazoquinocin F	$C_{22}H_{27}NO_2$	337.2042	338.2120	360.194	376.1679	379.2386	336.1964	**Found**
**35**	precarazostatin	$C_{17}H_{19}NO_3$	285.1365	286.1443	308.1263	324.1002	327.1709	284.1287	Not found
**36**	precarquinostatin	$C_{16}H_{15}NO_3$	269.1052	270.1130	292.0950	308.0689	311.1396	268.0974	Not found
**37**	1-(2-hydroxypropyl)-2-methyl-9H-carbazole-3,4-diol	$C_{16}H_{17}NO_3$	271.1208	272.1286	294.1106	310.0845	313.1552	270.1130	Not found
**38**	carquinostatin A	$C_{21}H_{23}NO_3$	337.1678	338.1756	360.1576	376.1315	379.2022	336.1600	Not found
**39**	carquinostatin B	$C_{21}H_{23}NO_4$	353.1627	354.1705	376.1525	392.1264	395.1971	352.1549	Not found
**40**	reductasporine	$C_{22}H_{20}N_3^+$	326.1652	327.173	349.155	365.1289	368.1996	325.1574	Not found
**41**	hydroxysporine	$C_{20}H_{13}N_3O_3$	343.0957	344.1035	366.0855	382.0594	385.1301	342.0879	Not found
**42**	hydroxypyrrolinone indolocarbazole	$C_{20}H_{13}N_3O_2$	327.1008	328.1086	350.0906	366.0645	369.1352	326.093	Not found
**43**	pyrrolinone indolocarbazole	$C_{20}H_{13}N_3O$	311.1059	312.1137	334.0957	350.0696	353.1403	310.0981	Not found
**44**	maleimide indolocarbazole	$C_{20}H_{11}N_3O_2$	325.0851	326.0929	348.0749	364.0488	367.1195	324.0773	Not found

Table S12. MS-DIAL® parameters

MS-DIAL® Parameter	Input Character
Version	MS-DIAL ver 4.90
**Project**	
Experimental File	ID MS type Start mz End mz 0 SCAN 100 1500 1 MSE 50 1500
MS1 Data Type	Centroid
MS2 Data Type	Centroid
Ion Mode	Negative
Target	Metabolomics
**Data Collection Parameters**	
Retention Time Begin	0.5
Retention Time End	11
Mass Range Begin	100
Mass Range End	1500
MS2 Mass Range Begin	50
MS2 Mass Range End	1500
**Centroid Parameters**	
MS1 Tolerance	0.05
MS2 Tolerance	0.05
Isotope Recognition	True
Maximum charged number	2
**Data Processing**	
Smoothing Method	LinearWeightedMoving Average
Smoothing Level	3
Minimum peak width	5
Minimum peak height	3000
**Peak Spotting Parameters**	

Table S12. MS-DIAL® parameters (cont.)

**MS-DIAL® Parameter**	**Input Character**
Mass slice width	0.1
Exclusion mass list (mass &tolerance)	False
**Deconvolution parameters**	
Sigma window value	1
MS2Dec amplitude cut off	400
Exclude after precursor	True
Keep isotope until 0.5	
Keep original precursor isotopes	False
**MSP file and MS/MS identification setting**	
MSP File	D:\0003-DSD3025\MSMS-Public-Neg-VS17.msp
Retention time tolerance	0.5
Accurate mass tolerance (MS1)	0.005
Accurate mass tolerance (MS2)	0.025
Identification score cut off	80
Using retention time for scoring	False
Using retention time for filtering	False
**Text file and Post Identification**	
Text File	
Retention time tolerance	0.1
Accurate mass tolerance	0.01
Identification score cut off	85
**Advanced Setting for Identification**	
Relative abundance cut off	0
**Adduct Ion Setting**	
[M-H]-	True

Table S12. MS-DIAL® parameters (cont.)

MS-DIAL® Parameter	Input Character
[M-H20-H]-	True
[M+FA-H]-	True
[2M-H]-	True
[2M+FA-H]-	True
**Alignment Parameter Setting**	
Reference File	D:\0003-DSD3025\20220722-MSe-Neg-DSD3025_001.abf
Retention time tolerance	0.05
MS1 Tolerance	0.015
Retention time factor	0.5
MS1 factor	0.5
Peak count filter	0
N% detected in at least one group	0
Remove feature based on peak height fold-change	True
Sample max / blank average	5
Sample average / blank average	5
Keep identified and annotated metabolites	False
Keep removable features and assign the tag for checking	False
Gap filling by compulsion	True
**Tracking of isotope labels**	
Tracking of isotopic labels	False
**Ion Mobility**	
Ion Mobility Data	False

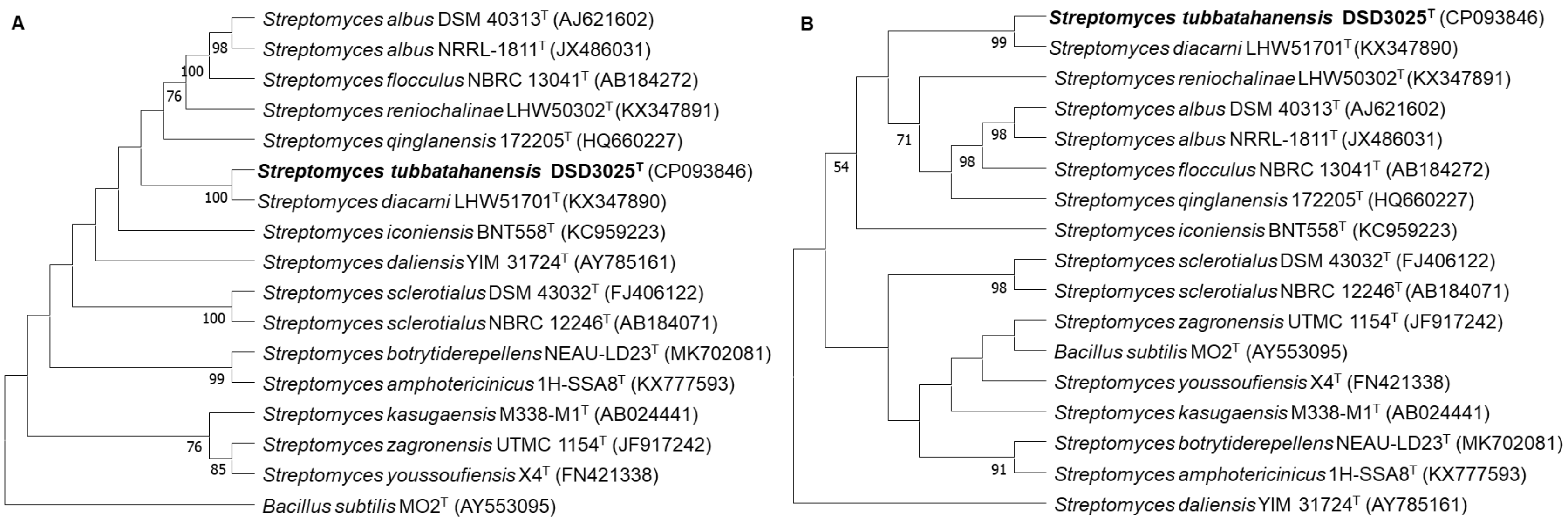

Figure S1. Phylogenetic analysis of the 16S rRNA gene sequences of *S. tubbatahanensis* DSD3025^T and its closely related Streptomyces species based on (A) neighbor-joining and (B) maximum parsimony algorithms. Numbers at nodes indicate levels of bootstrap support values of more than 50% based on 1,000 bootstrap replicates.

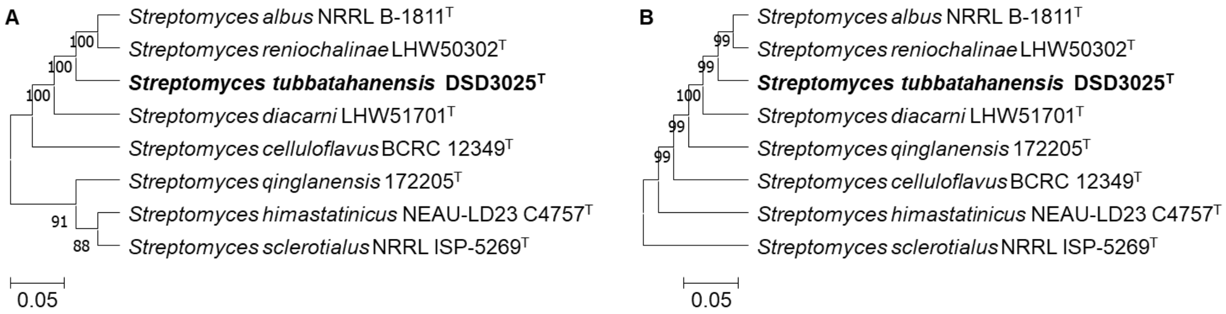

Figure S2. Phylogenetic analysis of concatenated five house-keeping genes (*atpD*, *gyrB, rpoB, recA*, and *trpB*) of *S. tubbatahanensis* DSD3025^T and its closely related Streptomyces species based on (A) neighbor-joining and (B) maximum parsimony algorithms. Numbers at nodes indicate levels of bootstrap support values of more than 50% based on 1,000 bootstrap replicates.

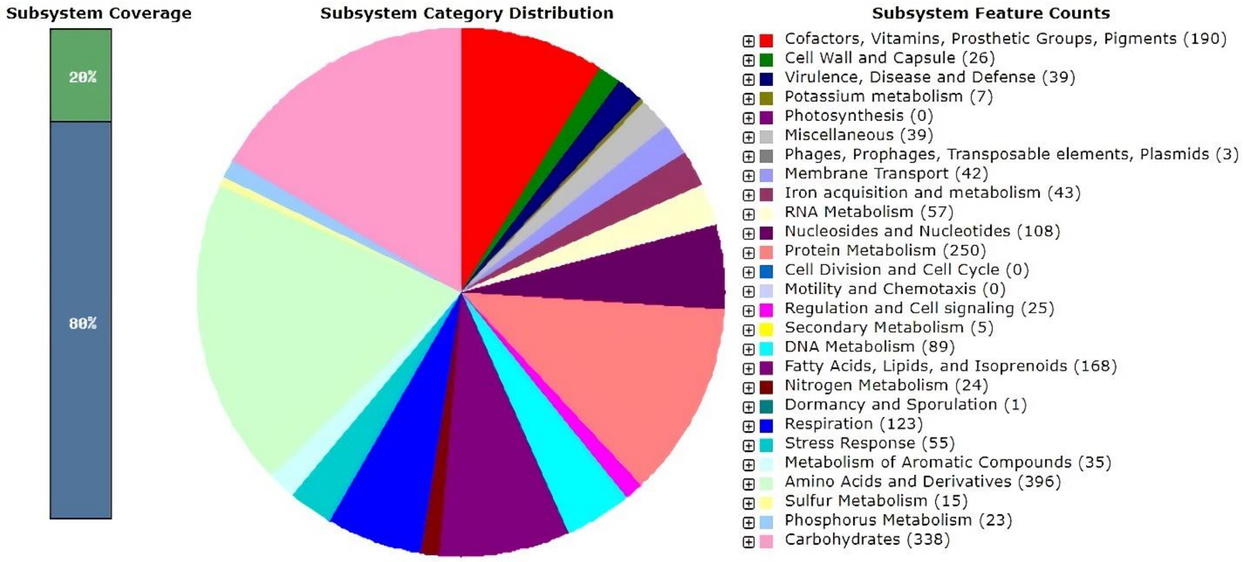

Figure S3. Subsystem information of *S. tubbatahanensis* DSD3025^{T} genome annotated by the Rapid Annotation using Subsystem Technology (RAST v2). The bar at the left side indicated that only 20% was included in the subsystem coverage. Majority of the genes were linked to amino acid and derivative metabolism (18.8%), carbohydrate metabolism (16.1%), followed by protein metabolism (11.9%).

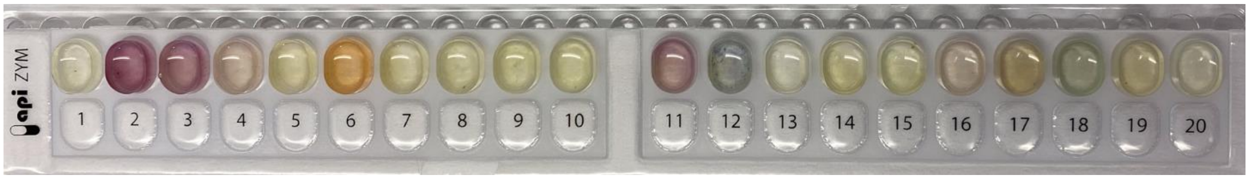

Figure S4. Enzymatic activities of *Streptomyces tubbatahaensis* DSD3025^{T} as analyzed using the API® ZYM kit. The API® ZYM strip: 1 (negative control), 2 (alkaline phosphatase), 3 (esterase C4), 4 (esterase/lipase C8),5 (lipase C14), 6 (leucine arylamidase), 7 (valine arylamidase), 8 (cystine arylamidase), 9 (trypsin), 10 (α-chymotrypsin), 11 (acid phosphatase), 12 (naphthol-AS-BI-phosphohydrolase), 13 (α-galactosidase), 14 (β-galactosidase), 15 (β-glucuronidase), 16 (α-glucosidase), 17 (β-glucosidase), 18 (N-acetyl-β-glucosaminidase), 19 (α-mannosidase), and 20 (α-fucosidase).

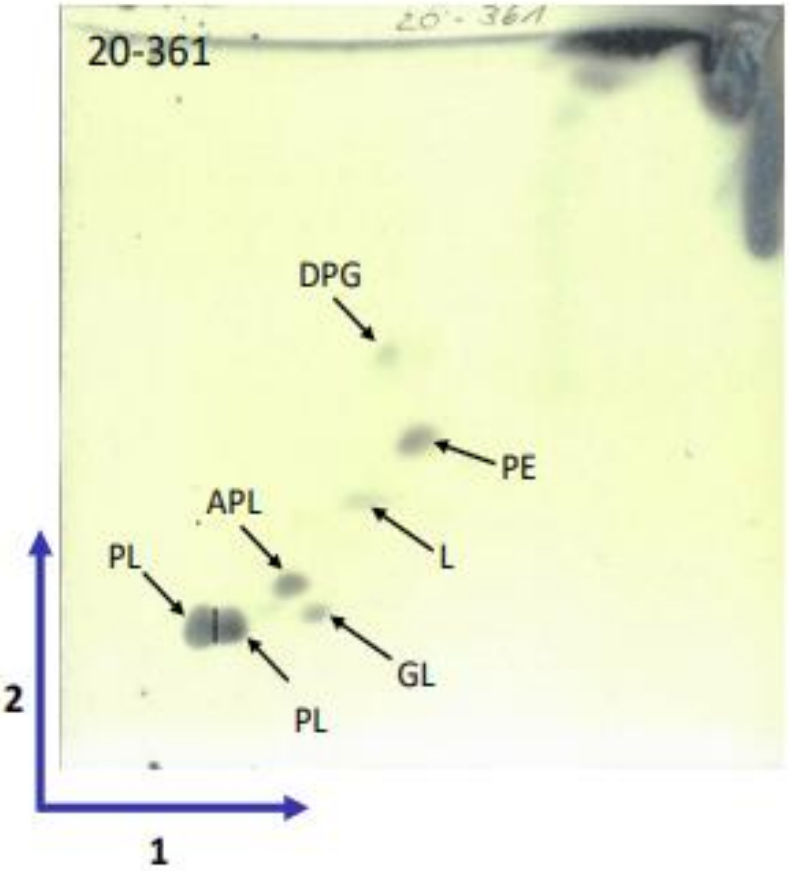

Figure S5. Major polar lipid profile of *S. tubbatahanensis* DSD3025^{T}. DPG, diphosphatidylglycerol; PE, phosphatidylethanolamine; APL, aminophospholipids; GL, glycolipids; PL, unidentified phospholipids; L, unidentified lipids.

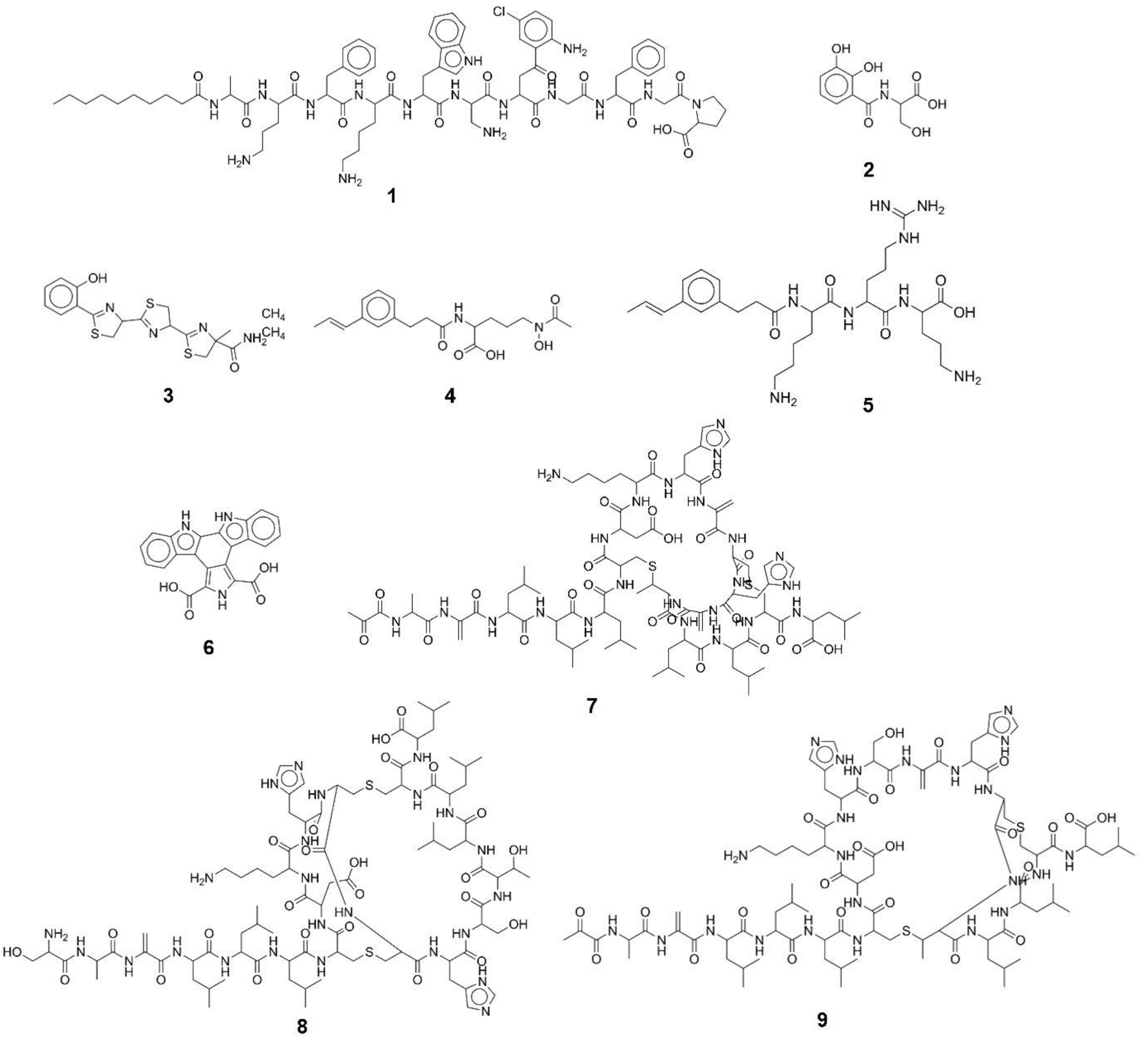

Figure S6. BGC core structures detected in *S. tubbatahanensis* DSD3025^T genome as predicted by PRISM4 available at https://prism.adapsyn.com/.

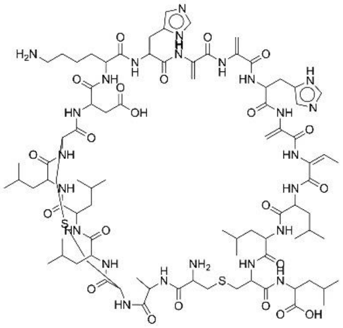

7

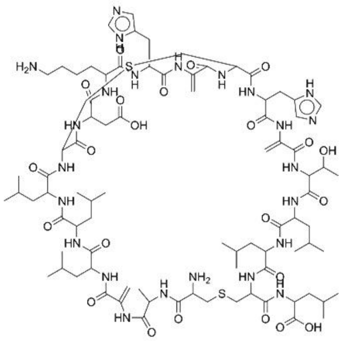

8

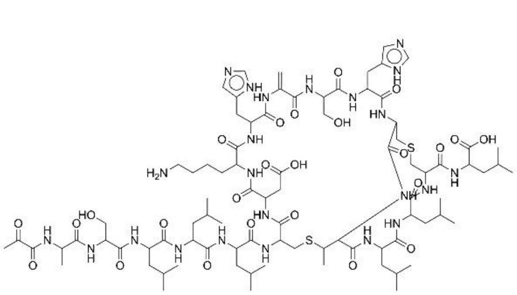

9

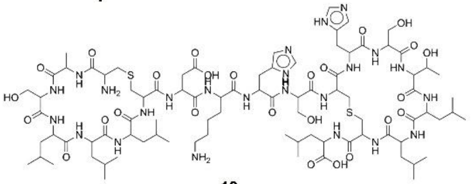

10

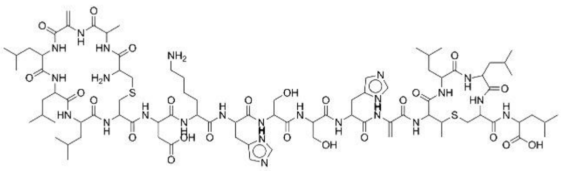

11

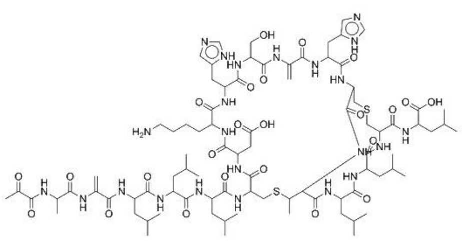

12

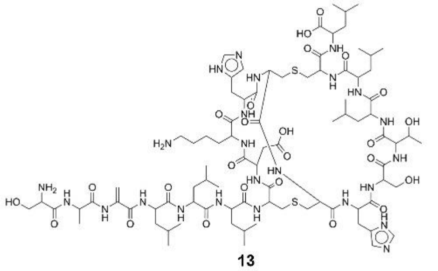

13

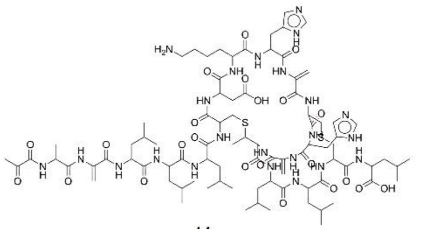

14

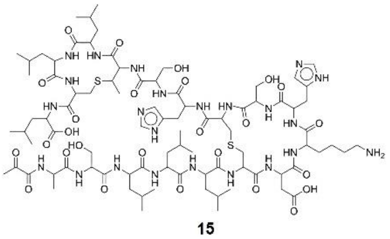

15

Figure S6 (cont.). BGC core structures detected in *S. tubbatahanensis* DSD3025^{T} genome as predicted by PRISM4 available at https://prism.adapsyn.com/.

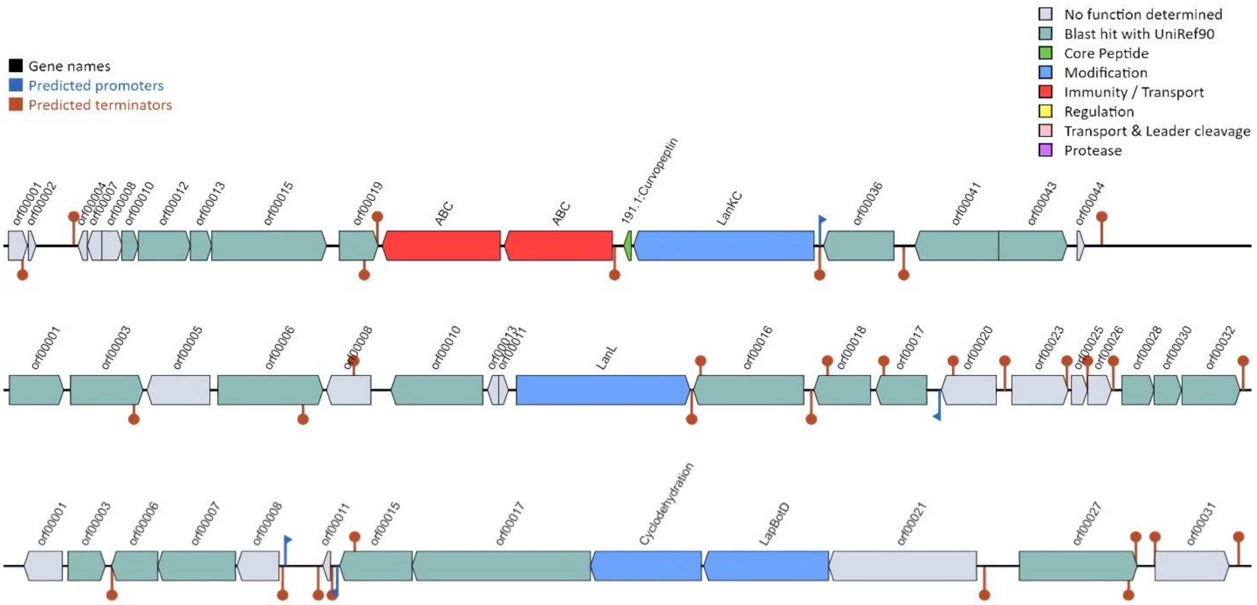

Figure S7. Putative RiPPs and bacteriocin BGCs detected in *S. tubbatahanensis* DSD3025^{T} genome as predicted by BAGEL4 available at http://bagel4.molgenrug.nl/.

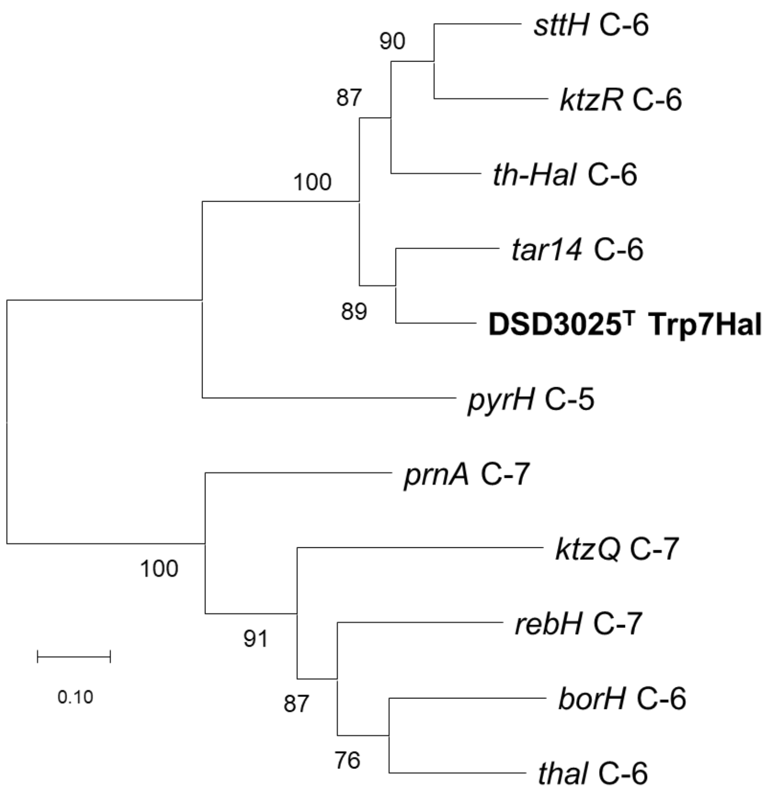

Figure S8. Phylogenetic analysis of tryptophan halogenase of DSD3025^{T} and known regioselective flavin-dependent halogenases using maximum likelihood algorithms.

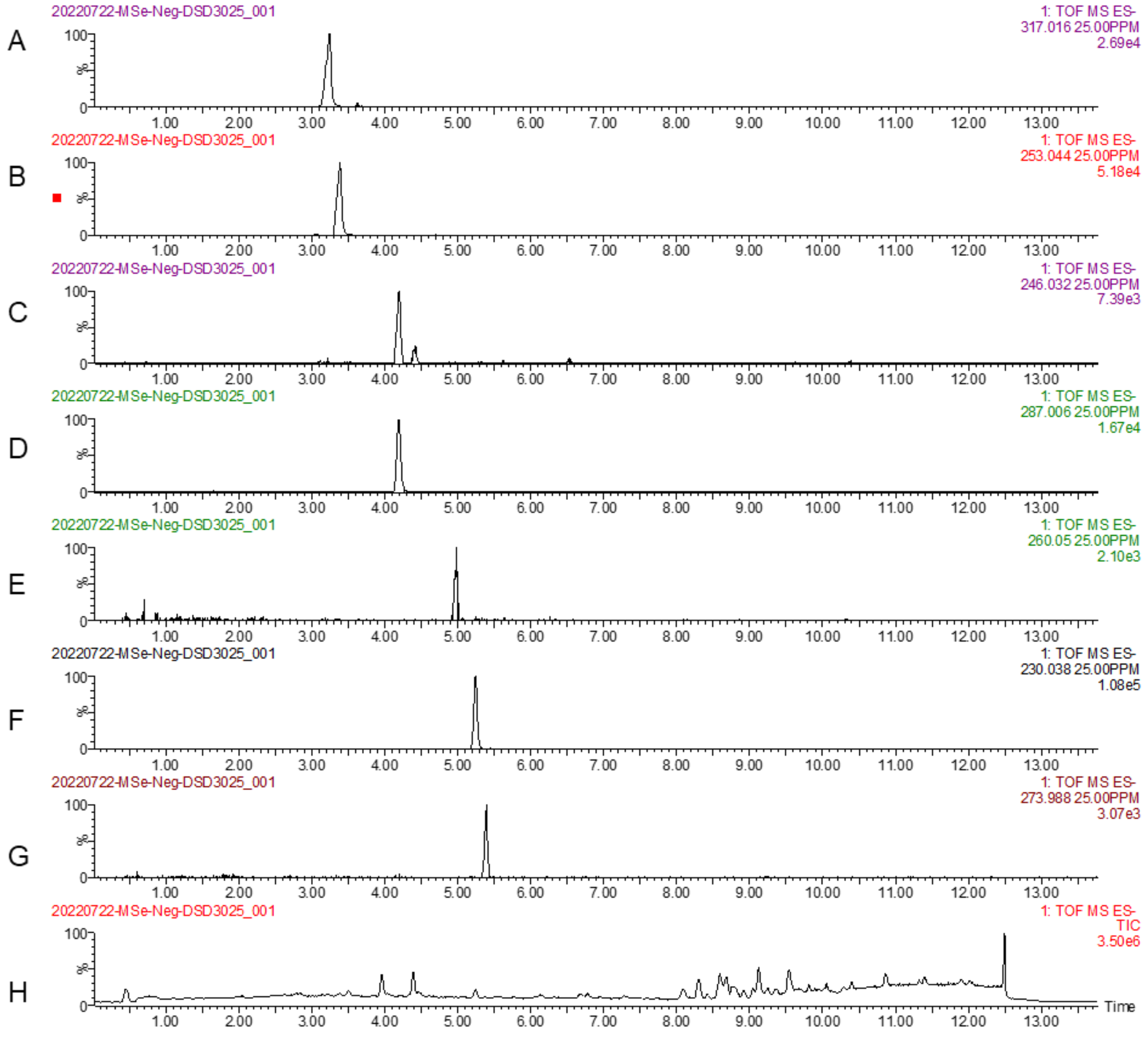

Figure S9. Detection of known and chlorinated carbazole alkaloids in *S. tubbatahanensis* DSD3025^T extract. Extracted Ion Chromatogram (EIC) in the negative mode of (A) thiocarbazomycin B, (B) thiocarbazomycin A, (C) chlocarbazomycin B or C, (D) new compound **7**, (E) chlocarbazomycin E, (F) chlocarbazomycin A, and (G) brocarbazomycin A. (H) Total Ion Chromatogram (TIC) of DSD3025^T in the negative mode.

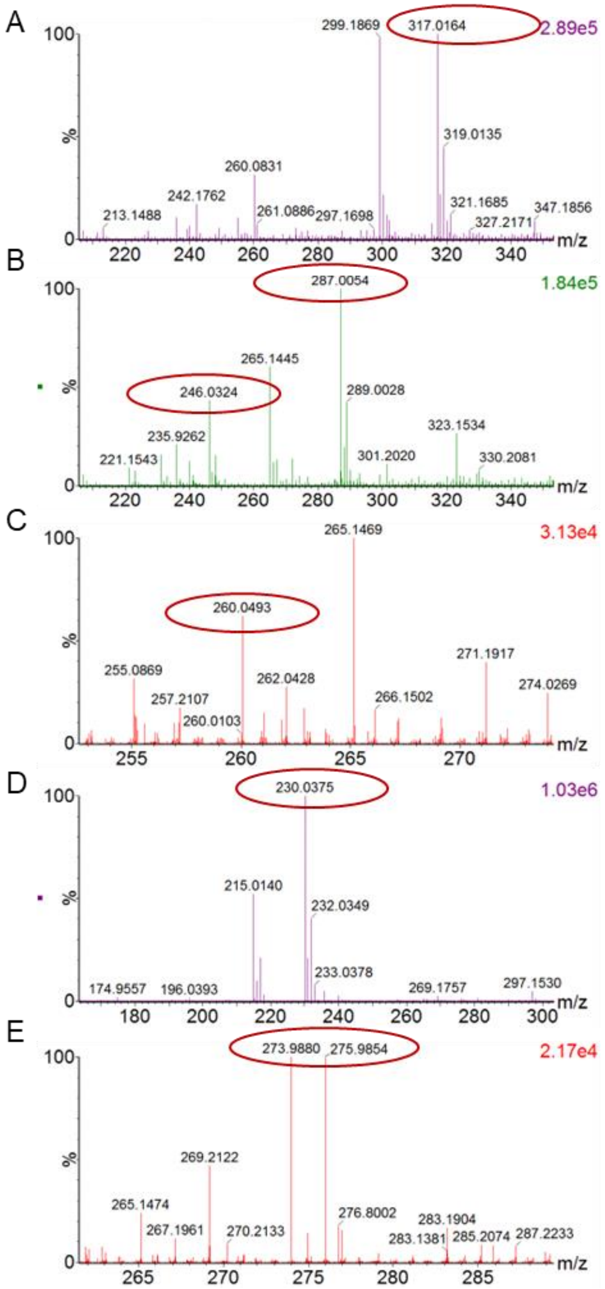

Figure S10. Mass ion peaks of DSD3025^{T} extract in the negative mode with chlorine and bromine isotope pattern. Mass ion peaks at (A) *m/z* 317.0164 $[M-H]^-$; (B) *m/z* 246.0324 $[M-H]^-$, *m/z* 287.0054 $[M-H]^-$; (C) *m/z* 260.0493 $[M-H]^-$; and *m/z* 230.0375 $[M-H]^-$ showed 3:1 isotope pattern of chlorine while mass ion peak at *m/z* 273.9880 $[M-H]^-$ showed 1:1 isotope pattern of bromine.

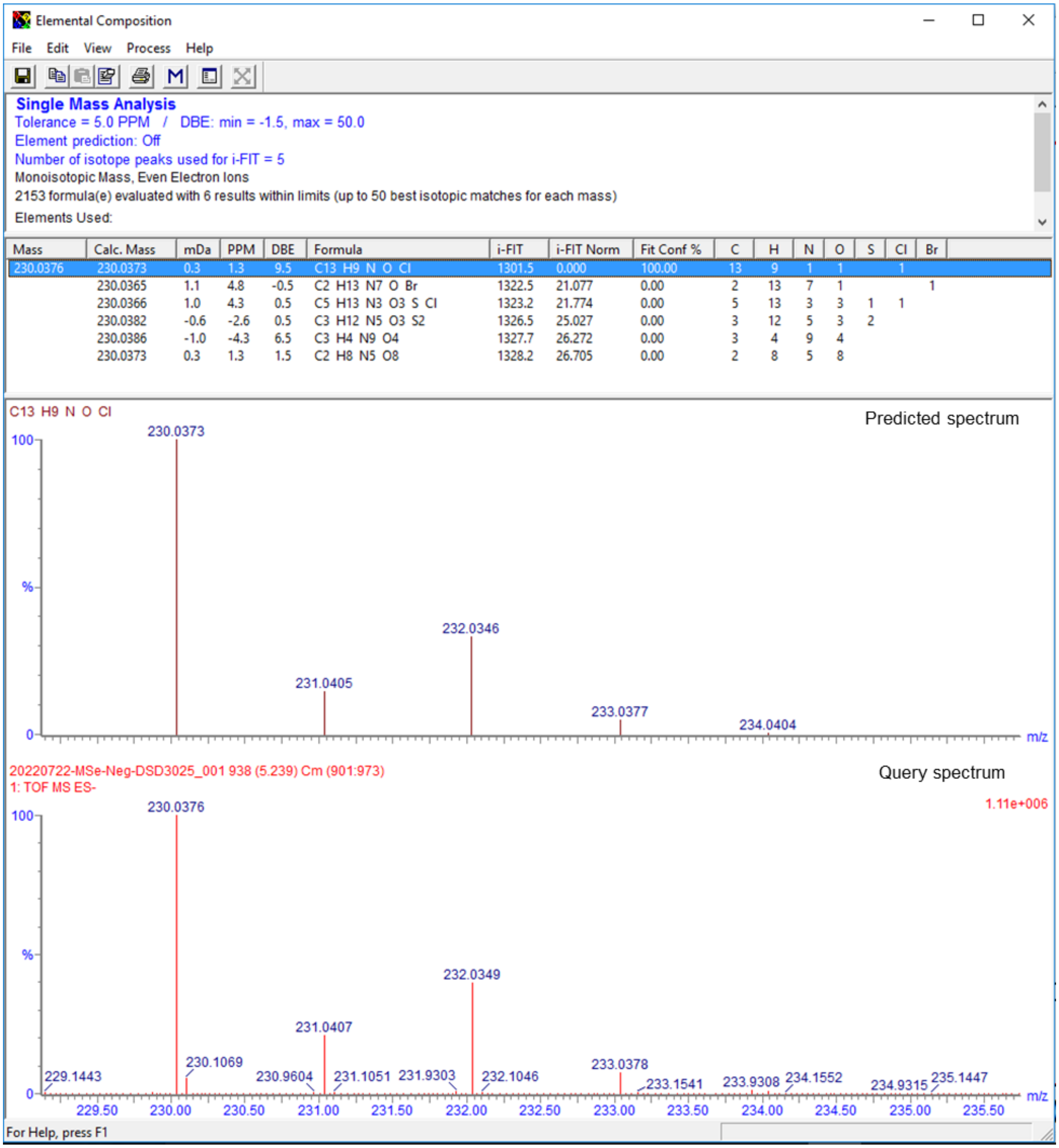

Mass	Calc. Mass	mDa	PPM	DBE	Formula	i-FIT	i-FIT Norm	Fit Conf %	C	H	N	O	S	Cl	Br
230.0376	230.0373	0.3	1.3	9.5	C13 H9 N O Cl	1301.5	0.000	100.00	13	9	1	1		1	
	230.0365	1.1	4.8	-0.5	C2 H13 N7 O Br	1322.5	21.077	0.00	2	13	7	1			1
	230.0366	1.0	4.3	0.5	C5 H13 N3 O3 S Cl	1323.2	21.774	0.00	5	13	3	3	1	1	
	230.0382	-0.6	-2.6	0.5	C3 H12 N5 O3 S2	1326.5	25.027	0.00	3	12	5	3	2		
	230.0386	-1.0	-4.3	6.5	C3 H4 N9 O4	1327.7	26.272	0.00	3	4	9	4			
	230.0373	0.3	1.3	1.5	C2 H8 N5 O8	1328.2	26.705	0.00	2	8	5	8			

Figure S11. Formula prediction of Chlocarbazomycin A **1**. Chemical formula was accurately predicted (<5.0 ppm) based on the measured mass, % fit confidence, and isotopic model. The mass ion peak at *m/z* 230.0376 $[M-H]^-$ was identified as chlocarbazomycin A with a neutral chemical formula of $C_{13}H_{10}NOCl$ and double bond equivalent of 9.0.

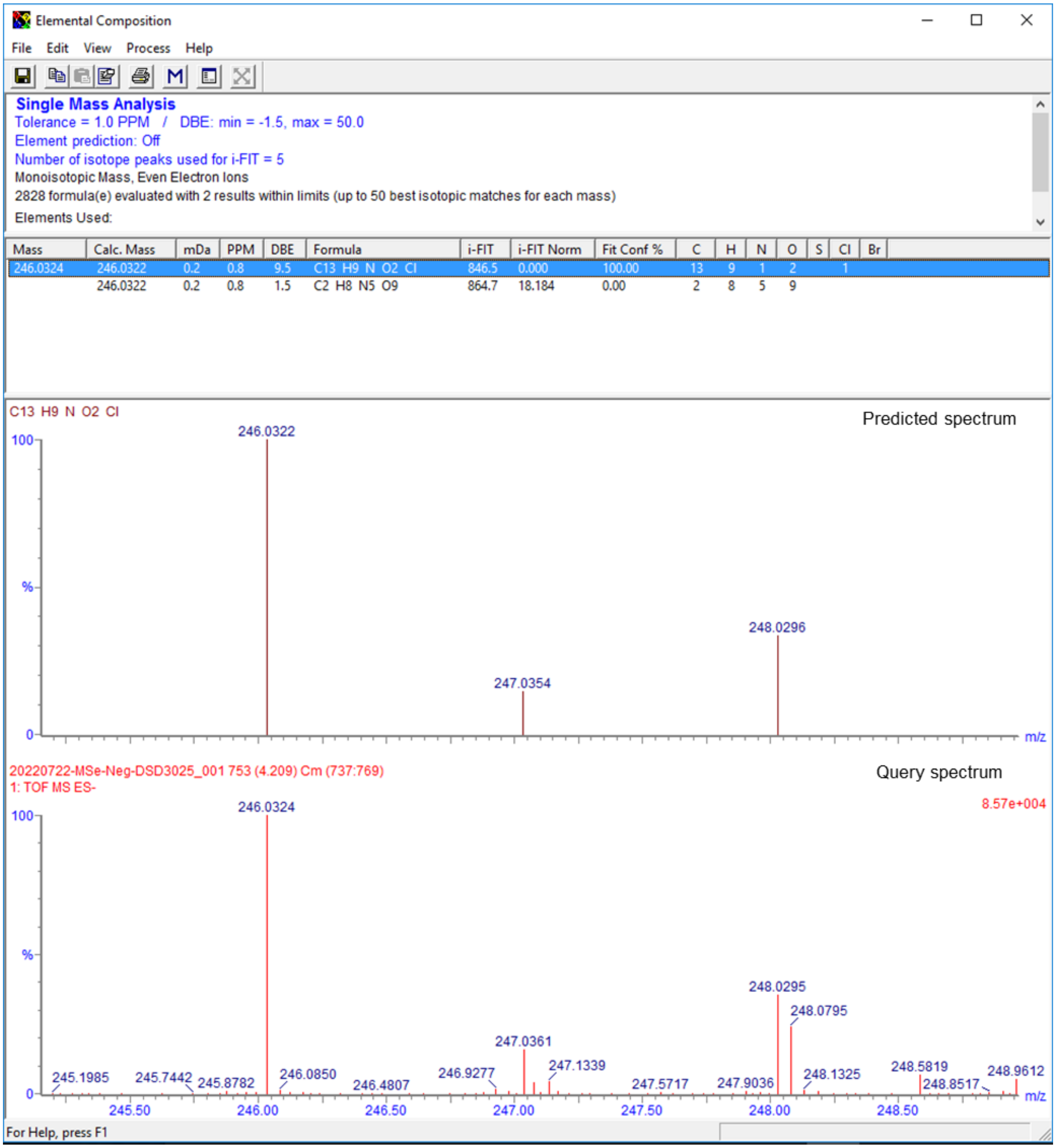

Figure S12. Formula prediction of Chlocarbazomycin B **2a** or Chlocarbazomycin C **2b**. Chemical formula was accurately predicted (<5.0 ppm) based on the measured mass, % fit confidence, and isotopic model. The mass ion peak at *m/z* 246.0324 [M-H]$^-$ was identified as chlocarbazomycin B or C with a neutral chemical formula of $C_{13}H_{10}NO_2Cl$ and double bond equivalent of 9.0.

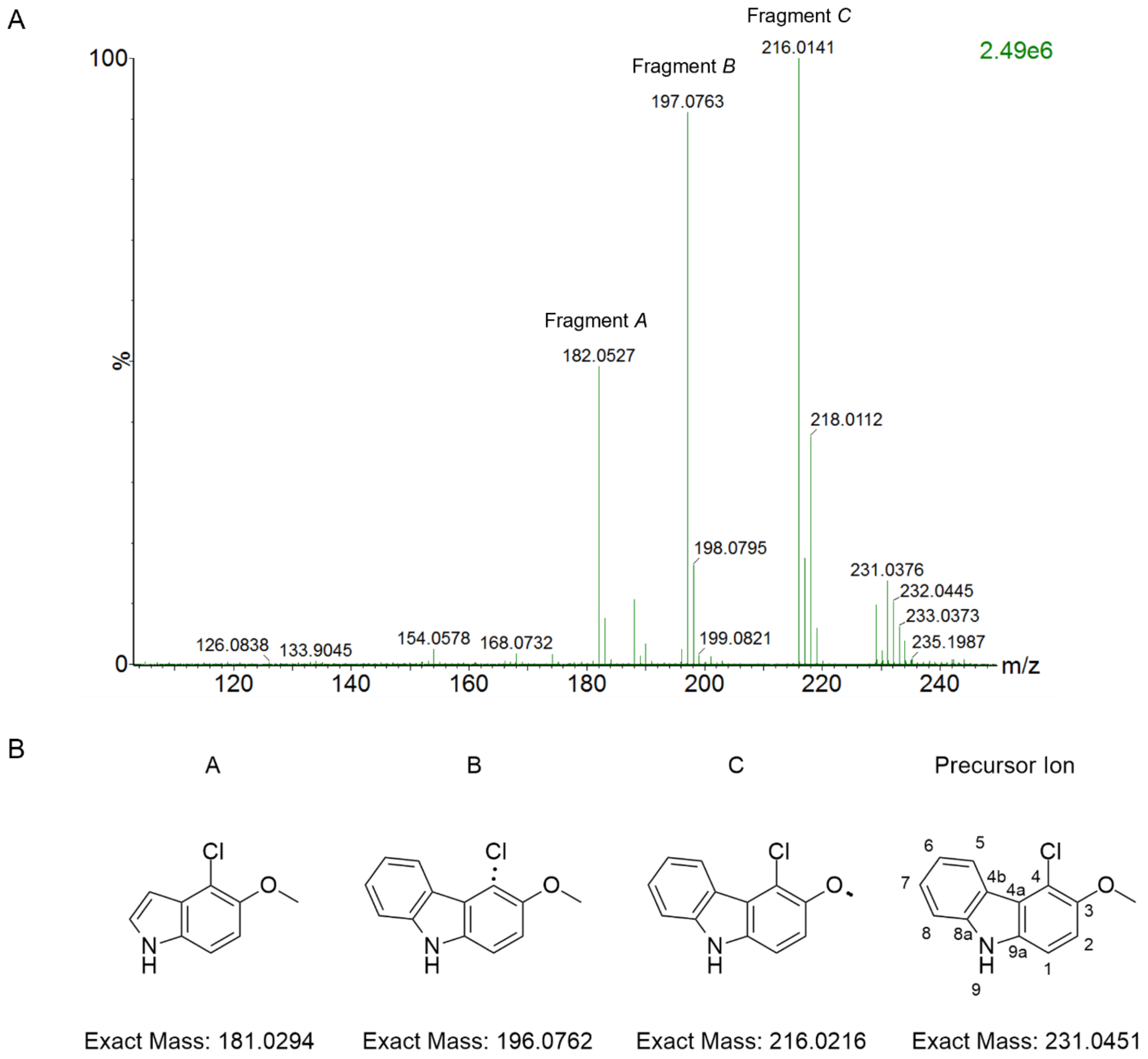

Figure S13. MS/MS analysis of *m/z* 232.0521 [M+H]$^+$. The mass ion peak at *m/z* 230.0376 [M-H]$^-$ detected in the negative mode has a corresponding mass ion peak in the positive mode at *m/z* 232.0521 [M+H]$^+$ at the same retention time. The MS/MS data was acquired in the positive mode since only one fragment peak was obtained in the negative mode. The experimental fragment peaks of *m/z* 232.0521 [M+H]$^+$ matched the MS/MS of known compound chlocarbazomycin A **1**.

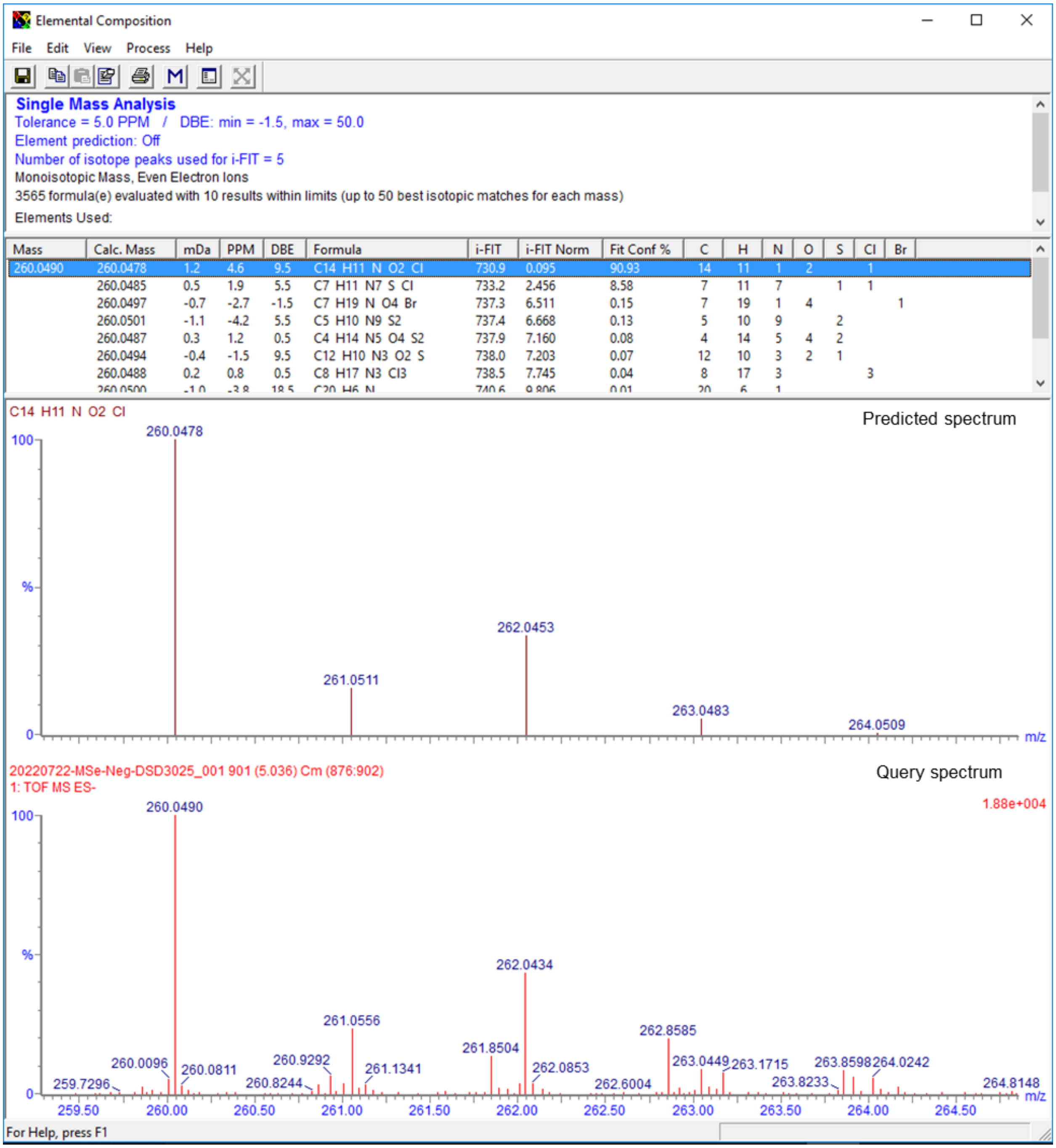

Figure S14. Formula prediction of Chlocarbazomycin E **3**. Chemical formula was accurately predicted (<5.0 ppm) based on the measured mass, % fit confidence, and isotopic model. The mass ion peak at *m/z* 260.0490 $[M\text{-}H]^-$ was identified as chlocarbazomycin E with a neutral chemical formula of $C_{14}H_{12}NO_2Cl$ and double bond equivalent of 9.0.

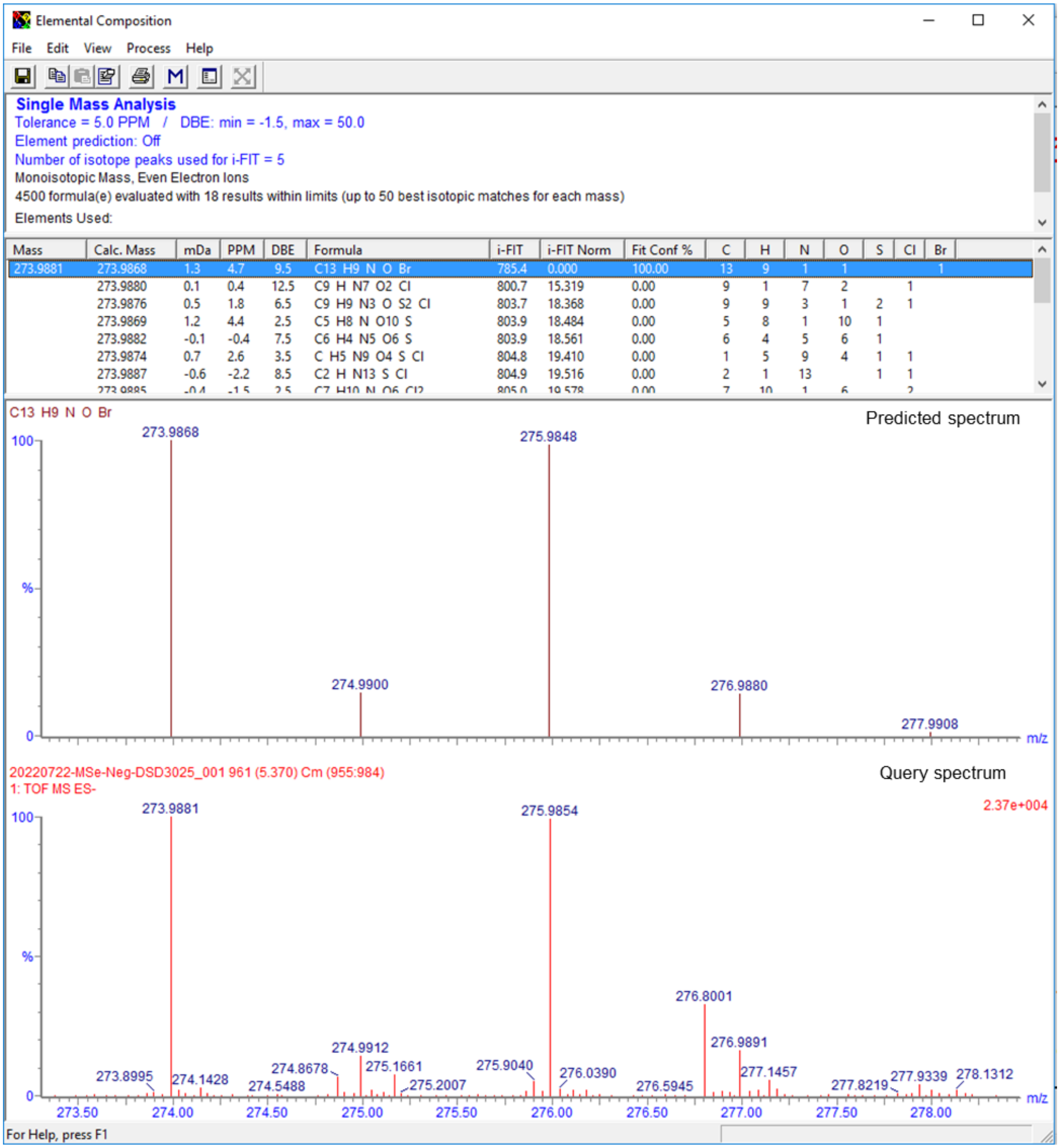

Figure S15. Formula prediction of Brocarbazomycin A **4**. Chemical formula was accurately predicted (<5.0 ppm) based on the measured mass, % fit confidence, and isotopic model. The mass ion peak at *m/z* 273.9881 $[M-H]^-$ was identified as brocarbazomycin A with a neutral chemical formula of $C_{13}H_{10}NOBr$ and double bond equivalent of 9.0.

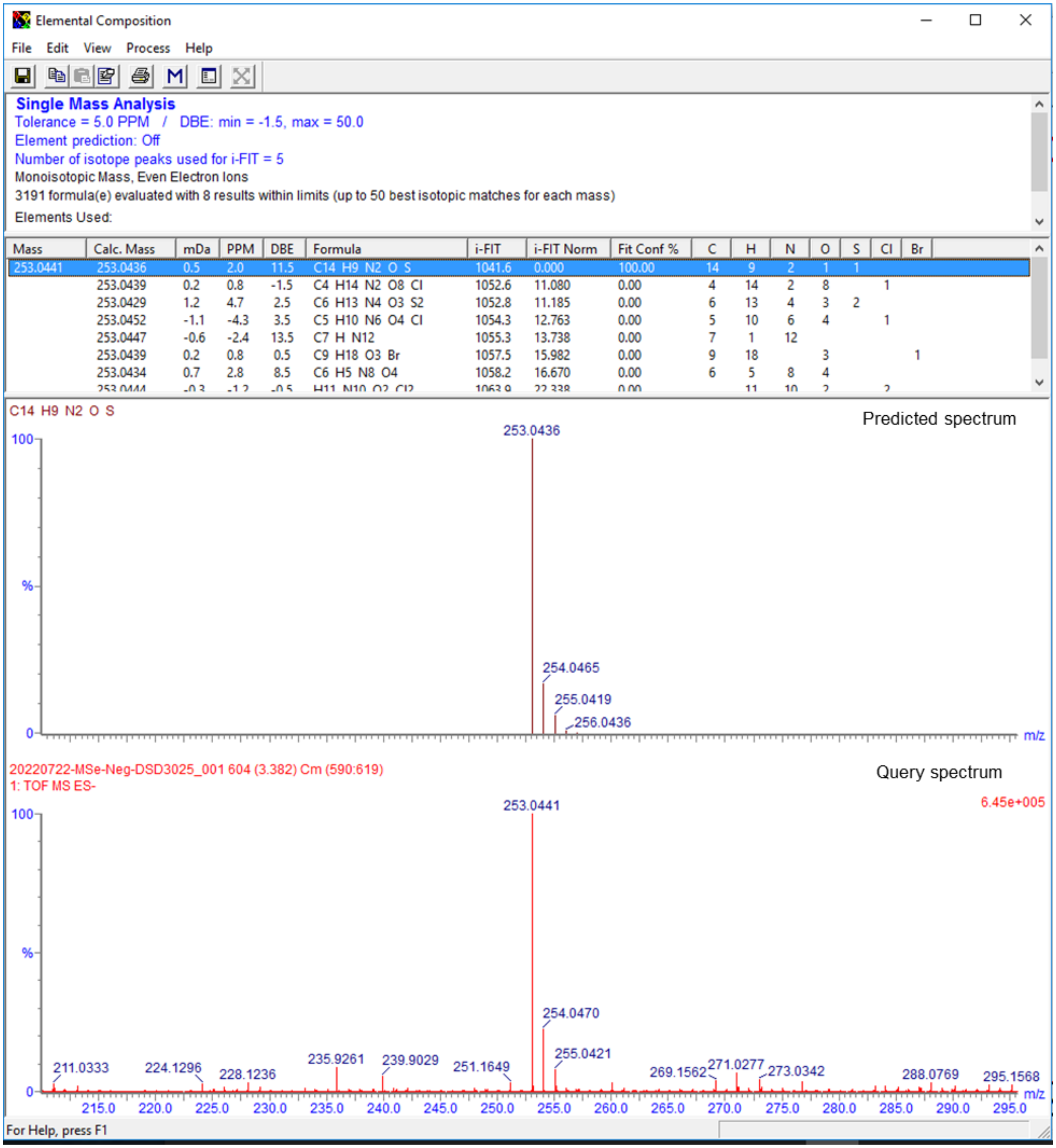

Figure S16. Formula prediction of Thiocarbazomycin A **5**. Chemical formula was accurately predicted (<5.0 ppm) based on the measured mass, % fit confidence, and isotopic model. The mass ion peak at *m/z* 253.0441 [M-H]$^-$ was identified as thiocarbazomycin A with a neutral chemical formula of $C_{14}H_{10}N_2OS$ and double bond equivalent of 11.0.

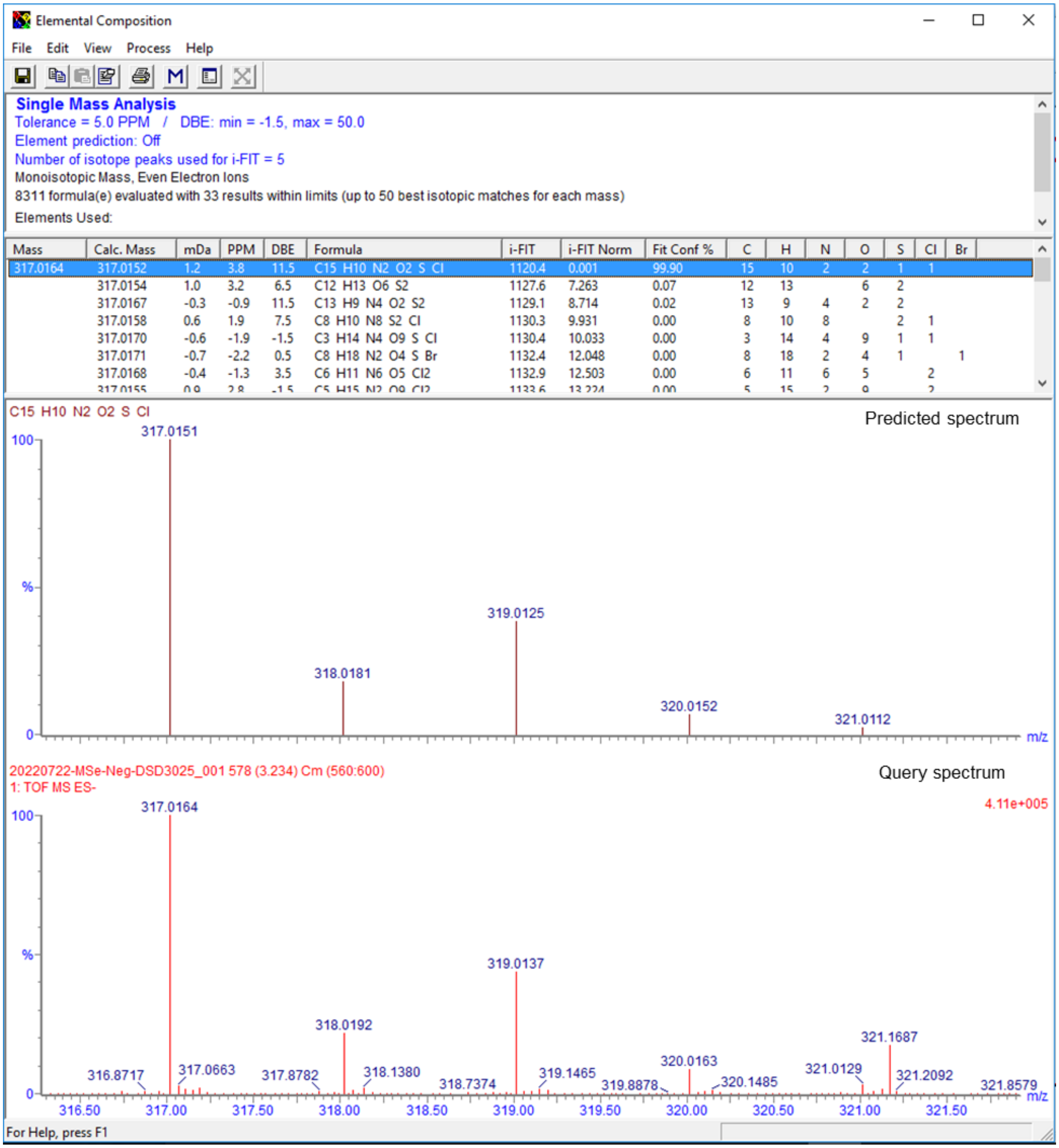

Figure S17. Formula prediction of Thiocarbazomycin B **6**. Chemical formula was accurately predicted (<5.0 ppm) based on the measured mass, % fit confidence, and isotopic model. The mass ion peak at *m/z* 317.0164 [M-H]$^-$ was identified as thiocarbazomycin B with a neutral chemical formula of $C_{15}H_{11}N_2O_2SCl$ and double bond equivalent of 11.0.

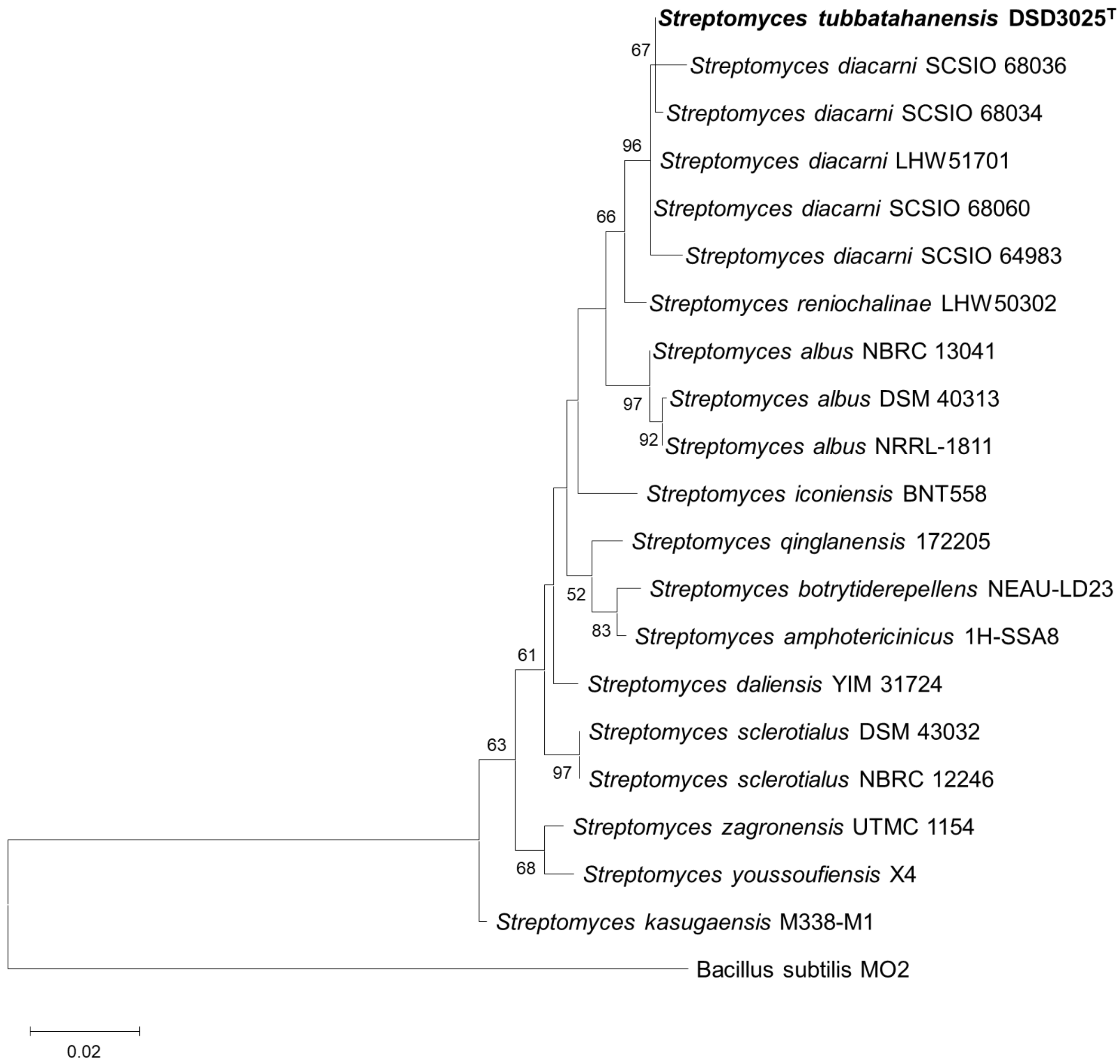

Figure S18. Reconstructed phylogenetic tree analysis of the 16S rRNA gene sequences of *S. tubbatahanensis* DSD3025^T and its closely related *Streptomyces* species based on maximum likelihood algorithm. Numbers at nodes indicate levels of bootstrap support values of more than 50% based on neighbor-joining analysis of 1,000 bootstrap replicates.

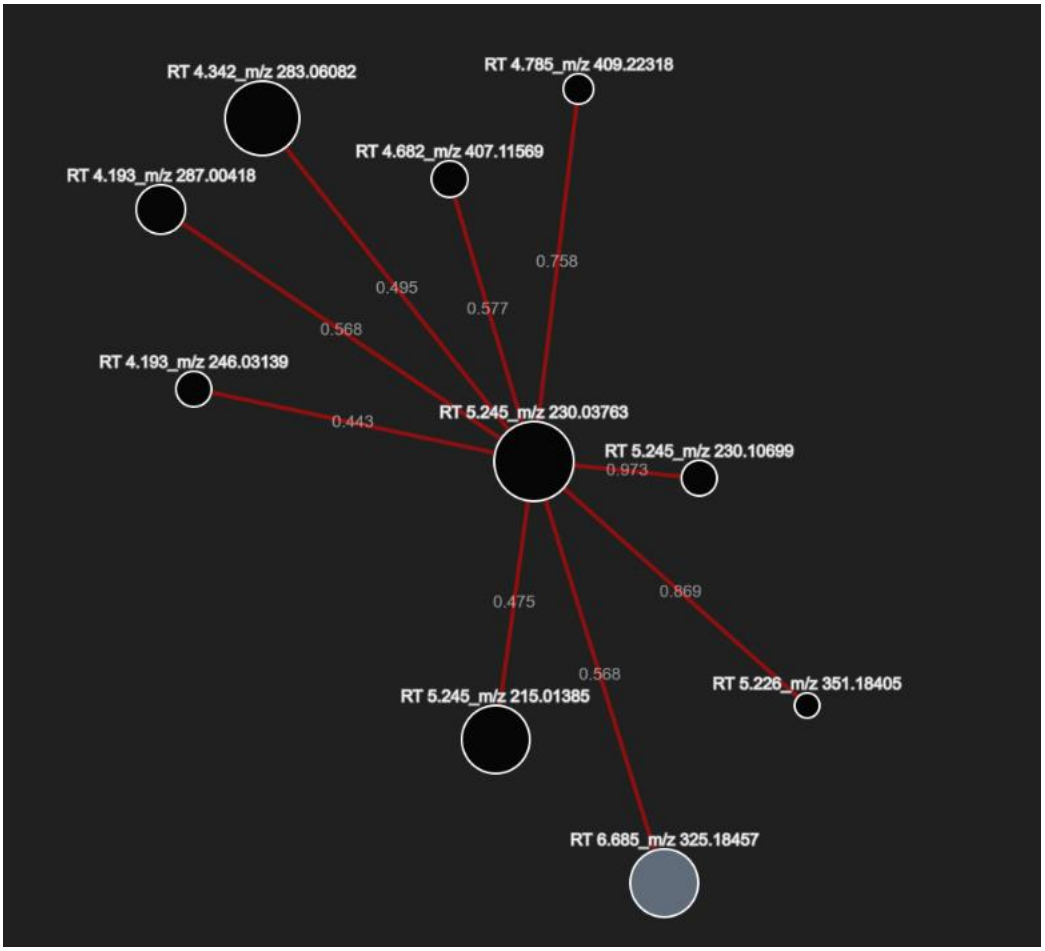

Figure S19. Molecular networking via MS-DIAL® showing the clustering of chlorinated compounds chlocarbazomycin A (**1**) (*m/z* 230.0376 $[M-H]^-$), chlocarbazomycin B **2a** or C **2b** (*m/z* 246.0314 $[M-H]^-$), and a new sulfur-containing carbazole alkaloid moiety (**7**) (*m/z* 287.0042 $[M-H]^-$). Peaks (nodes) were clustered based on a minimum of 40% similarity, MS/MS tolerance of 0.05 Da, and retention time tolerance of 1.5 min. Data was visualized using Mozilla Firefox html viewer.

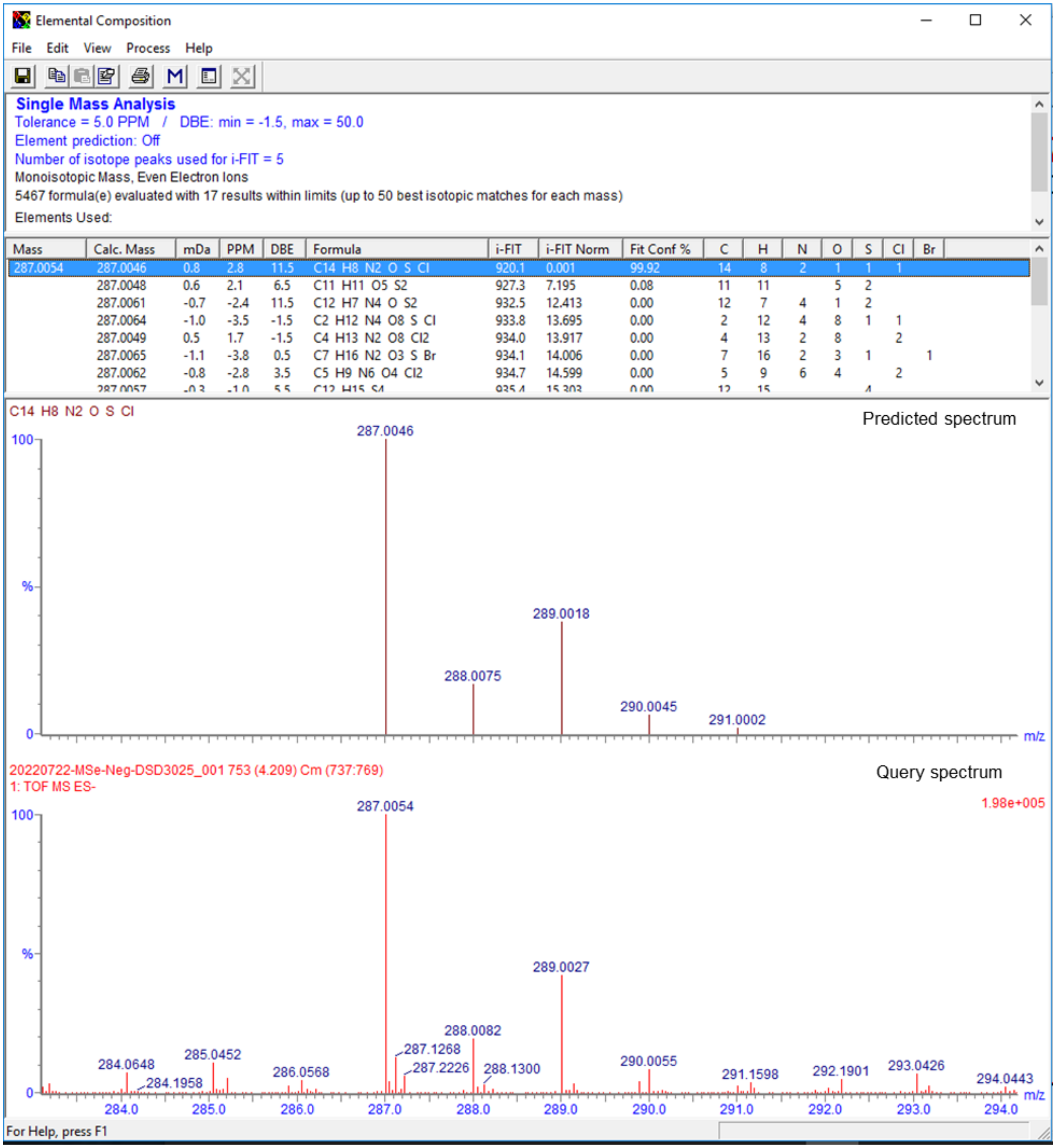

Figure S20. Formula prediction of Compound **7**. Chemical formula was accurately predicted (<5.0 ppm) based on the measured mass, % fit confidence, and isotopic model. The mass ion peak at *m/z* 287.0054 $[M-H]^-$ is a new thiocarbazole alkaloid from DSD3025^T with a neutral chemical formula of $C_{14}H_9N_2OSCl$ and double bond equivalent of 11.0.

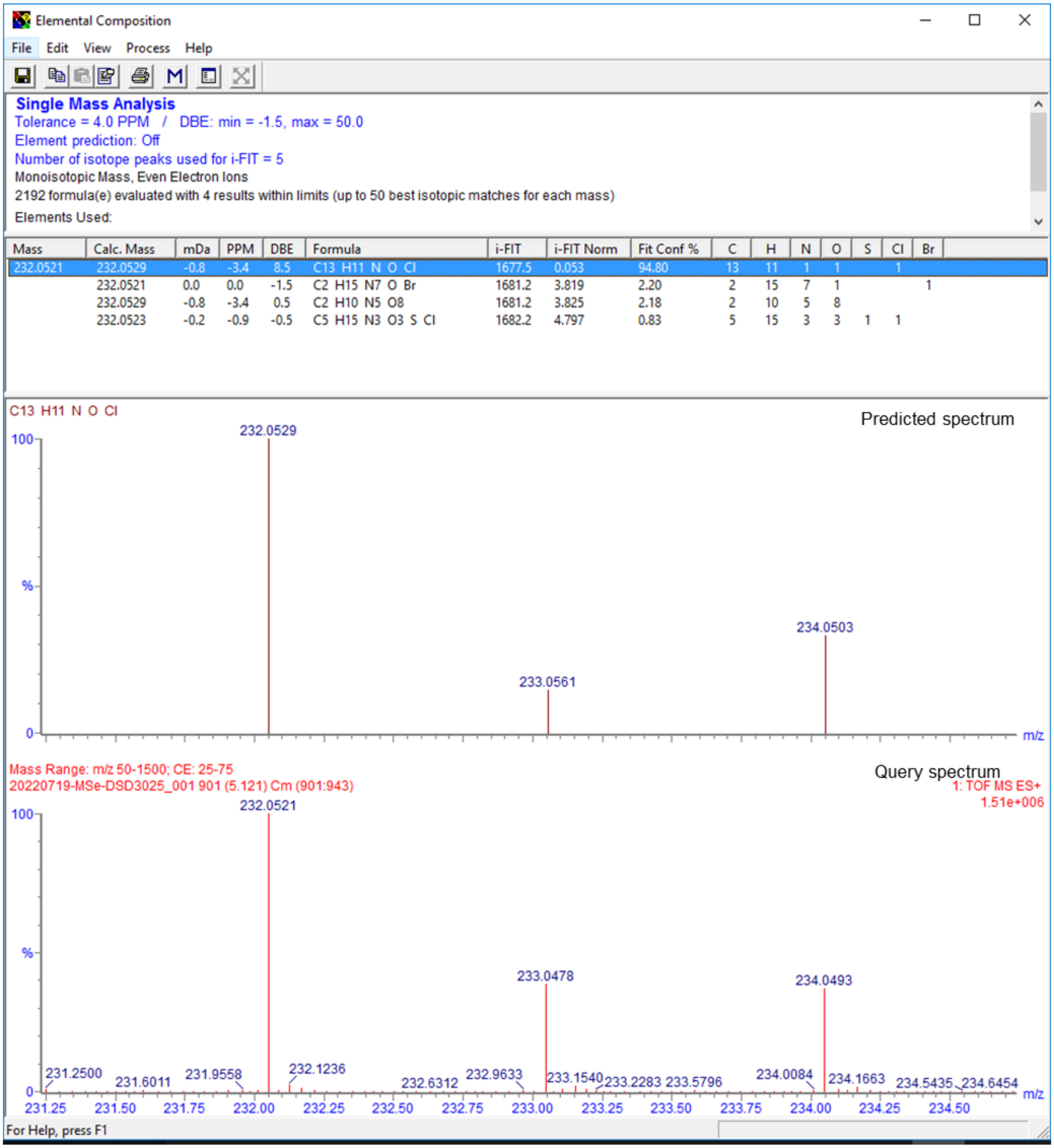

Figure S21. Formula prediction of chlorinated compound (*m/z* 232.0521 $[M+H]^+$) detected in the positive mode. Chemical formula was accurately predicted (<5.0 ppm) based on the measured mass, % fit confidence, and isotopic model. The mass ion peak at *m/z* 232.0529 $[M+H]^+$ was identified as chlocarbazomycin A with a neutral chemical formula of $C_{13}H_{10}NOCl$ and double bond equivalent of 9.0.

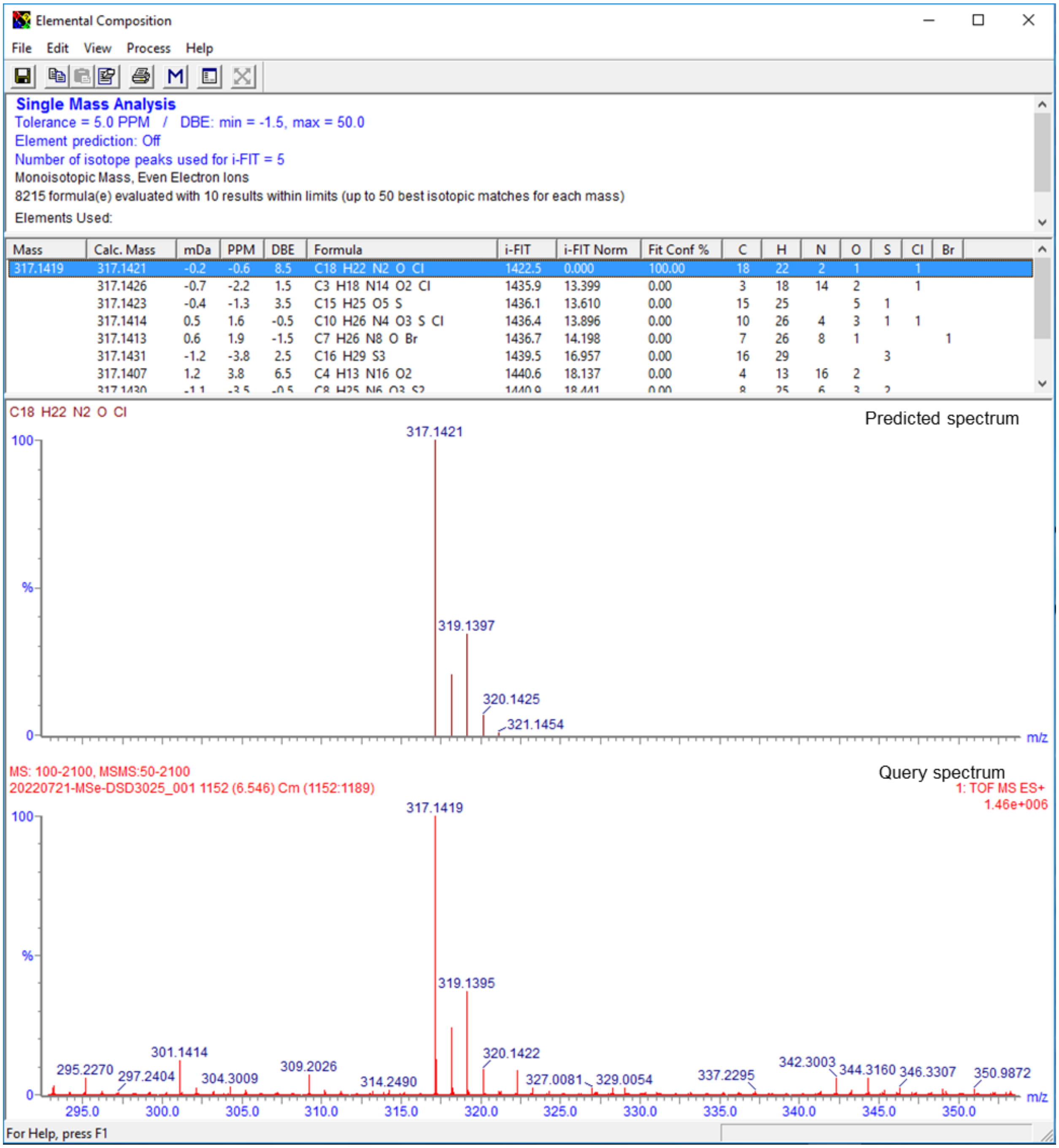

Figure S22. Formula prediction of chlorinated compound (*m/z* 317.1419 $[M+H]^+$) detected in the positive mode. Chemical formula was accurately predicted (<5.0 ppm) based on the measured mass, % fit confidence, and isotopic model. The mass ion peak at *m/z* 317.1419 $[M+H]^+$ is an unknown compound with a neutral chemical formula of $C_{18}H_{21}N_2OCl$ and double bond equivalent of 9.0.

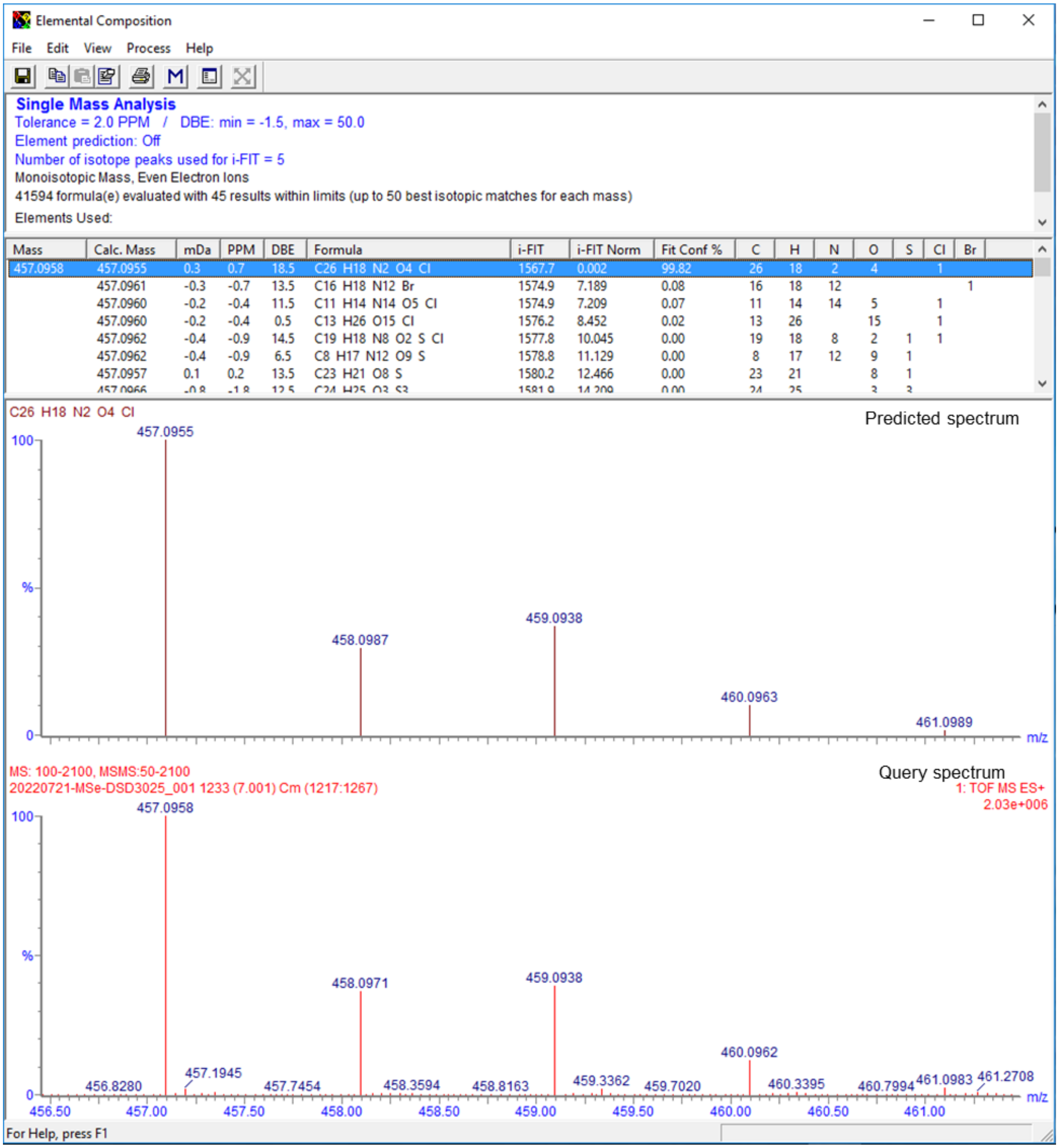

Figure S23. Formula prediction of chlorinated compound (*m/z* 457.0958 $[M+H]^+$) detected in the positive mode. Chemical formula was accurately predicted (<5.0 ppm) based on the measured mass, % fit confidence, and isotopic model. The mass ion peak at *m/z* 457.0958 $[M+H]^+$ is an unknown compound with a neutral chemical formula of $C_{26}H_{17}N_2O_4Cl$ and double bond equivalent of 19.0.

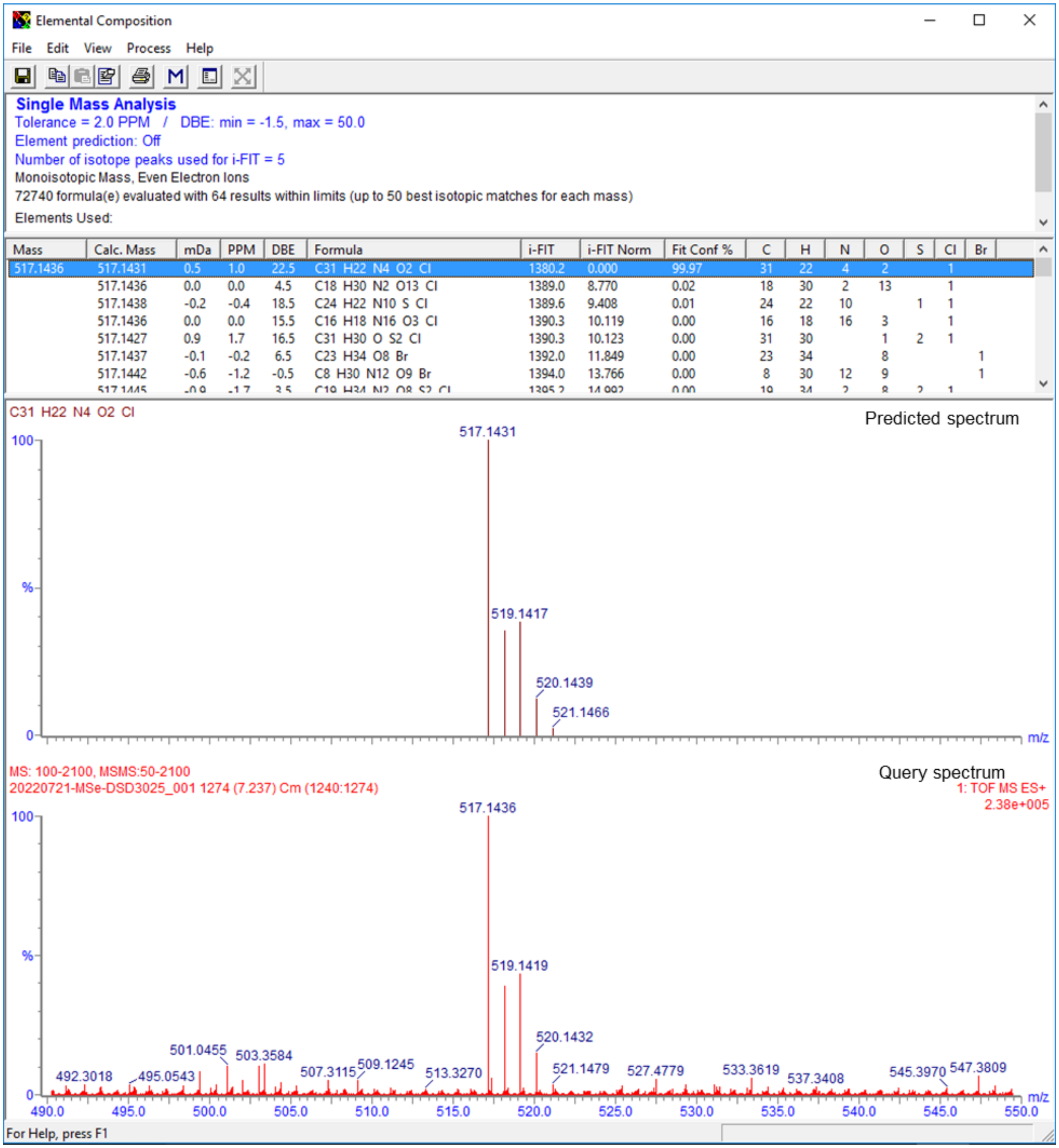

Figure S24. Formula prediction of chlorinated compound (*m/z* 517.1436 $[M+H]^+$) detected in the positive mode. Chemical formula was accurately predicted (<5.0 ppm) based on the measured mass, % fit confidence, and isotopic model. The mass ion peak at *m/z* 517.1436 $[M+H]^+$ is an unknown compound with a neutral chemical formula of $C_{31}H_{21}N_4O_2Cl$ and double bond equivalent of 23.

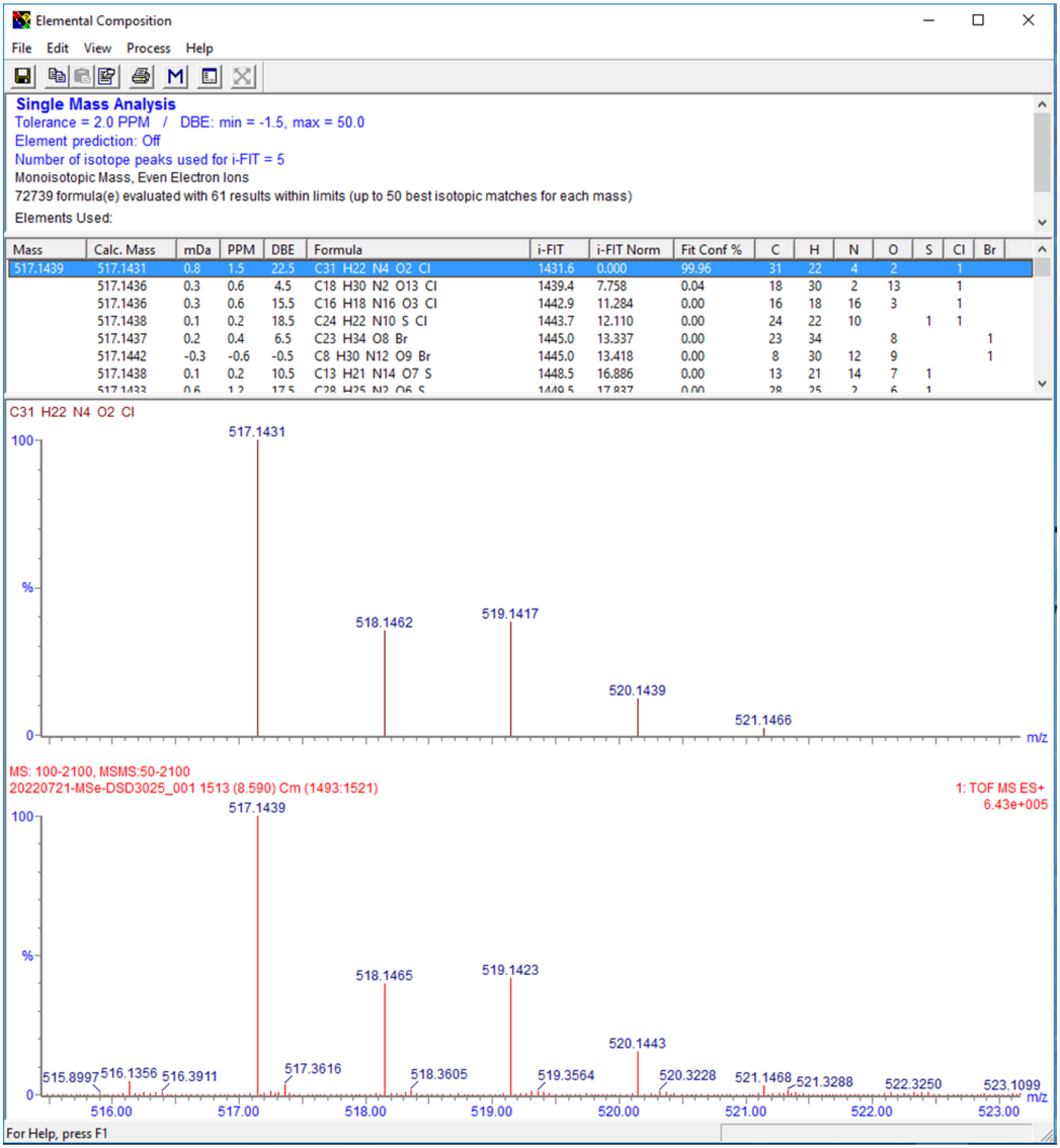

Figure S25. Formula prediction of chlorinated compound (*m/z* 517.1439 [M+H]$^+$) detected in the positive mode. Chemical formula was accurately predicted (<5.0 ppm) based on the measured mass, % fit confidence, and isotopic model. The mass ion peak at *m/z* 517.1439 [M+H]$^+$ is an unknown compound with a neutral chemical formula of $C_{31}H_{21}N_4O_2Cl$ and double bond equivalent of 23.

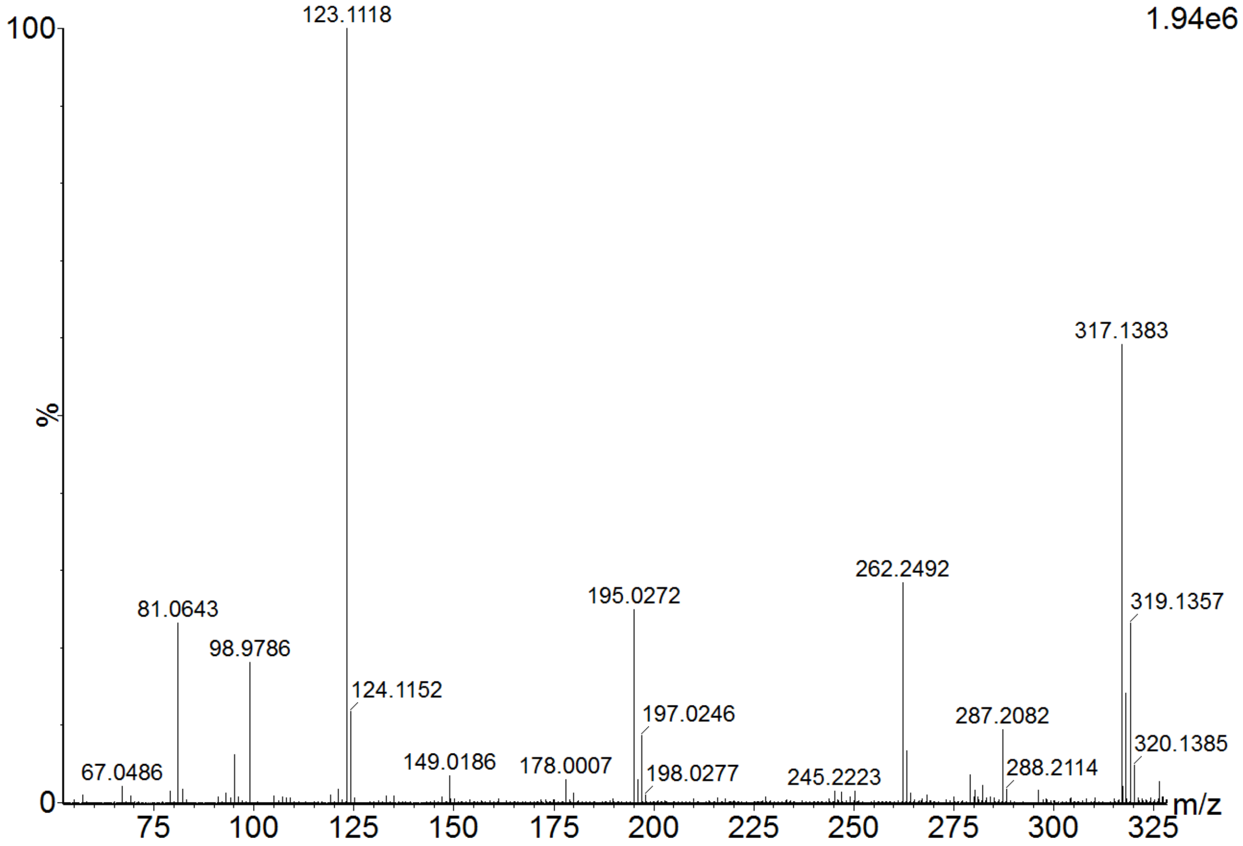

Figure S26. MS/MS analysis of chlorinated compound (*m/z* 317.1419 $[M+H]^+$) from DSD3025^T. MS/MS data of precursor *m/z* 317.1419 $[M+H]^+$ was acquired in the positive mode and Collision Energy (CE) at -20V.

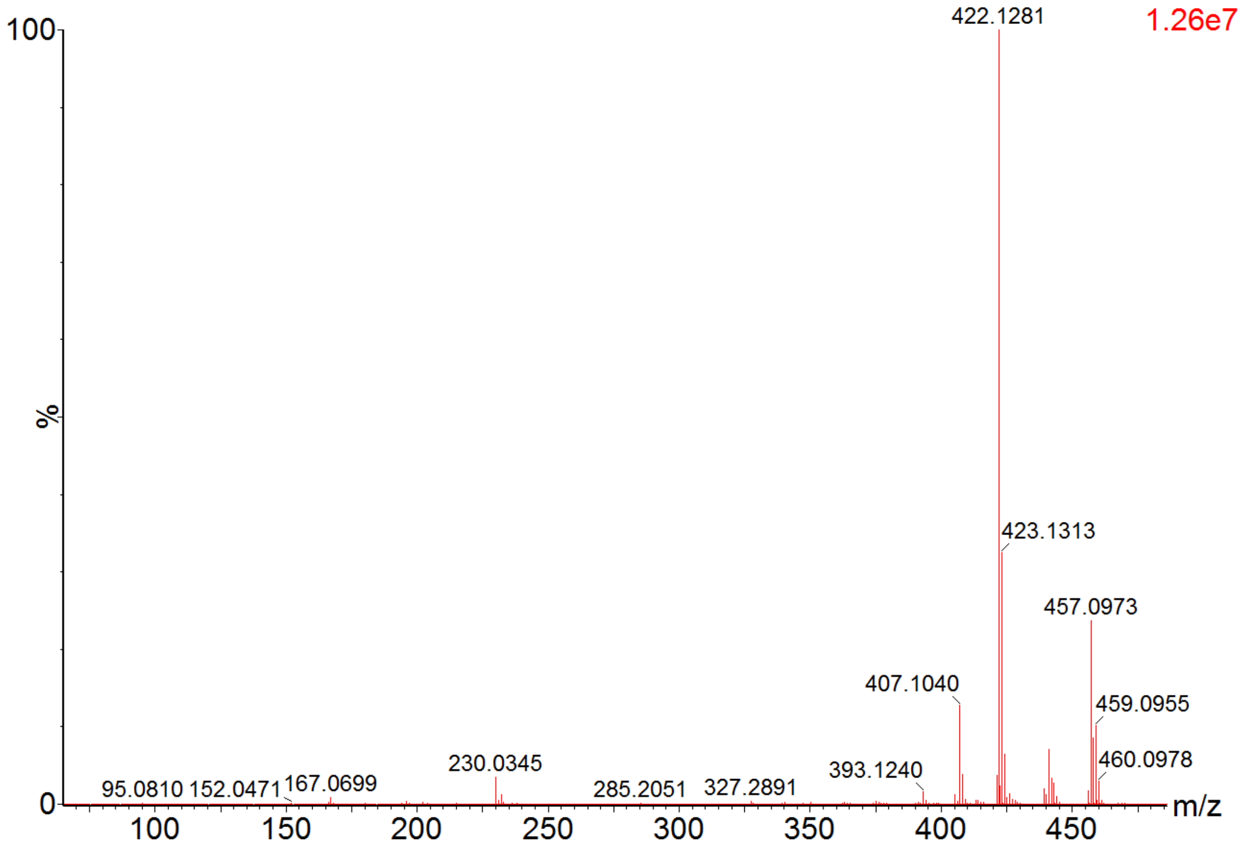

Figure S27. MS/MS analysis of chlorinated compound (*m/z* 457.0958 $[M+H]^+$) from DSD3025^T. MS/MS data of precursor *m/z* 457.0958 $[M+H]^+$ was acquired in the positive mode and Collision Energy (CE) at -20V.

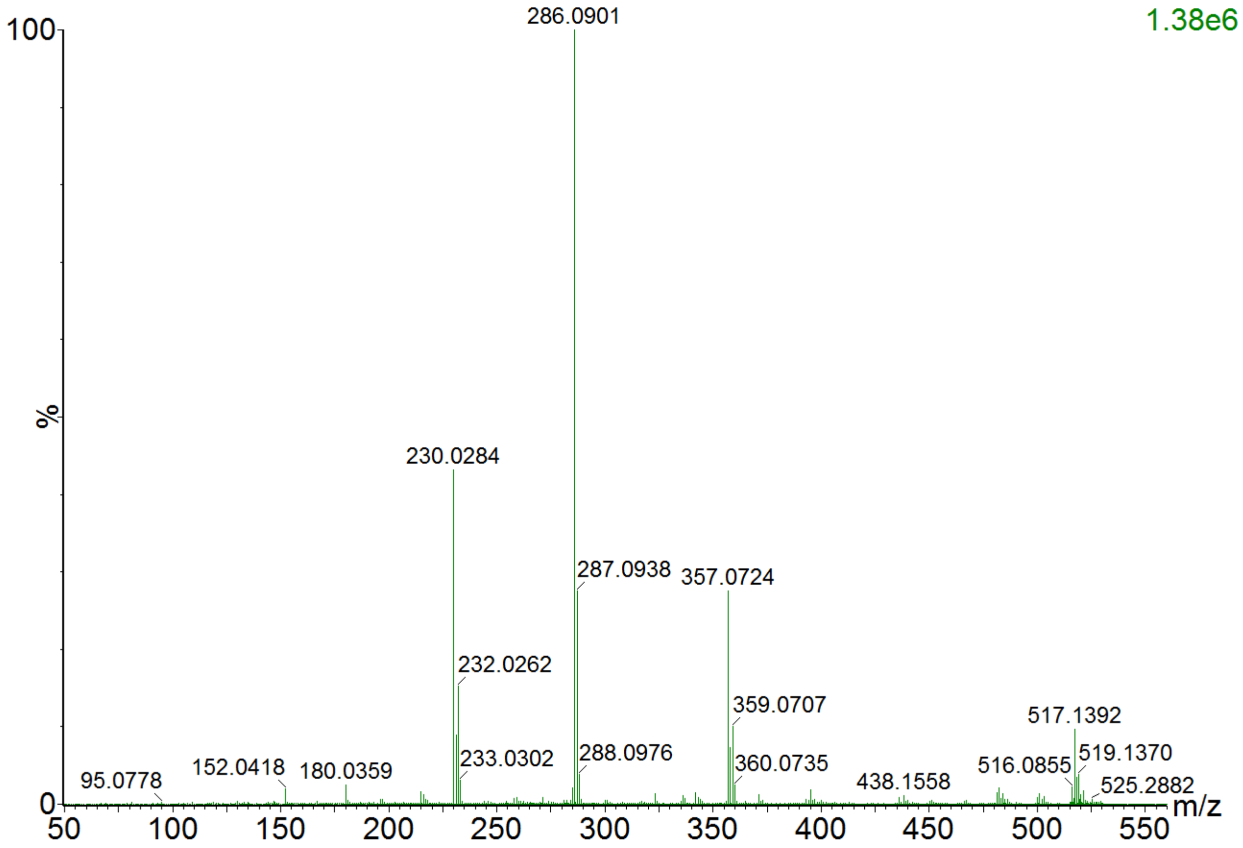

Figure S28. MS/MS analysis of chlorinated compound (*m/z* 517.1436 $[M+H]^+$) from DSD3025^T. MS/MS data of precursor *m/z* 517.1436 $[M+H]^+$ was acquired in the positive mode and Collision Energy (CE) at -30V.

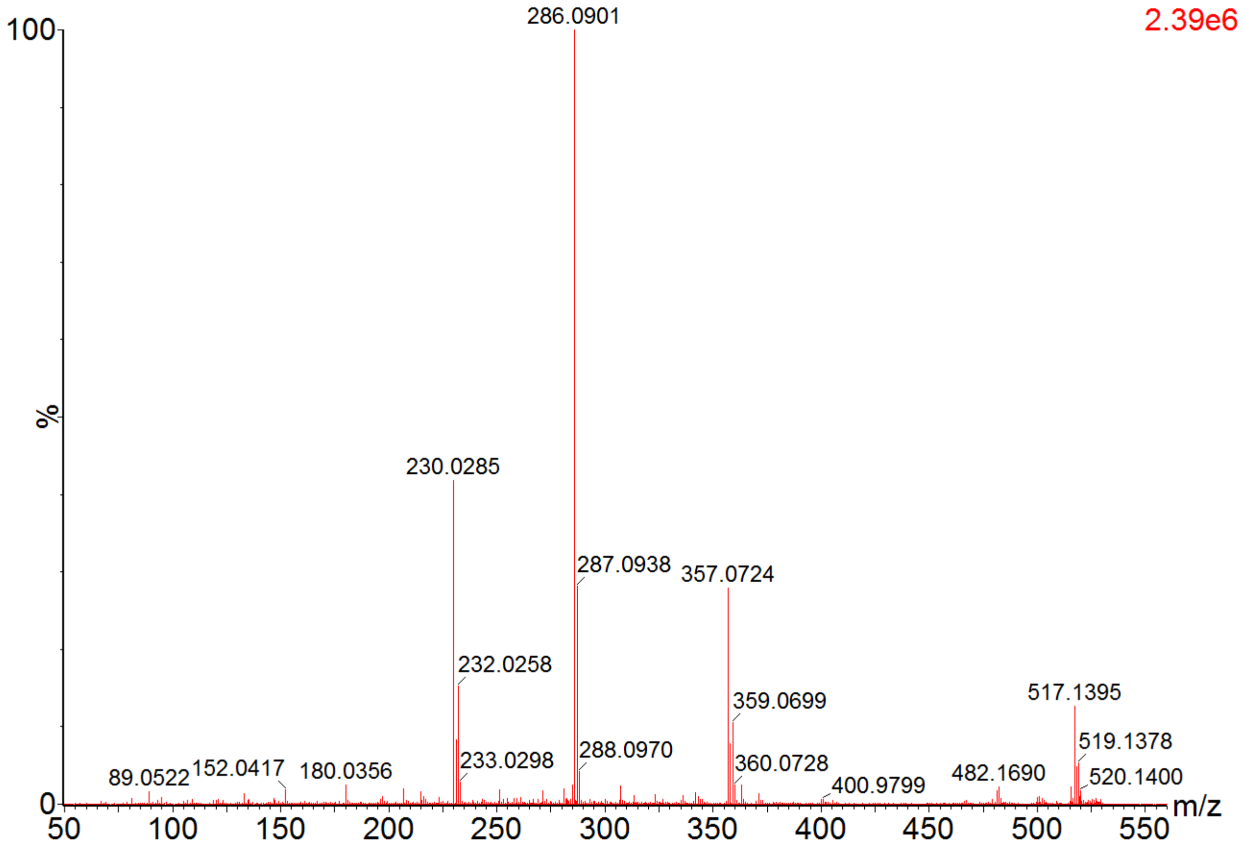

Figure S29. MS/MS analysis of chlorinated compound (m/z 517.1439 [M+H]+) from DSD3025^{T}. MS/MS data of precursor *m/z* 517.1439 $[M+H]^+$ was acquired in the positive mode and Collision Energy (CE) at -30V.

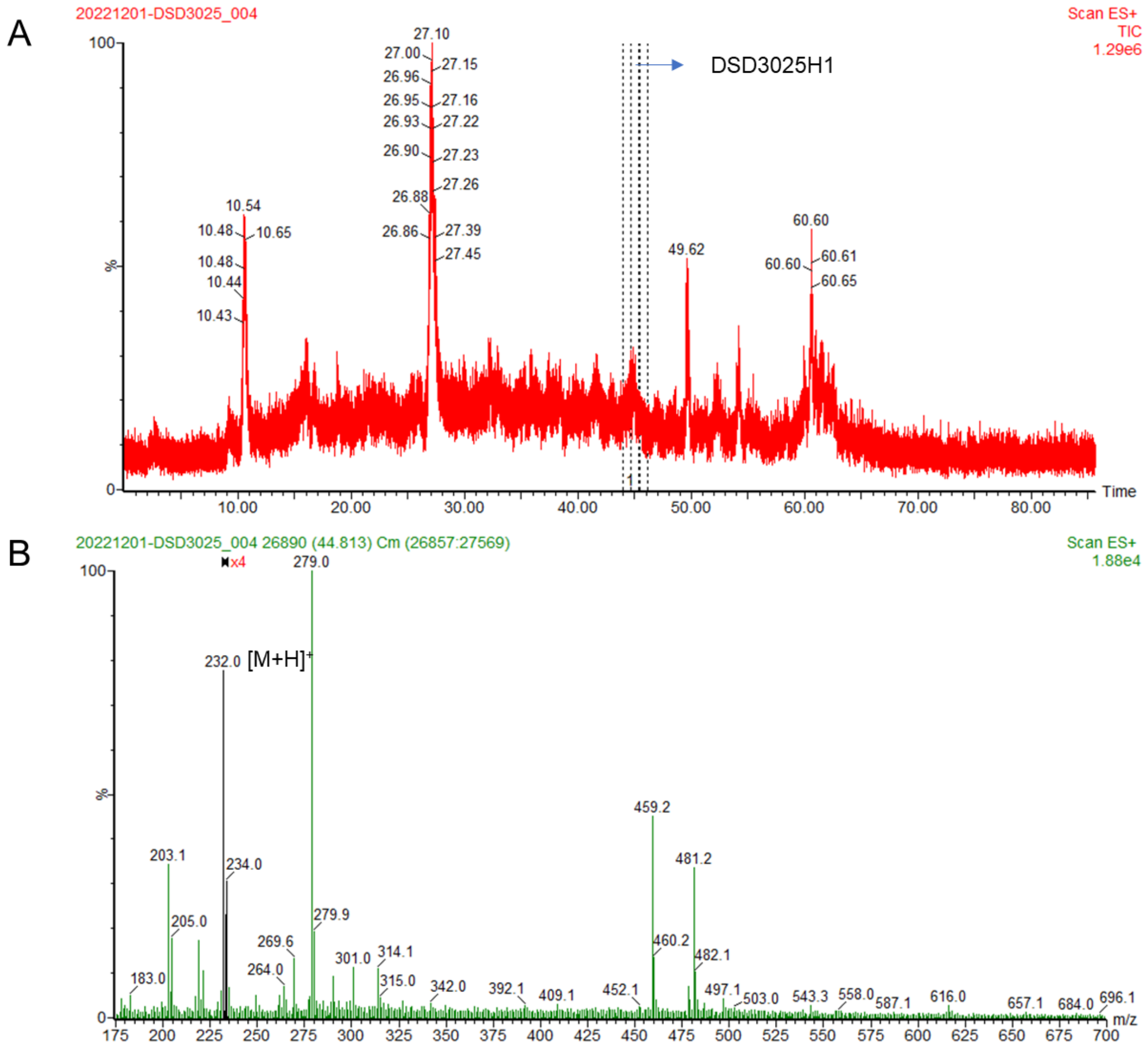

Figure S30. Mass-Directed Purification of *S. tubbatahanensis* DSD3025^T Extract. (A) Total Ion Chromatogram (TIC) in the positive polarity and collection (broken lines) of target chlorinated compound *m/z* 232 $[M+H]^+$. (B) Target mass ion peak at *m/z* 232.0 $[M+H]^+$ magnified 4x was isolated and detected in the collected fraction using a single-quadrupole mass spectrometer. Mass ion scans in the positive polarity were performed at mass ion range of 150 – 700 *m/z*.

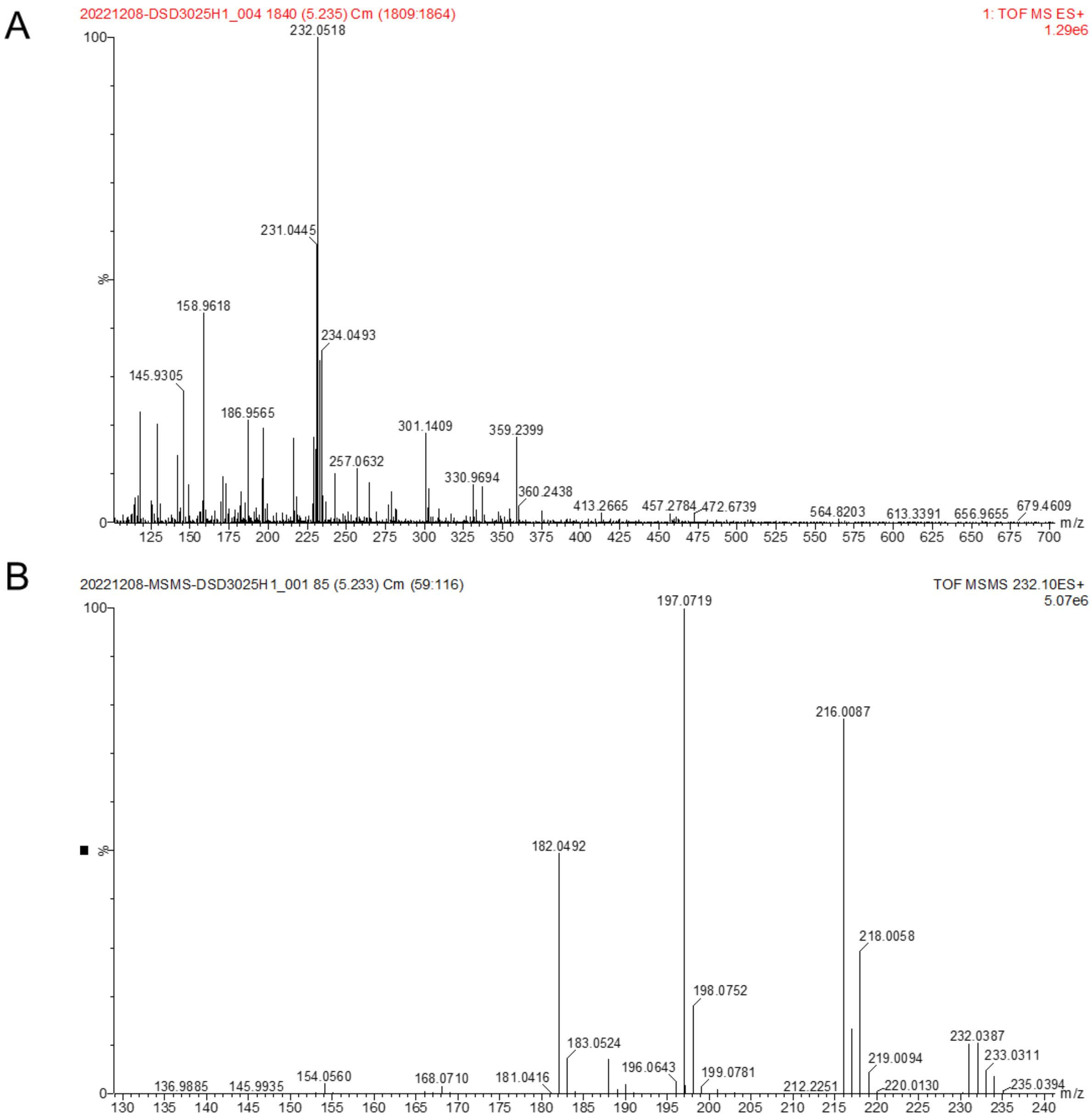

Figure S31. High-Resolution Mass Spectrometry Analysis of Collected Fraction DSD3025H1. Fraction DSD3025H1 was isolated via mass directed preparatory HPLC purification of *S. tubbatahanensis* DSD3025^T Extract. (A) Mass ion peaks at *m/z* 232.0518 $[M+H]^+$ was detected at t_R 5.24 min, consistent with initial metabolite profiling. (B) MS/MS analysis of *m/z* 232.0518 $[M+H]^+$ also showed consistent fragmentation with initial metabolite profiling.

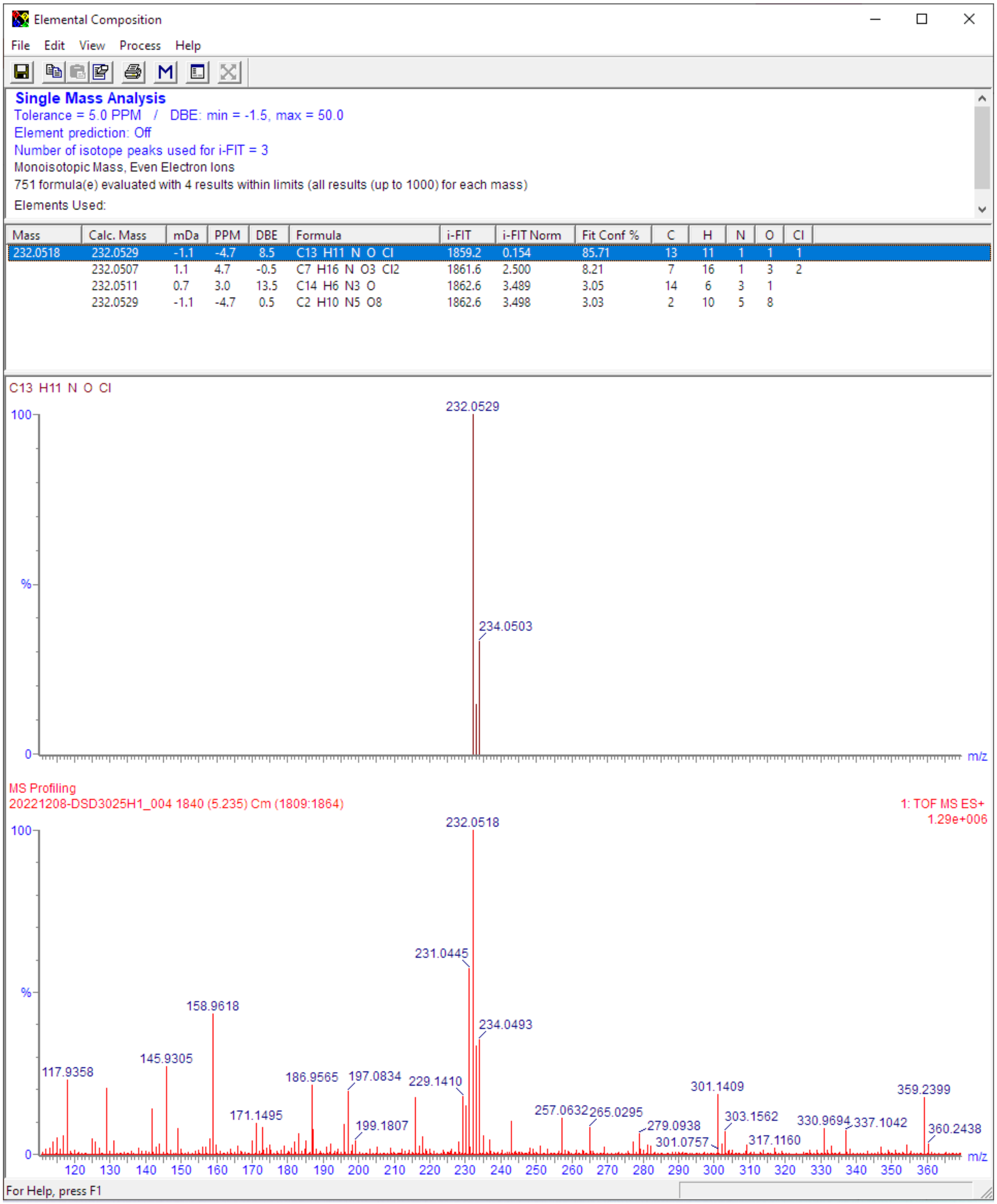

Figure S32. Formula prediction of the isolated fraction of DSD3025H1. Mass ion peak at *m/z* 232.0518 $[M+H]^+$ was detected in the positive mode from DSD3025H1. Chemical formula was accurately predicted (<5.0 ppm) based on the measured mass, % fit confidence, and isotopic model which is consistent with chlocarbazomycin A **1** ($C_{13}H_{10}NOCl$).

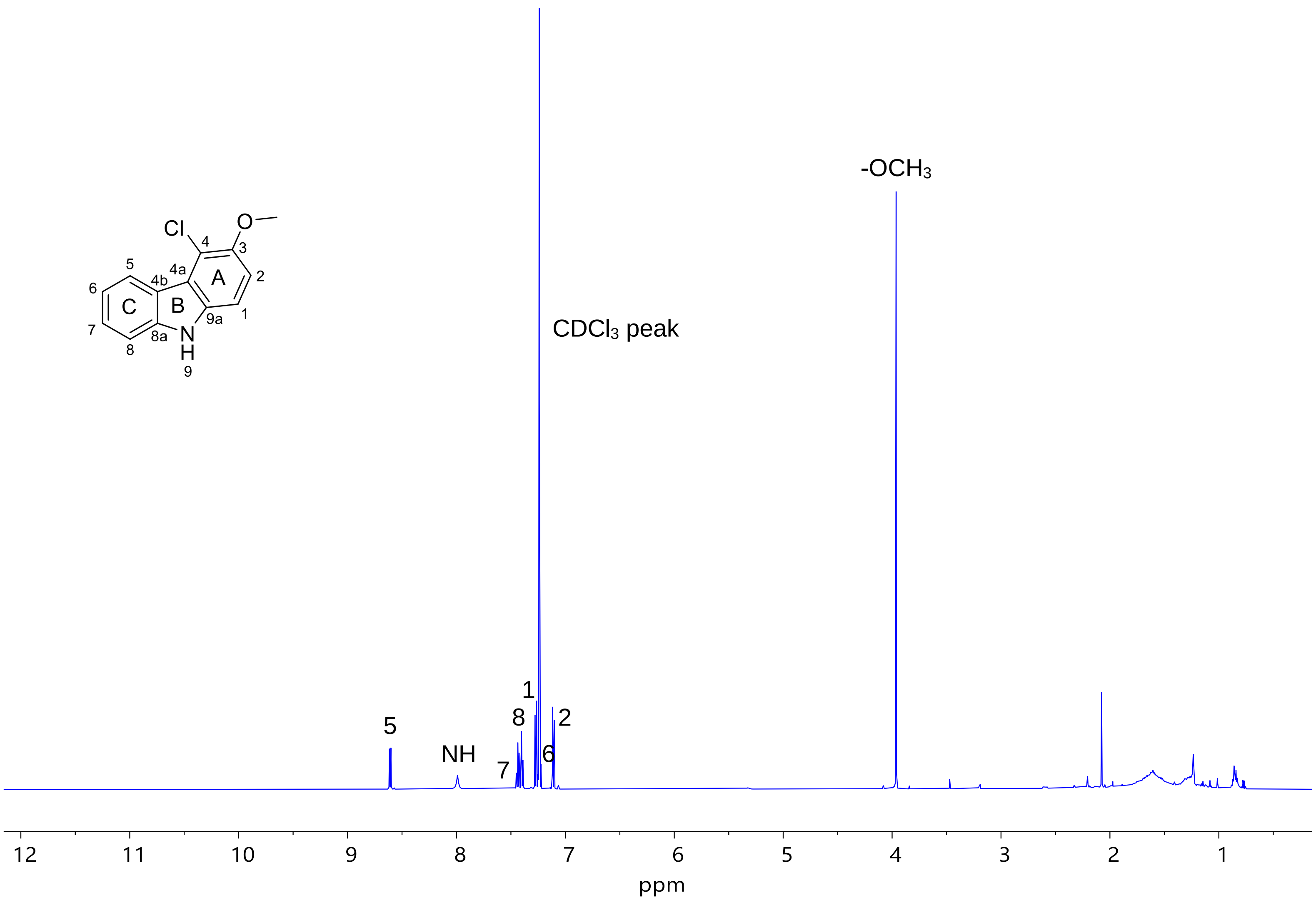

Figure S33. ^{1}H NMR Spectrum of **1** (600 MHz, $CDCl_3$).

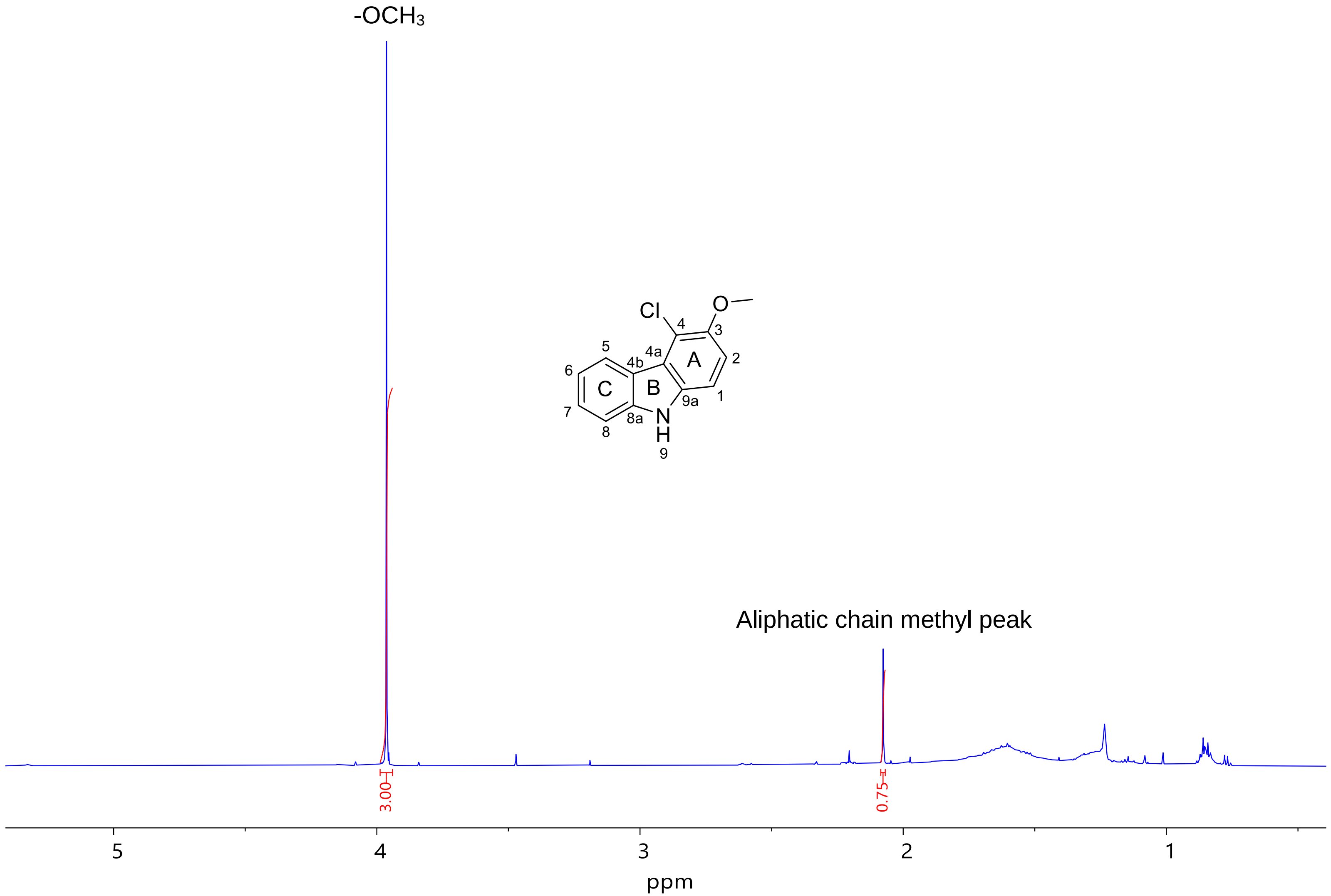

Figure S34. ^{1}H NMR Spectrum of **1** (600 MHz, $CDCl_3$, expansion, aliphatic region).

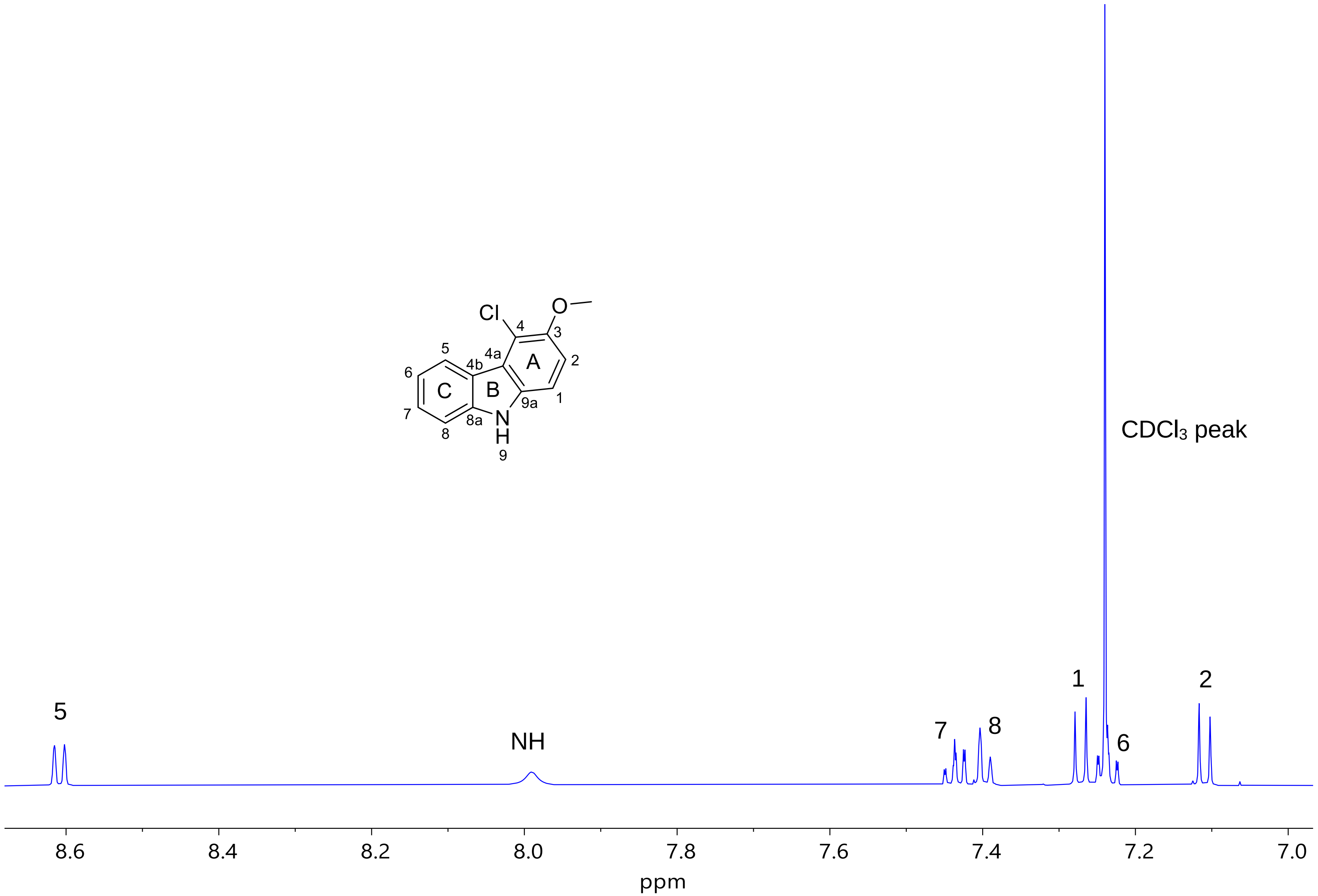

Figure S35. ^{1}H NMR Spectrum of **1** (600 MHz, $CDCl_3$, expansion, aromatic region).

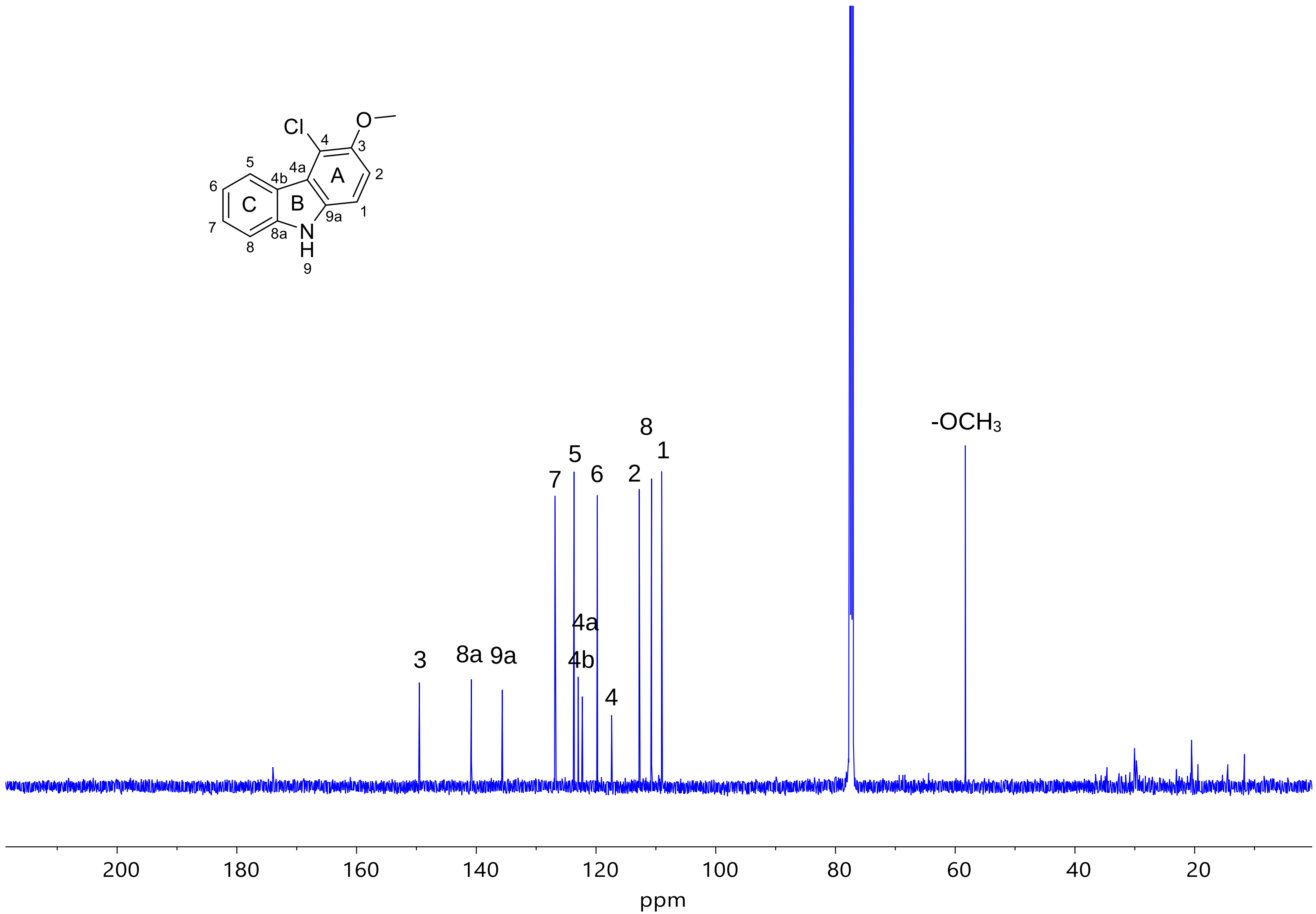

Figure S36. ^{1}H-decoupled ^{13}C NMR Spectrum of **1** (150 MHz, $CDCl_3$).

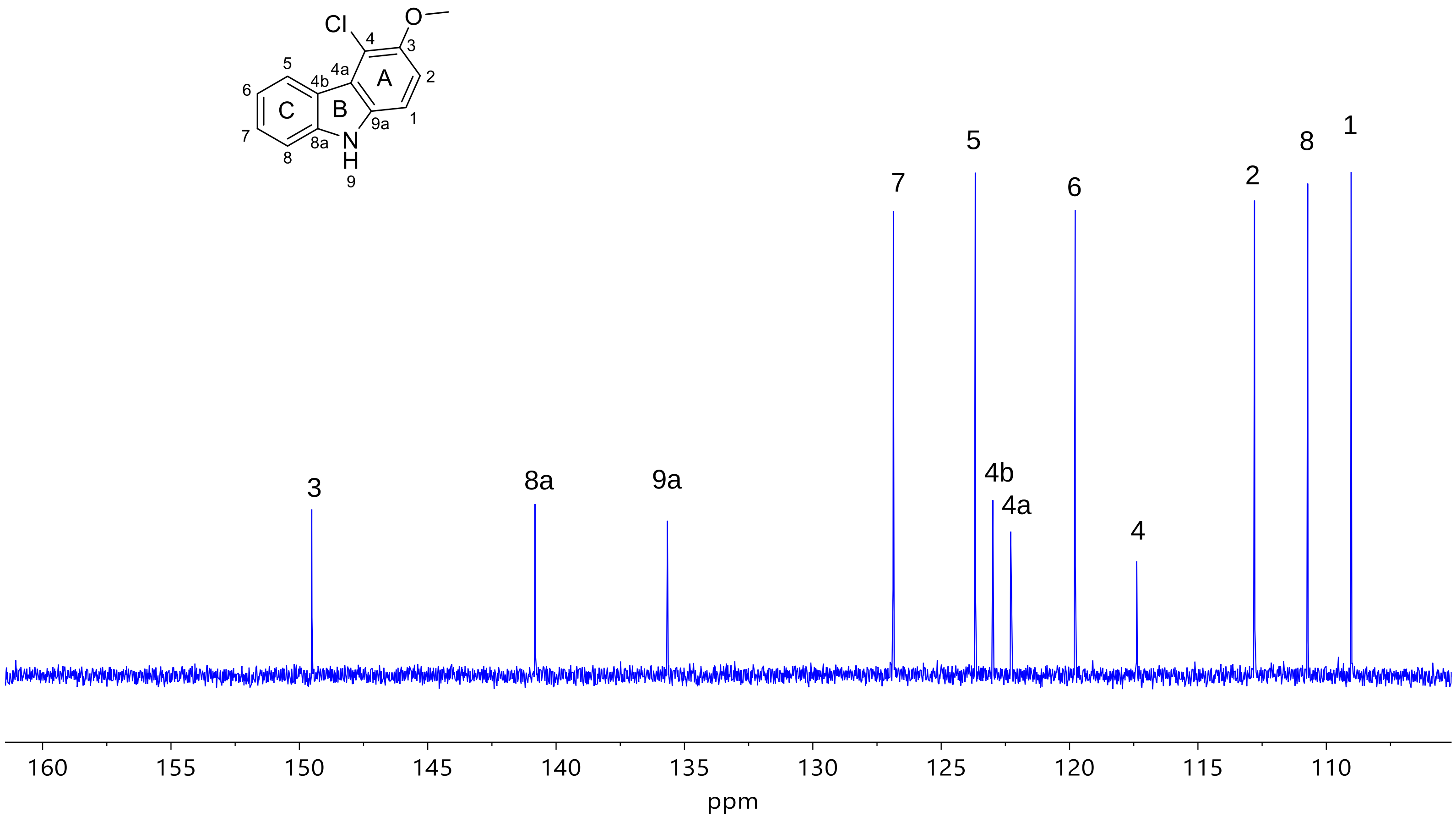

Figure S37. ^{1}H-decoupled ^{13}C NMR Spectrum of **1** (150 MHz, $CDCl_3$, expansion).

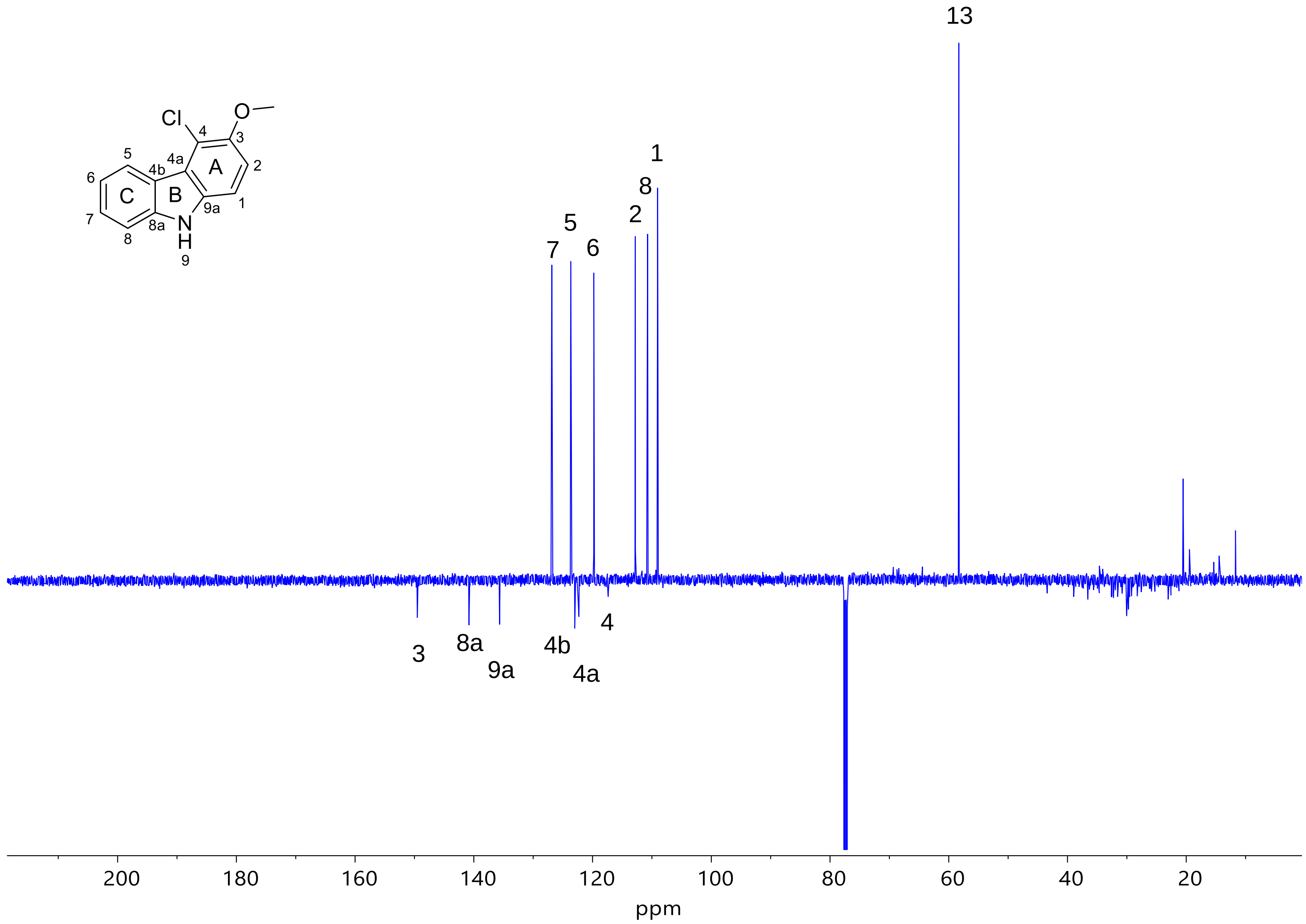

Figure S38. ^{13}C DEPTQ135 NMR Spectrum of **1** (150 MHz, $CDCl_3$). ^{13}C DEPTQ135 shows quaternary carbons as inverted peaks.

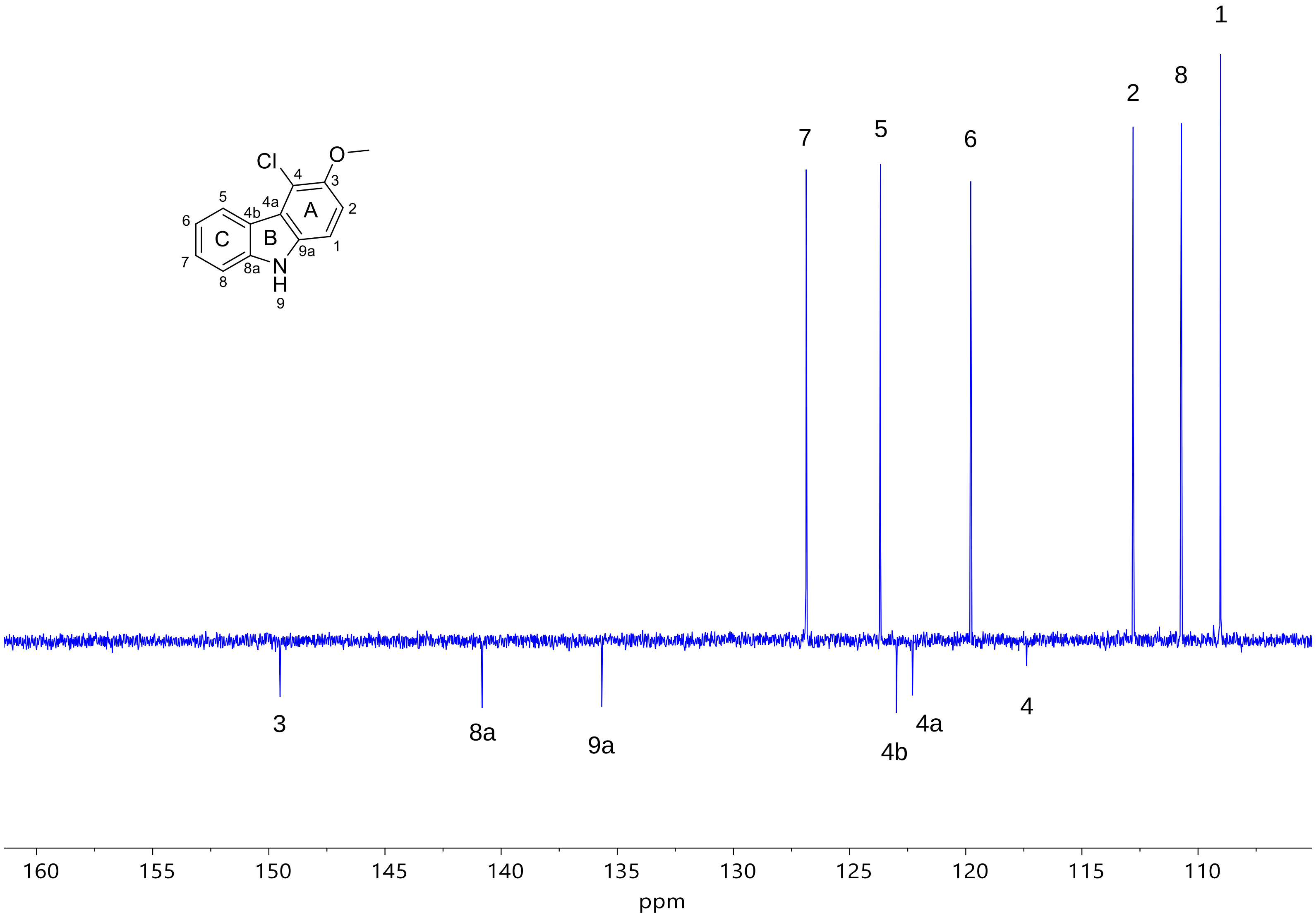

Figure S39. ^{13}C DEPTQ135 NMR Spectrum of **1** (150 MHz, $CDCl_3$, expansion).

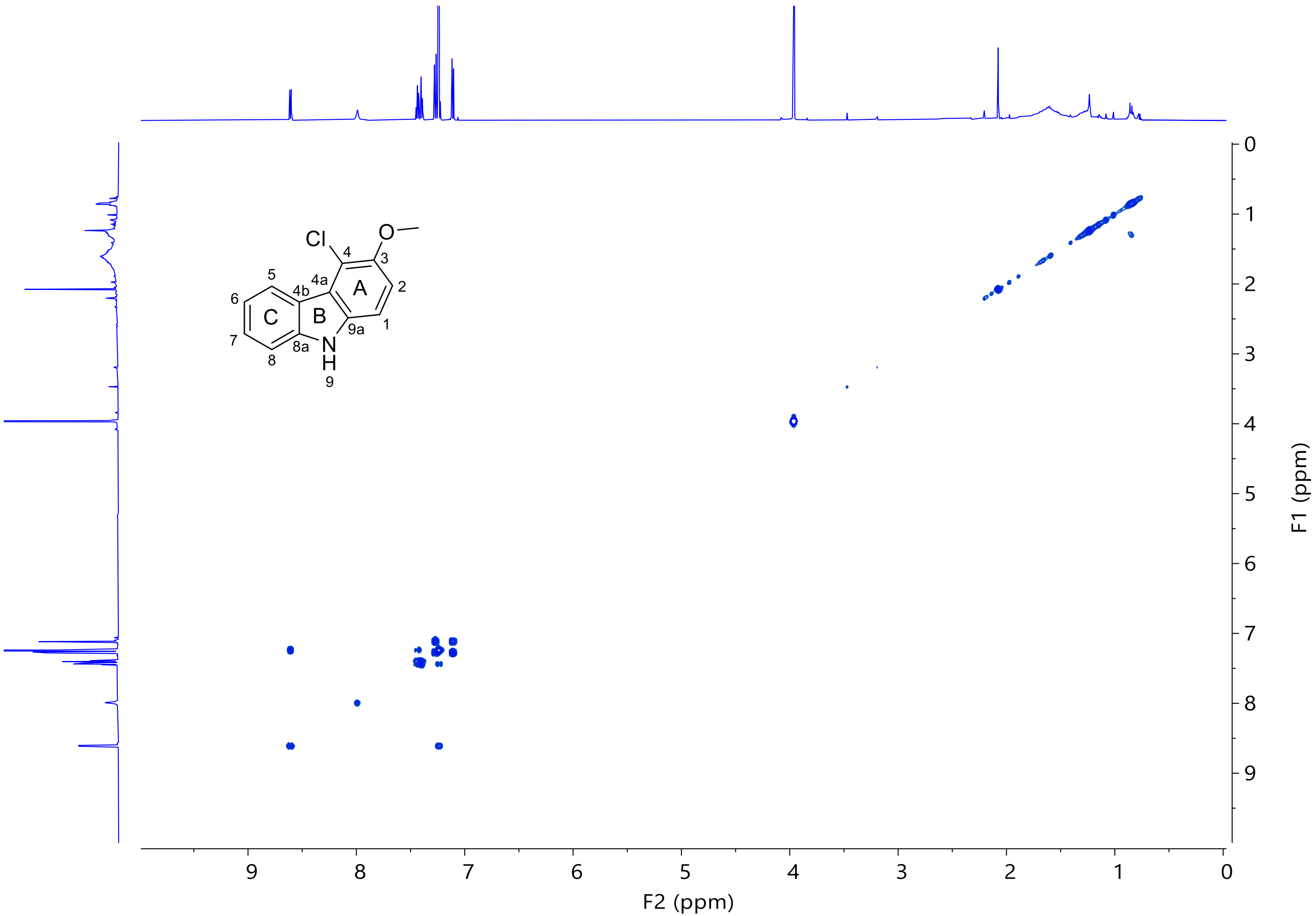

Figure S40. COSY NMR Spectrum of **1** (600 MHz, $CDCl_3$).

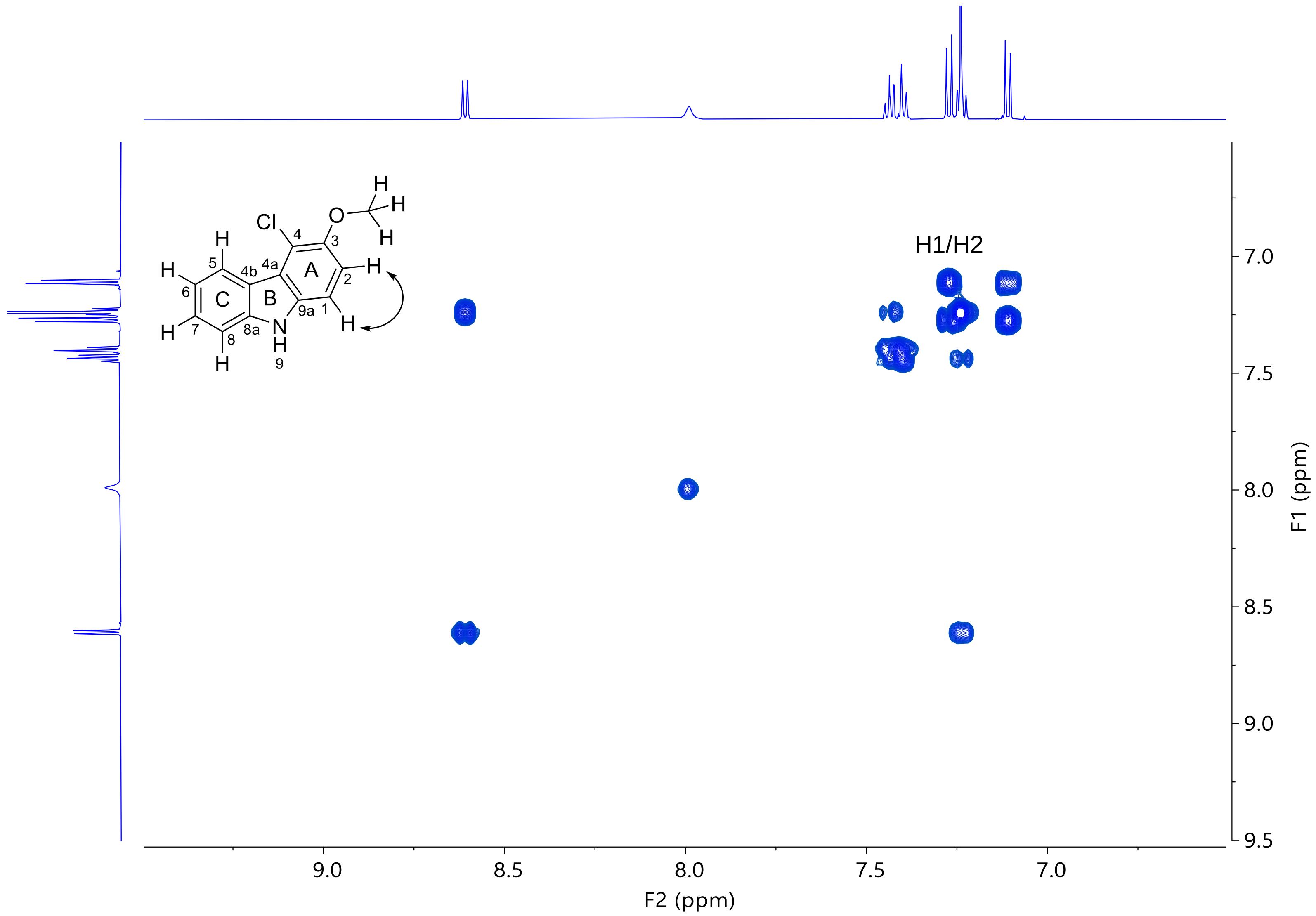

Figure S41. COSY NMR Spectrum of **1** (600 MHz, $CDCl_3$, expansion).

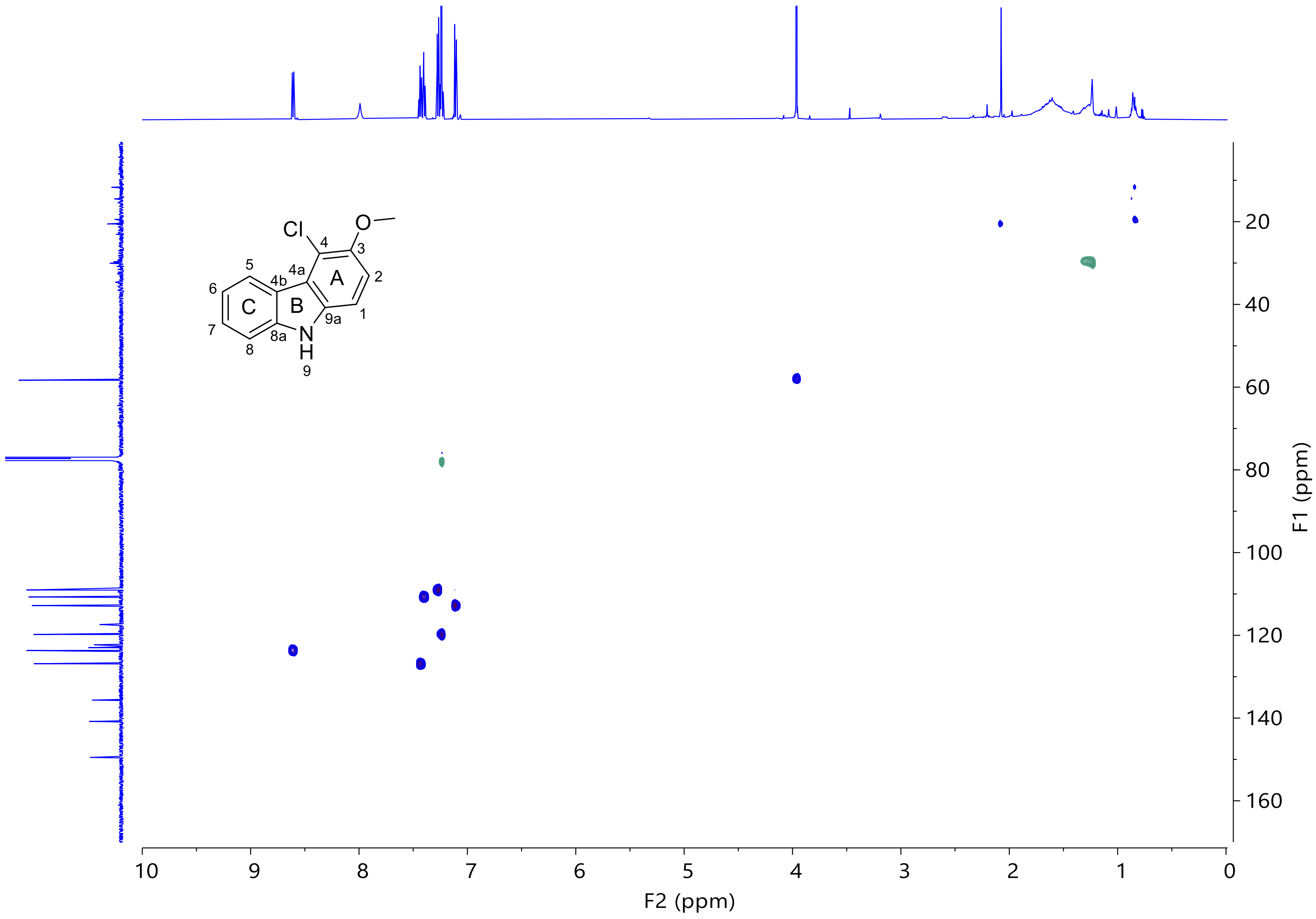

Figure S42. HSQC NMR Spectrum of **1** (600 MHz, $CDCl_3$).

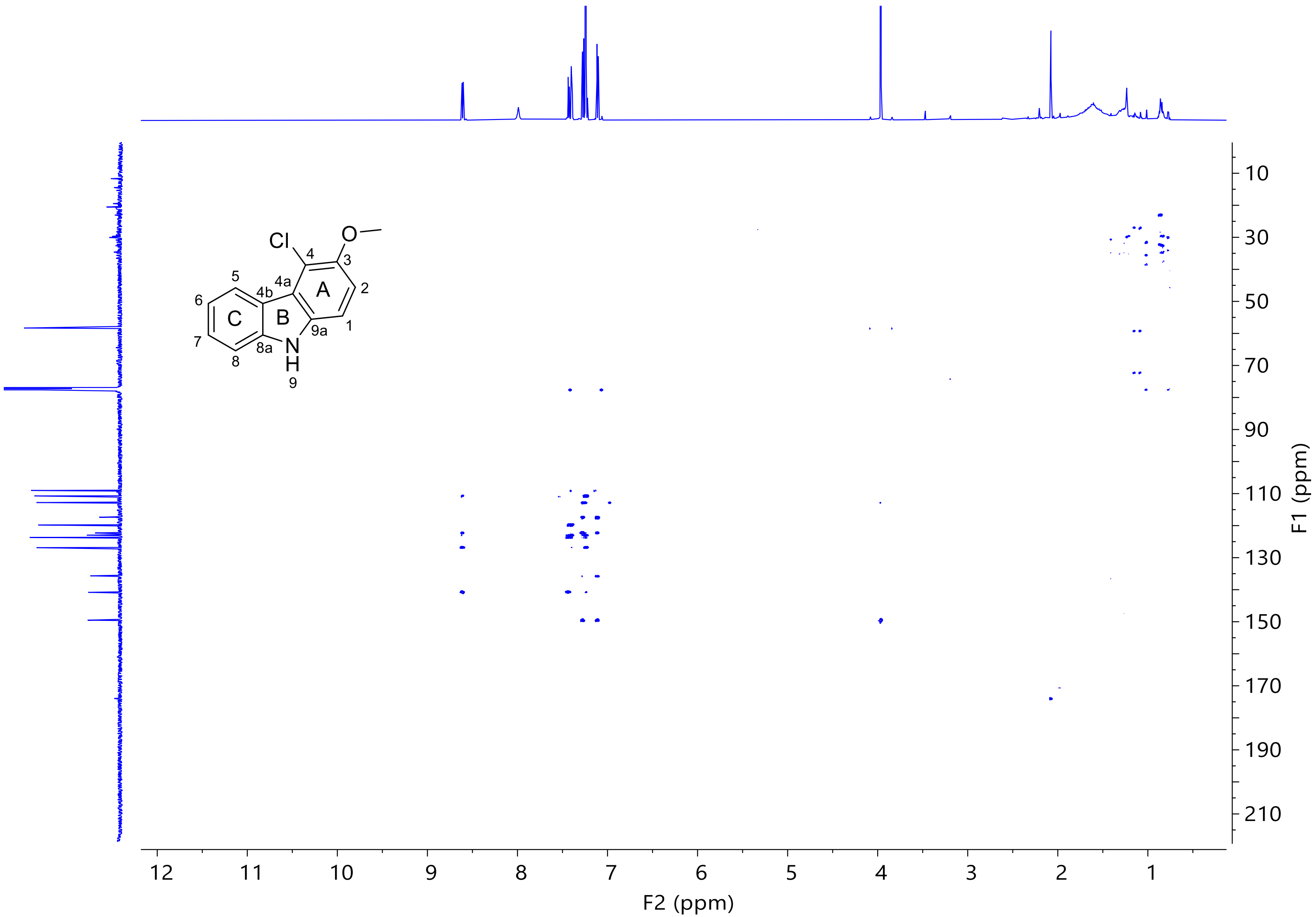

Figure S43. HMBC NMR Spectrum of **1** (600 MHz, $CDCl_3$, J_{CH} = 6 Hz).

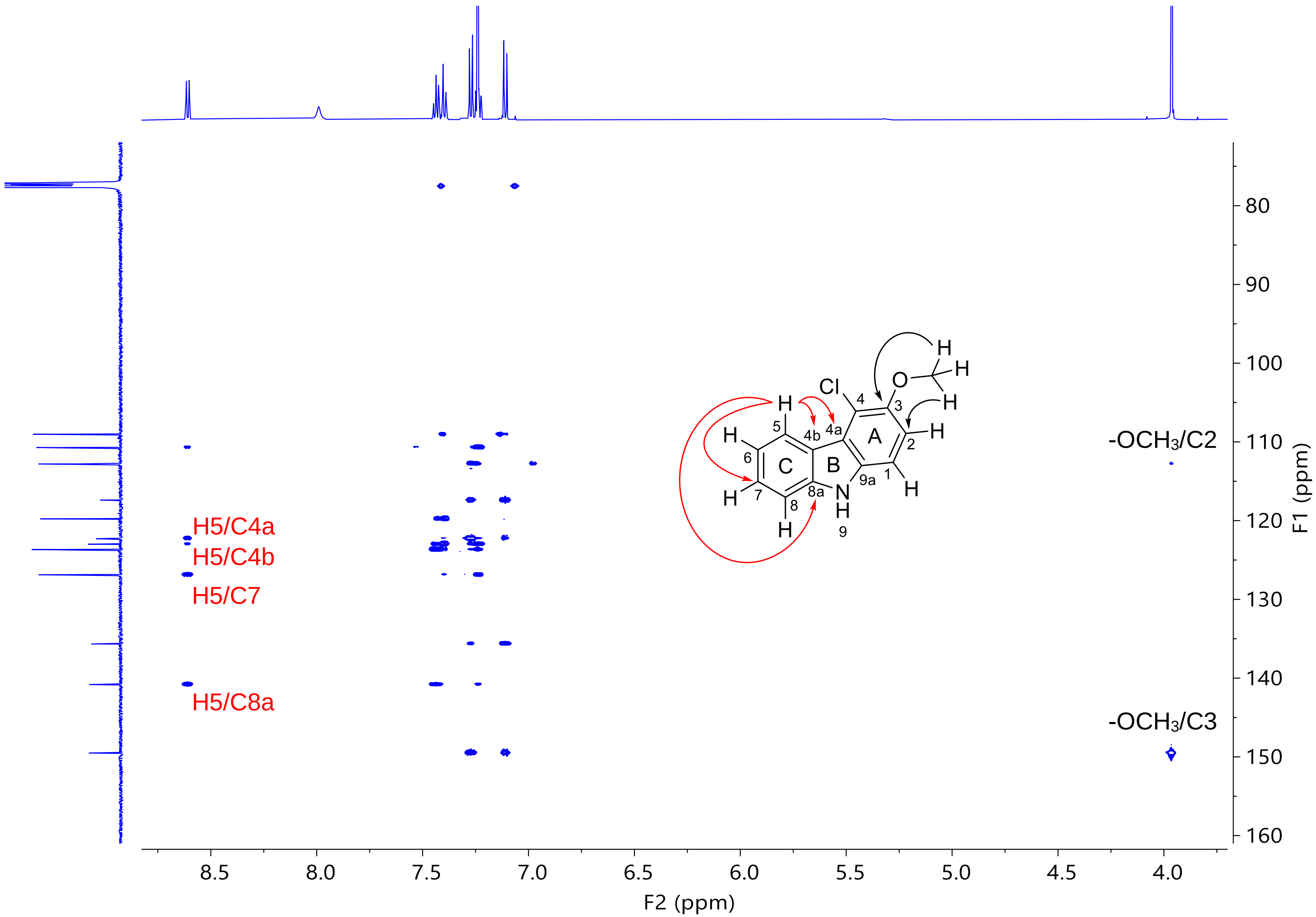

Figure S44. HMBC NMR Spectrum of **1** (600 MHz, $CDCl_3$, expansion, J_{CH} = 6 Hz).

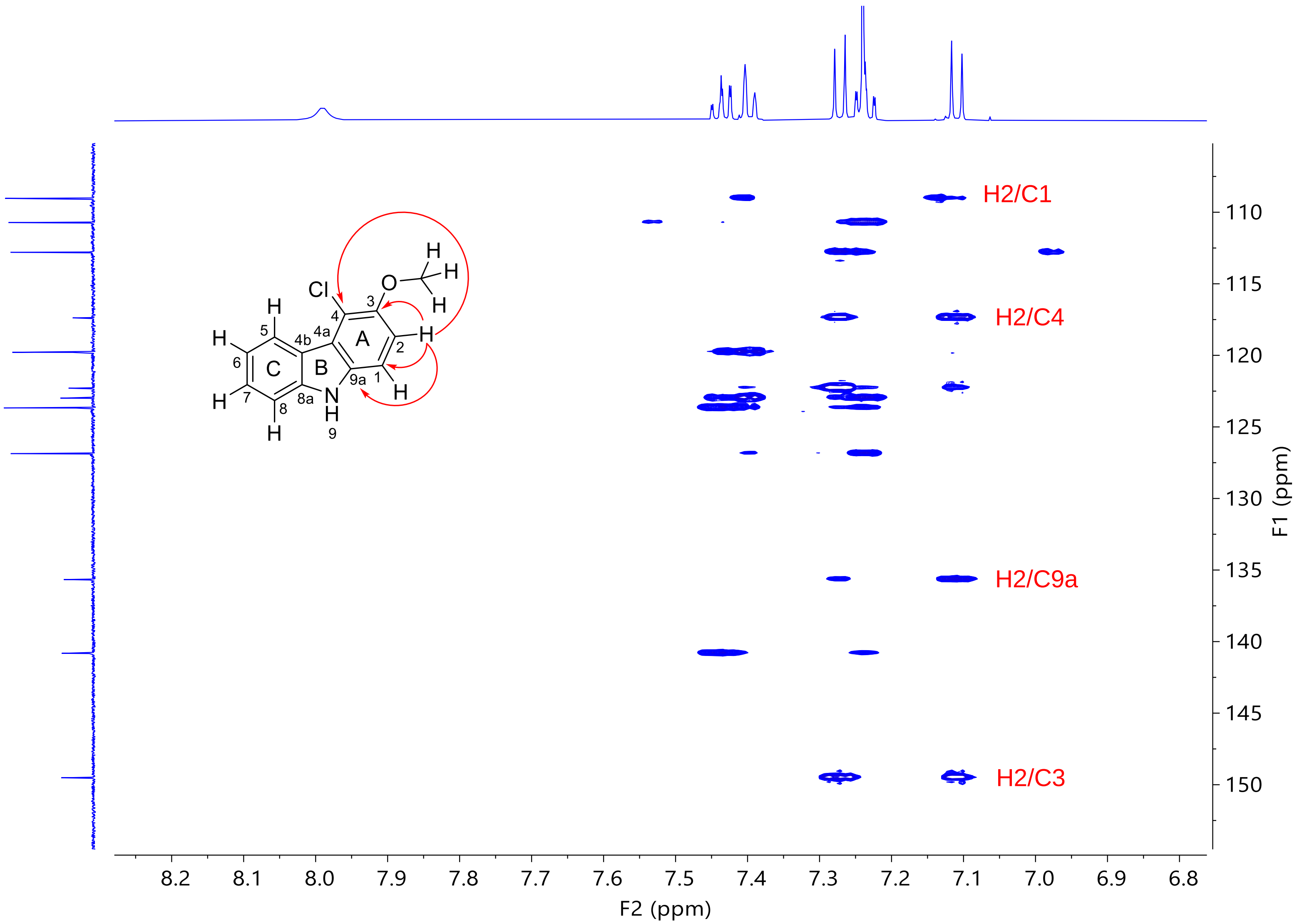

Figure S45. HMBC NMR Spectrum of **1** (600 MHz, $CDCl_3$, expansion, J_{CH} = 6 Hz).

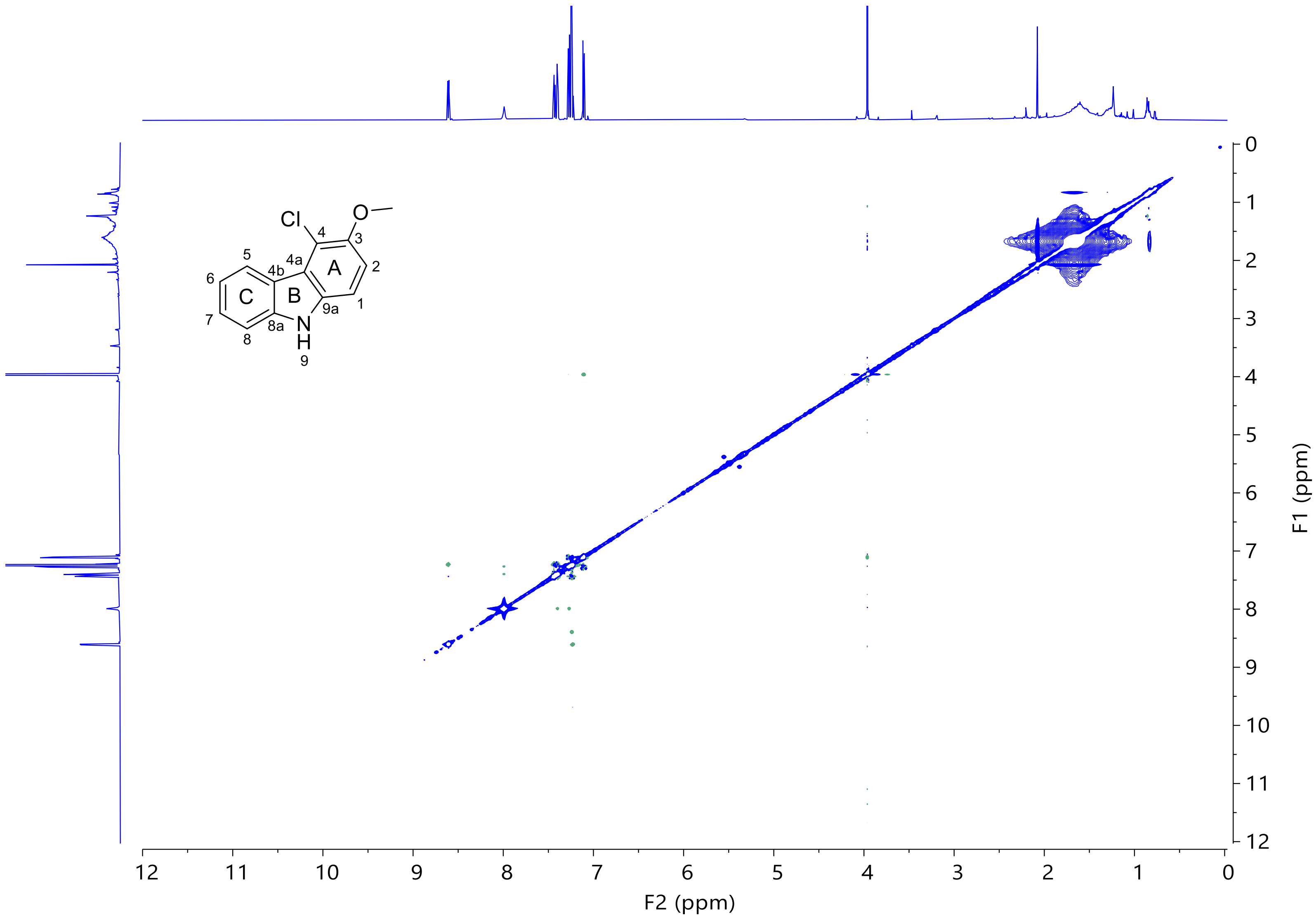

Figure S46. NOESY NMR Spectrum of **1** (600 MHz, $CDCl_3$, mixing time = 750 ms).

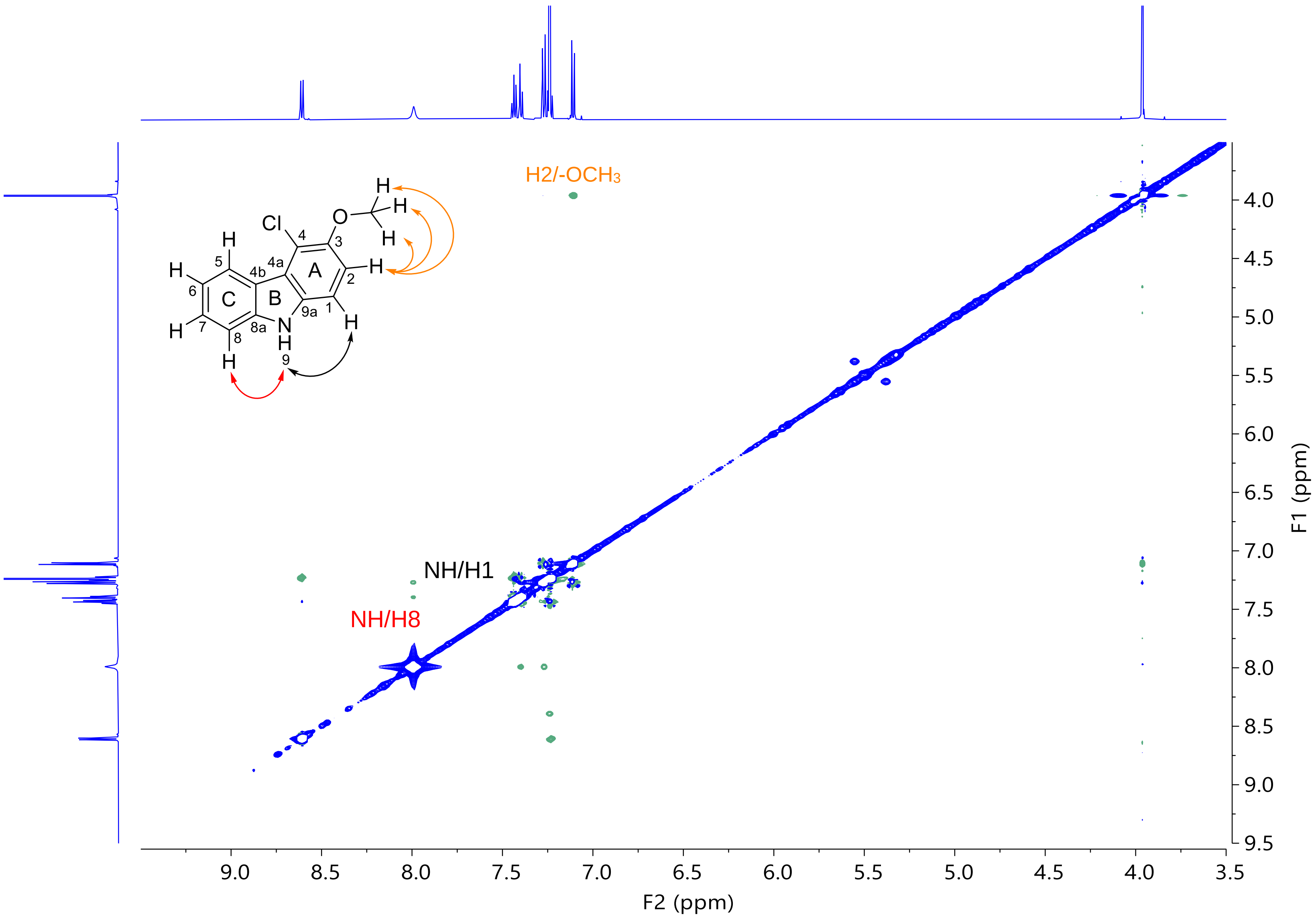

Figure S47. NOESY NMR Spectrum of **1** (600 MHz, $CDCl_3$, mixing time = 750 ms, expansion).

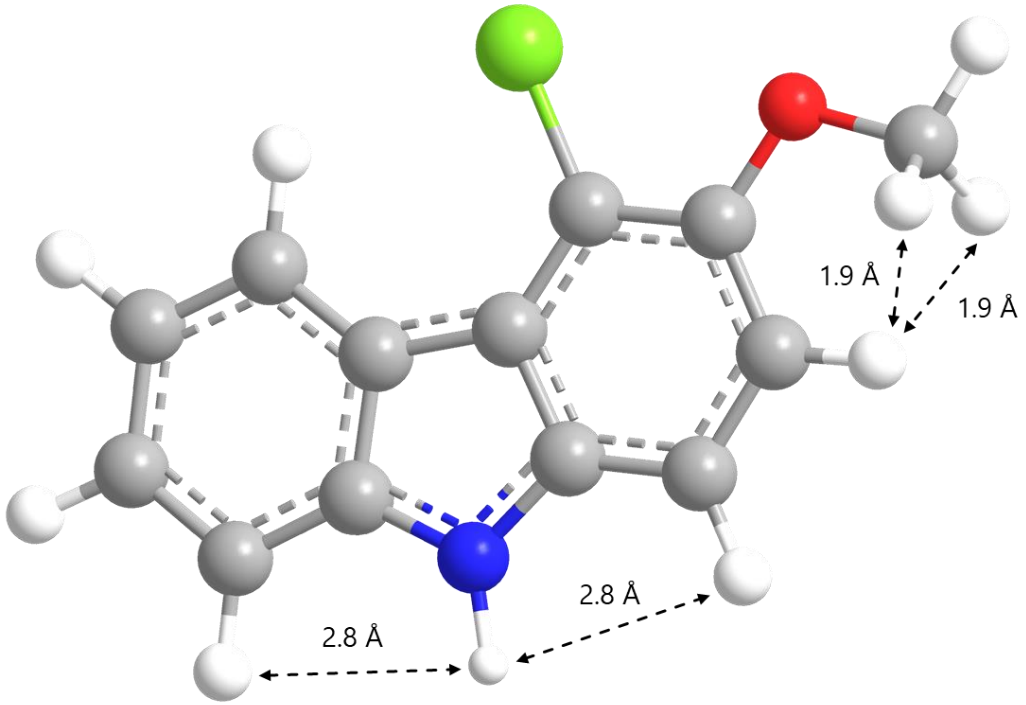

Figure S48. Distances measured between -NH/H1, -NH/H8, and H2/-OCH_3 using Chemdraw 3D (v21.0.0).

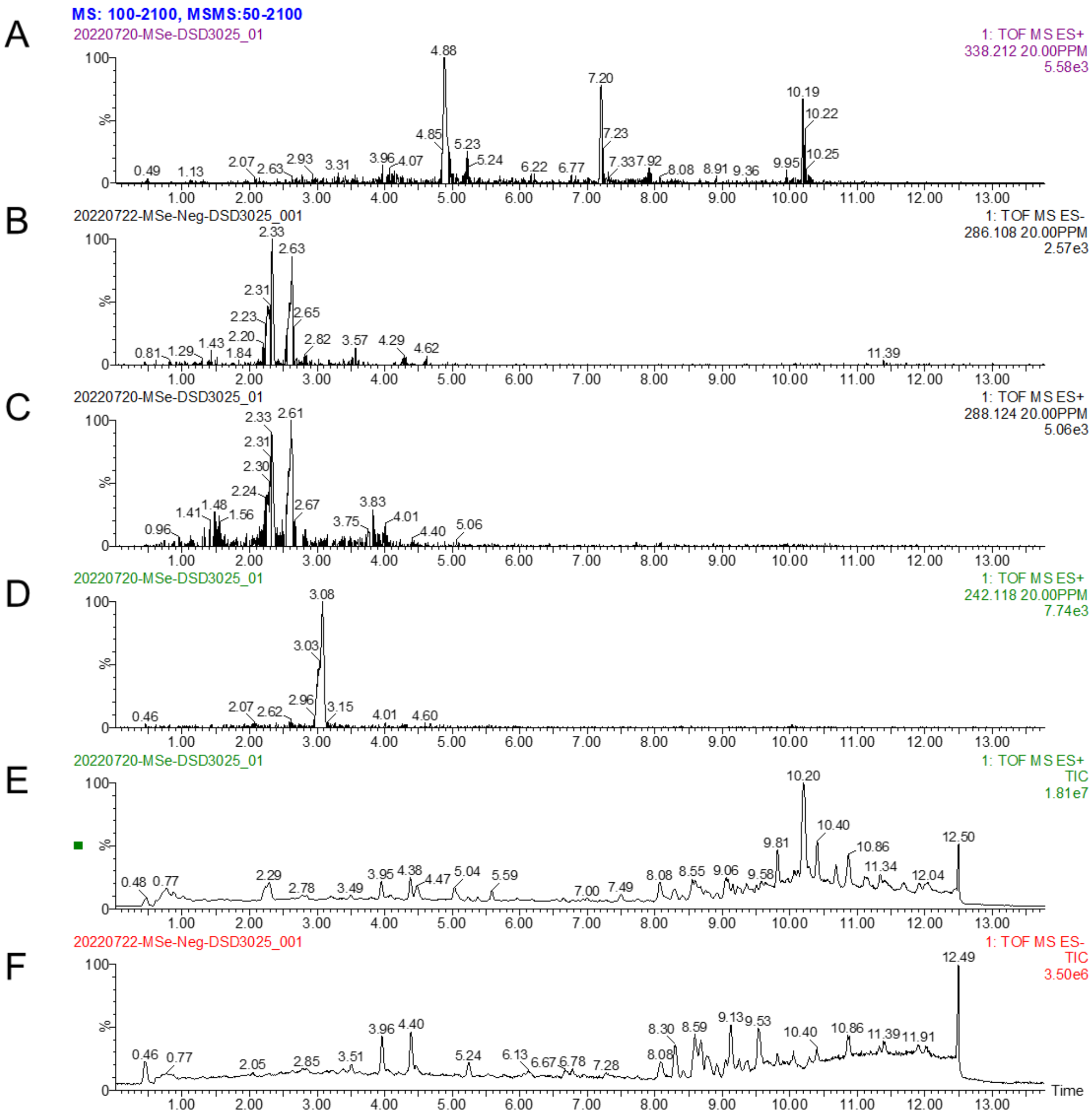

Figure S49. Detection of Non-Halogenated Carbazole Alkaloids in *S. tubbatahanensis* DSD3025^{T} Extract. Extracted Ion Chromatogram of (A) carbazoquinocin F in the positive mode, carbazomycin H in both (B) negative and (C) positive mode, and (D) carbazomycin B **8** in the positive mode were detected in *S. tubbatahanensis* DSD3025^{T} Extract

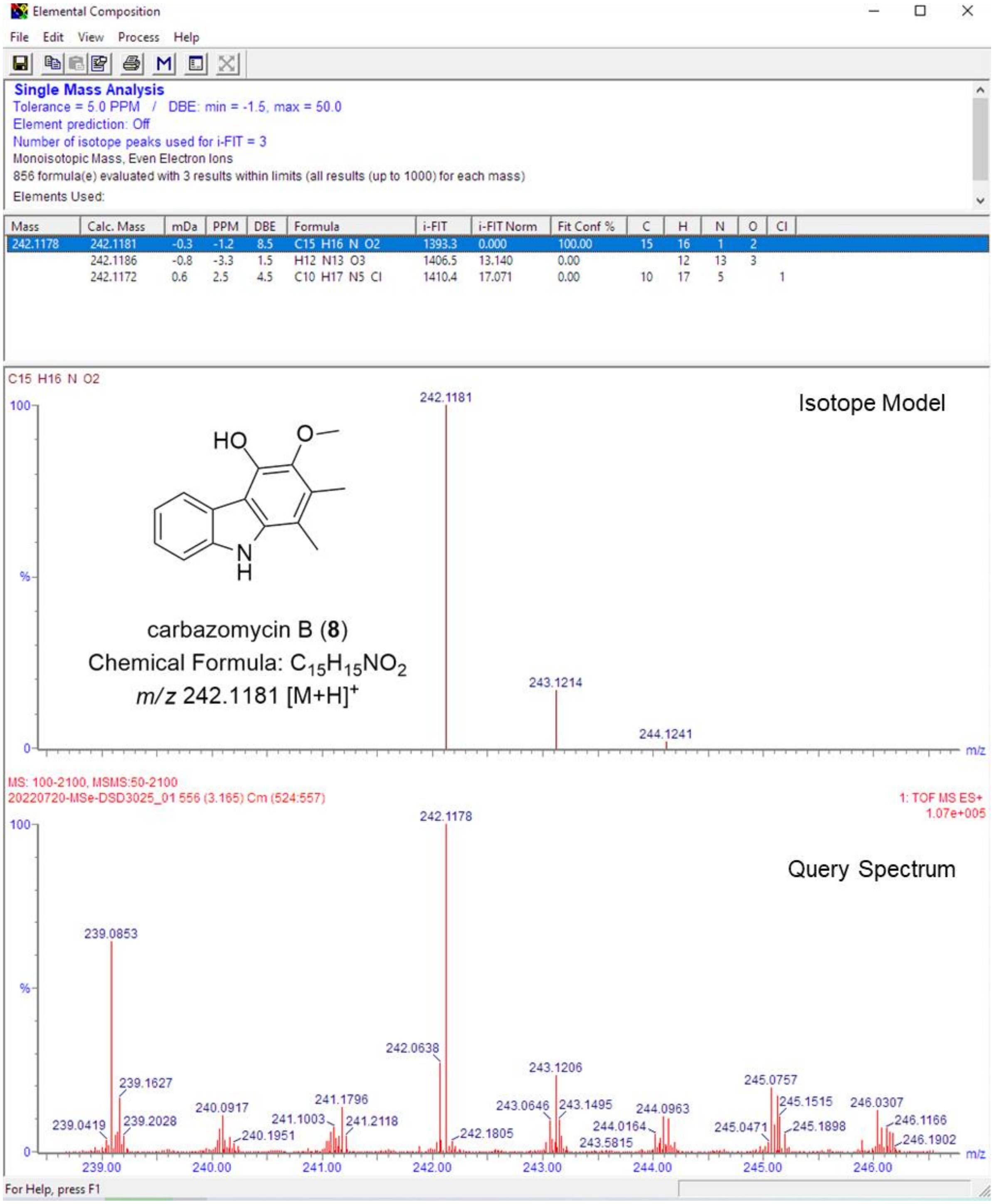

Figure S50. Formula prediction of compound **8** (*m/z* 242.1178 $[M+H]^+$) detected in the positive mode. Chemical formula was accurately predicted (<5.0 ppm) based on the measured mass, % fit confidence, and isotopic model. The mass ion peak at *m/z* 242.1178 $[M+H]^+$ was identified as carbazomycin B **8** with a neutral chemical formula of $C_{15}H_{15}NO_2$ and double bond equivalent of 9.

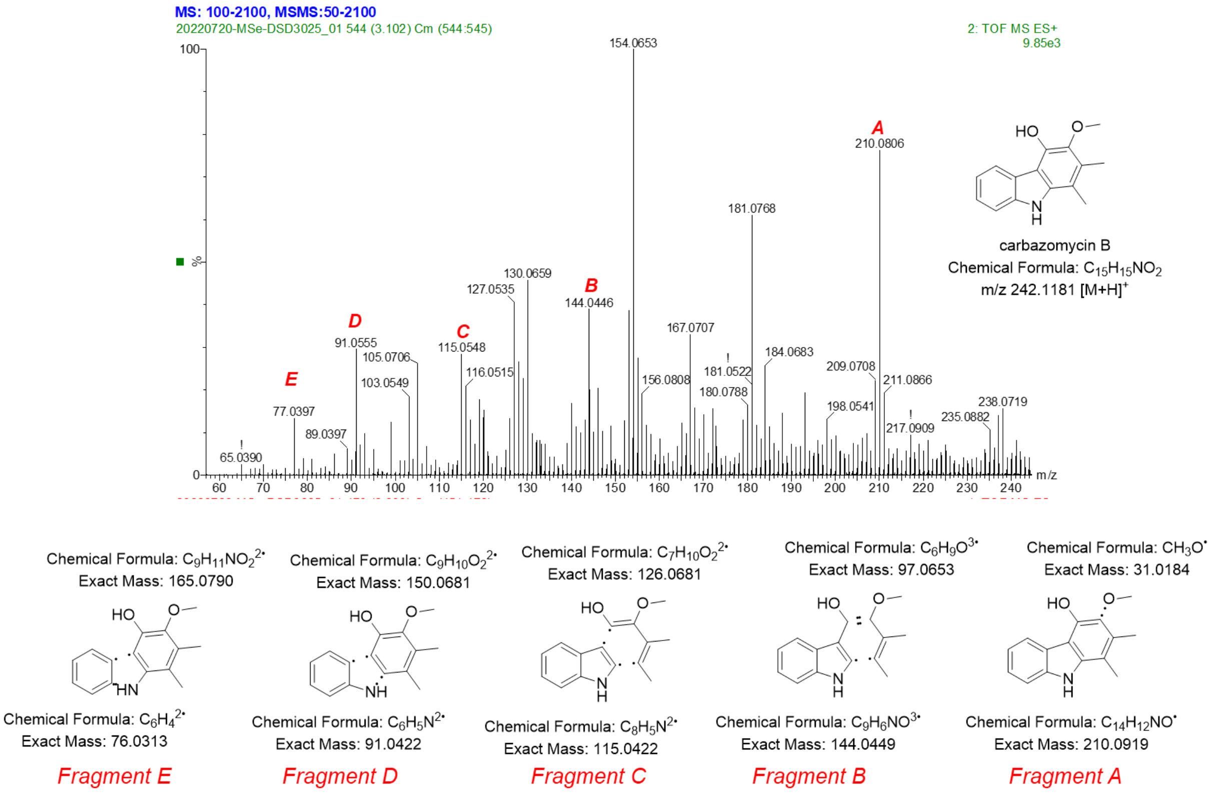

Figure S51. MS/MS Analysis of Carbazomycin B **8**. The MS/MS of carbazomycin B was extracted from the 2nd function of the MS^E data. Fragmentation of carbazomycin A matched the experimental MS/MS spectrum.

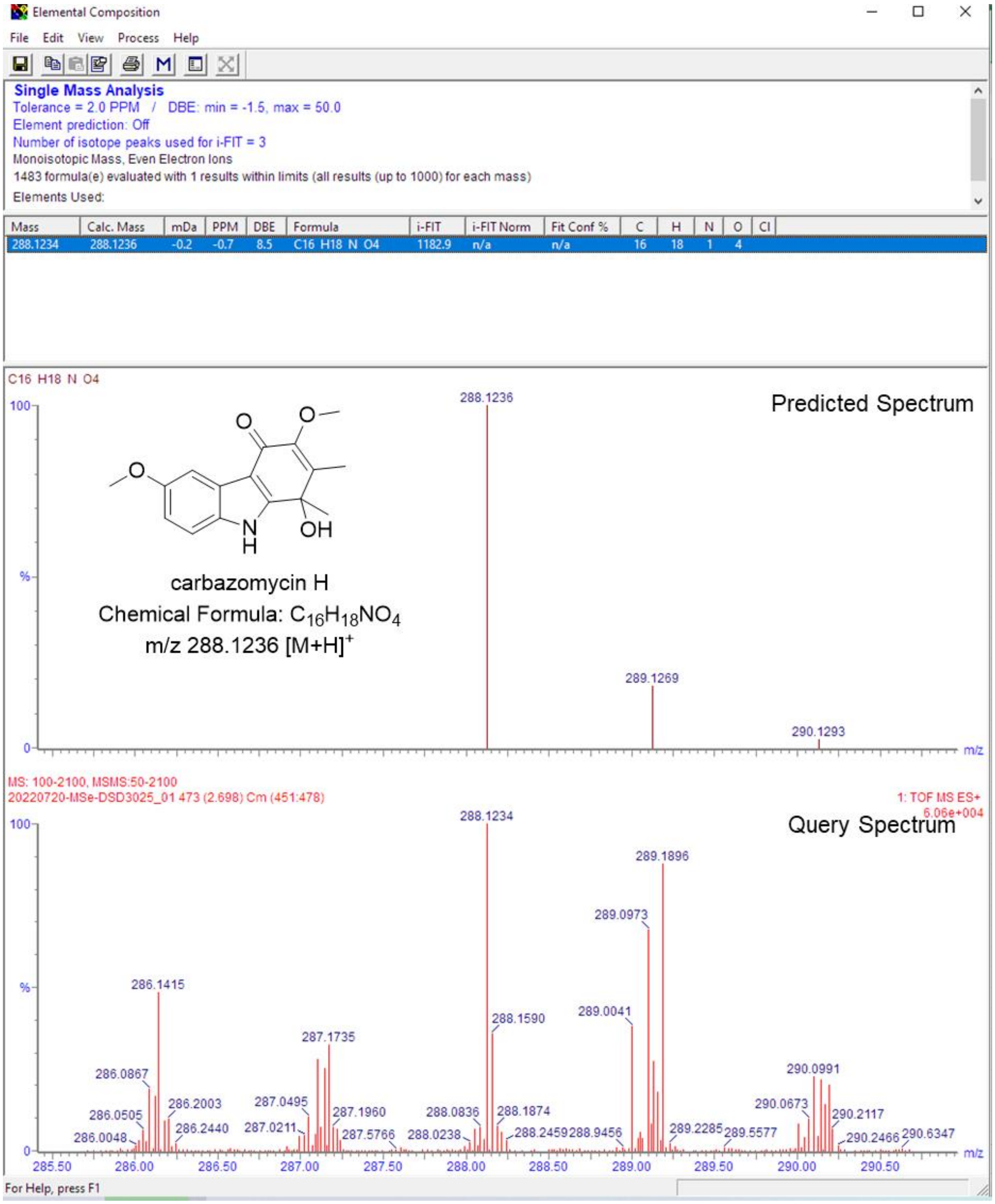

Figure S52. Formula prediction of mass ion peak detected in the positive mode at *m/z* 288.1234 $[M+H]^+$. Chemical formula was accurately predicted (<5.0 ppm) based on the measured mass, % fit confidence, and isotopic model. The mass ion peak at *m/z* 288.1234 $[M+H]^+$ was identified as carbazomycin H with a neutral chemical formula of $C_{16}H_{18}NO_4$ and double bond equivalent of 9.

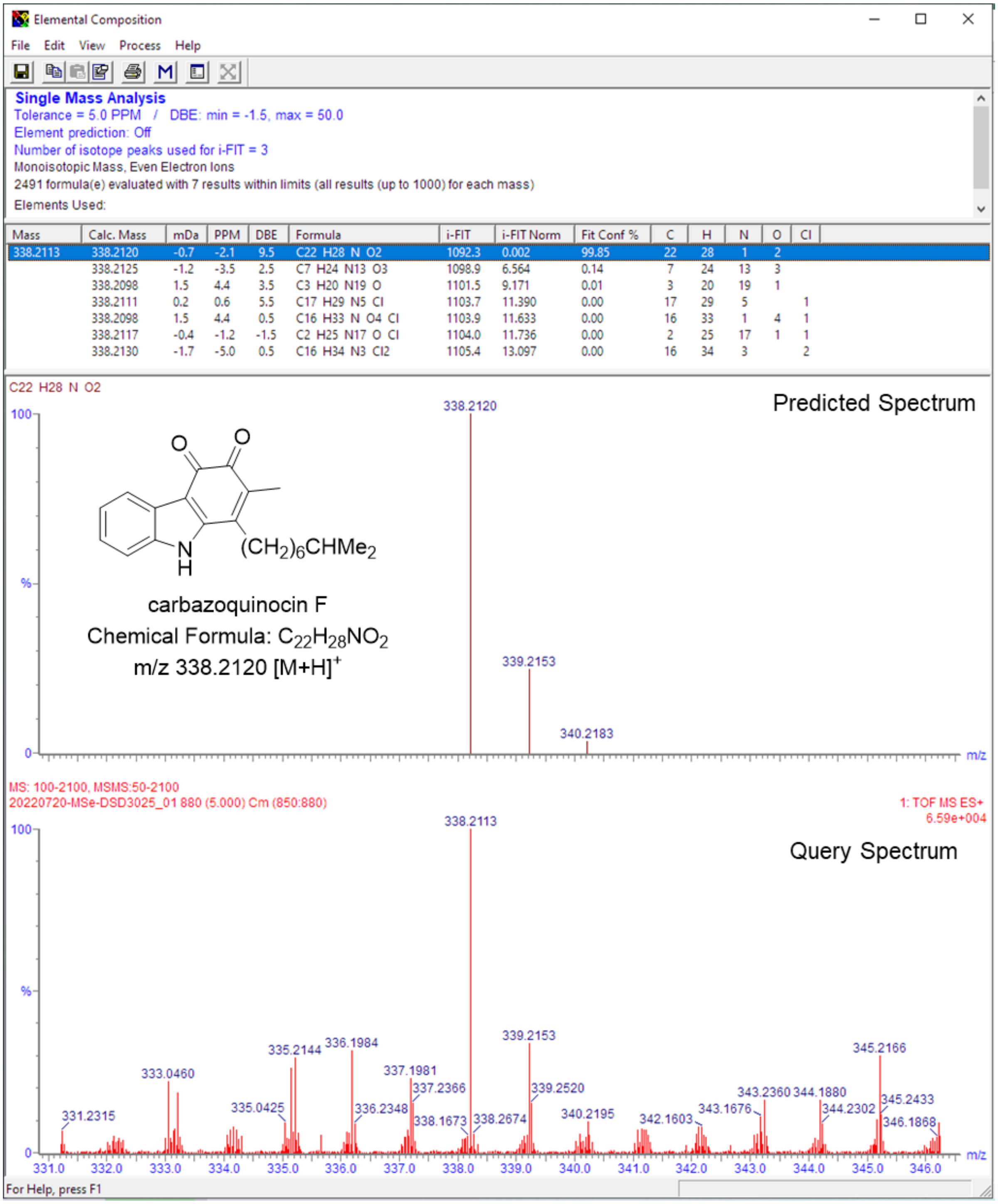

Figure S53. Formula prediction of mass ion peak detected in the positive mode at *m/z* 338.2113 $[M+H]^+$. Chemical formula was accurately predicted (<5.0 ppm) based on the measured mass, % fit confidence, and isotopic model. The mass ion peak at *m/z* 338.2113 $[M+H]^+$ was identified as carbazoquinocin F with a neutral chemical formula of $C_{22}H_{28}NO_2$ and double bond equivalent of 10.
